# Circulating tumor DNA to guide rechallenge with panitumumab in metastatic colorectal cancer: the phase 2 CHRONOS trial

**DOI:** 10.1038/s41591-022-01886-0

**Published:** 2022-08-01

**Authors:** Andrea Sartore-Bianchi, Filippo Pietrantonio, Sara Lonardi, Benedetta Mussolin, Francesco Rua, Giovanni Crisafulli, Alice Bartolini, Elisabetta Fenocchio, Alessio Amatu, Paolo Manca, Francesca Bergamo, Federica Tosi, Gianluca Mauri, Margherita Ambrosini, Francesca Daniel, Valter Torri, Angelo Vanzulli, Daniele Regge, Giovanni Cappello, Caterina Marchiò, Enrico Berrino, Anna Sapino, Silvia Marsoni, Salvatore Siena, Alberto Bardelli

**Affiliations:** 1 Department of Oncology and Hemato-Oncology, Università degli Studi di Milano (La Statale), Milan, Italy; 2 Department of Hematology, Oncology, and Molecular Medicine, Grande Ospedale Metropolitano Niguarda, Milan, Italy; 3 Medical Oncology Department, Fondazione IRCCS Istituto Nazionale dei Tumori, Milan, Italy; 4 Medical Oncology Unit 1, Veneto Institute of Oncology IOV-IRCCS Padua, Padua, Italy; 5 Candiolo Cancer Institute, FPO-IRCCS, Turin, Italy; 6 Department of Oncology, University of Turin, Turin, Italy; 7 IFOM, FIRC Institute of Molecular Oncology, Milan, Italy; 8 Mario Negri Institute for Pharmacological Research-IRCCS, Milan, Italy; 9 Department of Services, Grande Ospedale Metropolitano Niguarda, Milan, Italy; 10 Department of Surgical Sciences, University of Turin, Turin, Italy; 11 Unit of Radiology, Candiolo Cancer Institute, FPO-IRCCS, Turin, Italy; 12 Department of Medical Sciences, University of Turin, Turin, Italy

**Keywords:** Translational research, Colorectal cancer

## Abstract

Anti-epidermal growth factor receptor (EGFR) monoclonal antibodies are approved for the treatment of *RAS* wild-type (WT) metastatic colorectal cancer (mCRC), but the emergence of resistance mutations restricts their efficacy. We previously showed that *RAS*, *BRAF* and *EGFR* mutant alleles, which appear in circulating tumor DNA (ctDNA) during EGFR blockade, decline upon therapy withdrawal. We hypothesized that monitoring resistance mutations in blood could rationally guide subsequent therapy with anti-EGFR antibodies. We report here the results of CHRONOS, an open-label, single-arm phase 2 clinical trial exploiting blood-based identification of *RAS*/*BRAF*/*EGFR* mutations levels to tailor a chemotherapy-free anti-EGFR rechallenge with panitumumab (ClinicalTrials.gov: NCT03227926; EudraCT 2016-002597-12). The primary endpoint was objective response rate. Secondary endpoints were progression-free survival, overall survival, safety and tolerability of this strategy. In CHRONOS, patients with tissue-*RAS* WT tumors after a previous treatment with anti-EGFR-based regimens underwent an interventional ctDNA-based screening. Of 52 patients, 16 (31%) carried at least one mutation conferring resistance to anti-EGFR therapy and were excluded. The primary endpoint of the trial was met; and, of 27 enrolled patients, eight (30%) achieved partial response and 17 (63%) disease control, including two unconfirmed responses. These clinical results favorably compare with standard third-line treatments and show that interventional liquid biopsies can be effectively and safely exploited in a timely manner to guide anti-EGFR rechallenge therapy with panitumumab in patients with mCRC. Further larger and randomized trials are warranted to formally compare panitumumab rechallenge with standard-of-care therapies in this patient setting.

## Abstract (web summary)

In a phase 2 trial, monitoring tumor resistance mutations in blood using liquid biopsies allowed rationally guided subsequent therapy with anti-EGFR antibodies in patients with colorectal cancer.

## Main

The 5-year relative overall survival (OS) of patients with stage IV mCRC is lower than 15%^1,2^. Over a decade ago, the use of targeted agents, such as the anti-EGFR antibodies cetuximab and panitumumab, was shown to improve survival of patients with *RAS* WT mCRC^3^. However, patients with initial benefit from EGFR blockade almost invariably develop resistance^4,5^. From a molecular perspective, acquired resistance to anti-EGFR treatment is mostly associated with two main mechanisms: the first involves the emergence of activating mutations in *EGFR* downstream effectors (primarily, *NRAS* and *BRAF*), whereas the second relies on mutations in the *EGFR* extracellular domain (ECD) that impair antibody binding to its target^5–8^.

In 2015, we and others reported that mutant *KRAS* clones, which emerge in blood during EGFR blockade, decline upon withdrawal of anti-EGFR antibodies, indicating that clonal evolution continues beyond clinical progression^9–12^. Interestingly, *RAS* and *EGFR* ECD clones have been reported to decay in ctDNA with a half-life of 3.7 months and 4.7 months, respectively^11^. The decay of *KRAS* mutations in blood upon anti-EGFR antibody withdrawal is an indication of clonal evolution during therapy^13^.

Treatment of patients who initially respond and then progress after therapeutic EGFR blockade with cetuximab or panitumumab remains an unmet clinical need for at least three reasons. First, the molecular bases of relapse are patient specific and difficult to define, as tissue biopsies at the time of progression cannot be systematically performed in clinical practice and have intrinsic risks^13^. Second, *KRAS*, *NRAS* and *EGFR* ECD acquired mutations, which occur in about 40% of the cases, are often highly heterogeneous, and the corresponding mutant proteins are difficult to target pharmacologically^3^. Third, late-line standard therapeutic options beyond second-line treatment in patients with mCRC are poorly effective and ridden with meaningful toxicities^14,15^. As a result, upon failure of upfront anti-EGFR blockade, patients with *RAS* and *BRAF* WT mCRC and no targetable alterations, such as *ERBB2* amplification^16^ or *NTRK* fusions^17^, usually undergo additional lines of standard cytotoxic regimens and/or anti-angiogenic drugs^18,19^. None of the latter options is currently based on a diagnostic molecular testing^18,19^. In this setting, retreatment with anti-EGFR monoclonal antibodies may be used as late-line therapy in clinical practice^20^. In this context, rechallenge is defined as retreatment with a therapeutic agent to which the tumor has responded and then progressed upon^21^. Empirical anti-EGFR rechallenge therapy has a 8–20% response rate and manageable toxicities^20,22,23^. Despite much promising retrospective data, liquid biopsies have never been used interventionally to time and tailor anti-EGFR rechallenge in patients with mCRC^20,22–24^. To fill this gap, we conceived CHRONOS, a multicenter, open-label, single-arm phase 2 trial of panitumumab anti-EGFR therapy rechallenge guided by prospective and interventional assessment of *RAS*, *BRAF* and *EGFR* ECD mutational status in ctDNA. To our knowledge, this is the first time that liquid biopsies have been prospectively used to define treatment choice with an anti-EGFR antibody in patients with mCRC.

## Results

### Treatment characteristics of patients enrolled in CHRONOS

**Fig. 1:**
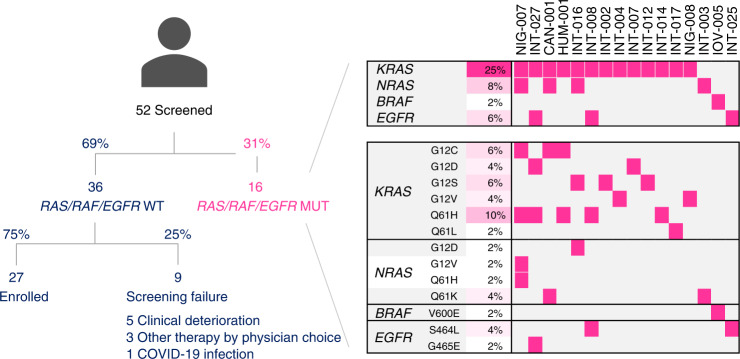
CONSORT diagram and molecular screening of the CHRONOS trial. Results of ctDNA ddPCR analysis and distribution of *RAS*, *BRAF* and *EGFR* ECD mutations in patients screened within the CHRONOS trial. Abbreviations: ctDNA, circulating tumor DNA; ddPCR, droplet digital PCR; ECD, ectodomain.

**Extended Data Fig. 1:**
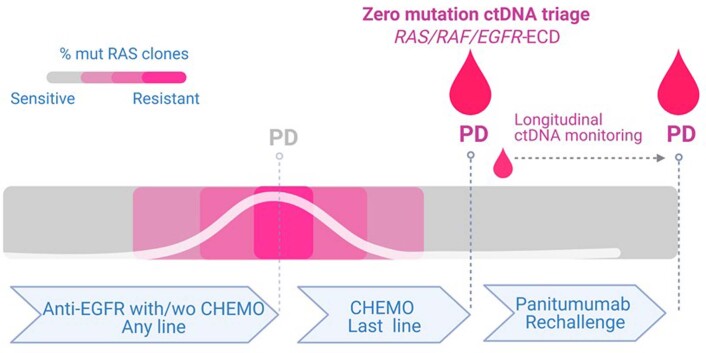
Schematic representation of the CHRONOS trial design (Amendment 3.0). Schematic representation of the CHRONOS trial design (Amendment 3.0). Created with BioRender.com.

**Extended Data Fig. 2:**
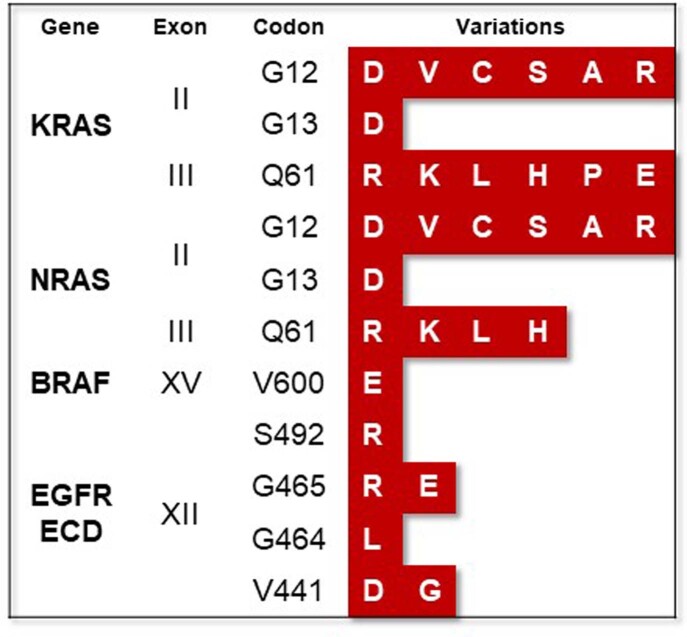
Molecular screening panel based on droplet-digital PCR implemented in the CHRONOS trial. Molecular screening panel based on droplet-digital PCR implemented in the CHRONOS trial.

CHRONOS was designed to test the hypothesis that assessing the presence/absence of resistance mutations in blood of patients with mCRC could be used to guide additional lines of anti-EGFR blockade (Fig. 1). To this end, we decided to enroll in CHRONOS only those patients in whom the ctDNA levels of all the mutations that we monitored were below detection, which we refer to as the ‘zero mutation ctDNA triage’ (Extended Data Fig. 1). This approach was set pragmatically to ensure timely and effective therapeutic intervention, considering the need to rapidly initiate the next round of treatment. A total of 52 patients were screened, and 27 with no detectable alterations in *RAS*, *BRAF* and *EGFR* ECD in ctDNA were enrolled for anti-EGFR treatment (Fig. 1 and Extended Data Fig. 2). Patient characteristics are representative of a third or further line population of patients with mCRC (Table 1). The median number of previous anti-cancer treatments was three, and the previous anti-EGFR treatment most commonly administered was panitumumab (15/27, 55%), predominantly in the first line (21/27, 78%) and always in combination with a cytotoxic backbone. All patients had a microsatellite stable (MSS) tumor. The primary endpoint of the trial was overall response rate (ORR) to panitumumab rechallenge according to Response Evaluation Criteria in Solid Tumors (RECIST) version 1.1 (ref. ^25^), and the secondary endpoints were progression-free survival (PFS), OS and toxicity according to Common Terminology Criteria for Adverse Events (CTCAE) version 4.03.

**Table 1:** Main clinicopathological features of patients enrolled in the CHRONOS trial

Characteristic	Study population
	(*n* = 27)
Age (median; range of years)	64 (42–80)
Gender (*n*, %)	
Male	16 (59)
Female	11 (41)
ECOG status (*n*, %)	
0–1	26 (96)
2	1 (4)
Primary tumor sidedness	
Right colon ^*^	5 (18)
Left colon ^§^	17 (63)
Rectum	5 (18)
Stage at initial diagnosis (*n,* %)	
Stage I–III	12 (44)
Stage IV	15 (56)
Mismatch repair status (*n*, %)	
MSI	0 (0)
MSS	27 (100)
Number of previous lines of therapy (median, range)	3 (2–6)
Oxaliplatin-containing regimens (*n*, %)	27 (100)
Irinotecan-containing regimens (*n*, %)	25 (93)
Anti-VEGF (*n*, %)	16 (59)
Regorafenib (*n*, %)	7 (26)
Trifluridine–tipiracil (*n*; %)	6 (22)
Previous anti-EGFR treatment	
Combination with chemotherapy (*n*, %)	27 (100)
- Previous rechallenge (*n*, %)	2 (7)
- Previous reintroduction (*n*, %)	7 (26)
Anti-EGFR monotherapy (*n*, %)	0 (0)
Type of previous anti-EGFR monoclonal antibody	
Panitumumab	15 (55)
Cetuximab	11(41)
Both	1 (4)

^*^ Located in caecum, ascending colon, liver flexure and transverse colon. ^§^ Located in splenic flexure, descending colon and sigmoid colon. VEGF, vascular endothelial growth factor; ECOG, Eastern Cooperative Oncology Group; MSS, microsatellite stable; MSI, microsatellite instable, EGFR, epidermal growth factor receptor.

### Blood detection of *RAS/BRAF* and *EGFR* ECD mutations

A panel of *KRAS*, *BRAF* and *EGFR* ECD mutations was assessed in ctDNA using a droplet digital PCR (ddPCR)-based assay (Methods). A total of 52 patients were screened; of these, 16 (31%) had at least one anti-EGFR resistance-conferring mutation in their ctDNA (Fig. 1). Precisely, we found that 13 of 52 (25%) patients had *KRAS*, four of 52 (8%) had *NRAS*, one of 52 (2%) had *BRAF* and three of 52 (6%) had *EGFR* ECD mutations in their ctDNA. Multiple mutations co-occurred in five of the 16 patients, whereas, in two patients, *BRAF* and *EGFR* ECD were the only resistance-conferring mutations, confirming that the addition of these two to the *RAS* panel optimized patient selection. The variant allele frequency varied between 0.28% and 46.20%. As we and others previously reported^5,12,26^, codon 61 *RAS* mutations were among the most frequently identified *RAS* alleles, with a prevalence of eight of 16 (50%) patients. The high frequency of Q61 variants in ctDNA of patients with mCRC is directly associated with secondary resistance to anti-EGFR antibodies; accordingly, these findings further confirm the emergence of EGFR blockade-specific mutations in CHRONOS^5,12,26^.

### Clinical outcome to panitumumab rechallenge

**Fig. 2:**
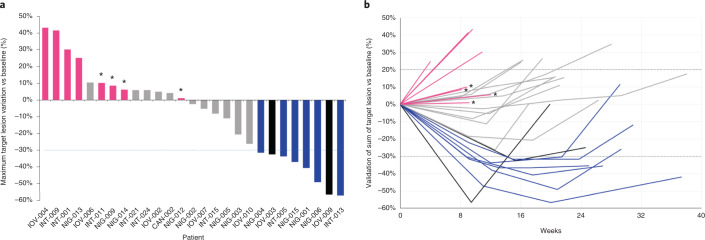
Waterfall plot depicts best responses to panitumumab rechallenge within the CHRONOS trial according to RECIST 1.1 (**a**). Spider plot displays best responses according to RECIST 1.1 and duration of response to panitumumab rechallenge (**b**). Magenta, progressive disease; gray, stable disease; blue, partial response; black, unconfirmed partial response; * progressive disease exclusively due to the onset of a new metastatic lesion.

**Extended Data Fig. 3:**
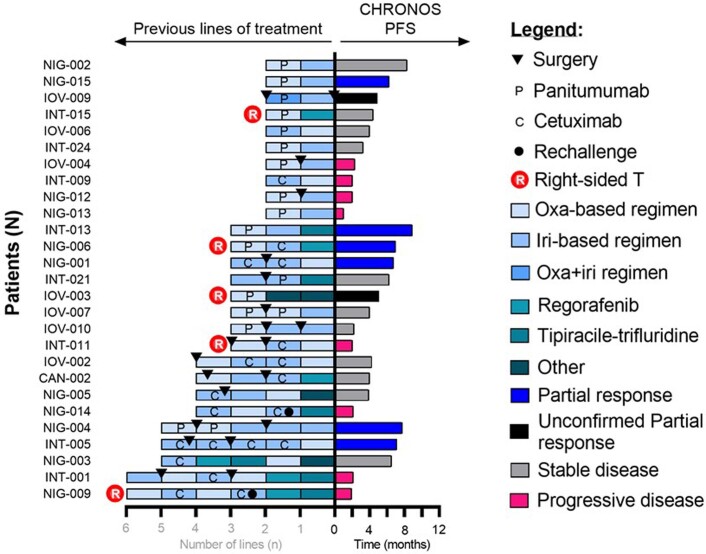
Outcome to panitumumab rechallenge in CHRONOS trial, according to (right) RECIST 1.1 response and progression-free survival and (left) the number and type of previous lines of treatments, type of previous anti-EGFR agent administered, and primary tumor sidedness. Outcome to panitumumab rechallenge in CHRONOS trial, according to (right) RECIST 1.1 response and progression-free survival and (left) the number and type of previous lines of treatments, type of previous anti-EGFR agent administered, and primary tumor sidedness. Created with GraphPad Prism 9.

**Extended Data Fig. 4:**
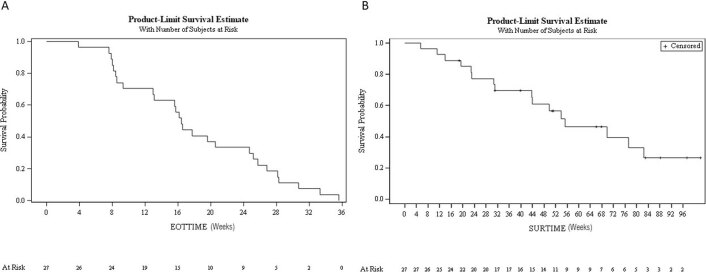
Progression-free survival (panel A) and overall survival (panel B) of the 27 patients enrolled in the CHRONOS trial. Progression-free survival (panel A) and overall survival (panel B) of the 27 patients enrolled in the CHRONOS trial. Keys: EOTTIME = End of Treatment Time; SURTIME = Survival Time.

According to the ‘zero mutation ctDNA triage’, 36 patients were molecularly eligible for panitumumab rechallenge. Of these, 27 received the drug as per trial protocol, six did not meet clinical inclusion criteria, and three were treated otherwise as per physician choice (Fig. 1). The median time interval between the screening liquid biopsy and the first dose of panitumumab rechallenge was 21 days (range, 9–44 days). The primary endpoint of the trial was met, with eight partial responses (PRs) (six confirmed plus two unconfirmed according to RECIST 1.1) defining an ORR of 30% (95% confidence interval (CI): 12–47%) (Fig. 2a). The median duration of response was 17 weeks (Fig. 2b). Stable disease (SD) was achieved in 11 of 27 (40%, 95% CI: 24–59%) patients and lasted more than 4 months in nine of 11 (82%) patients. A disease control rate (defined as PR plus SD >4 months) was, therefore, obtained in 17 patients (63%, 95% CI: 41–78%). Taking into account that patients had received, and failed, a median of three prior regimens of systemic treatments, the depth — in terms of percentage tumor shrinkage — and the dynamic of response achieved by single-agent panitumumab (Fig. 2) is remarkable and favorably compares with third-line standard-of-care treatments such as regorafenib or trifluridine–tipiracil^14,15,27^. Interestingly, objective responses occurred also in patients with right-sided primary tumors and in those heavily pretreated with more than four previous lines (Extended Data Fig. 3). A 16-week PFS further corroborates the response findings (Extended Data Fig. 4a and Fig. 2). The median OS was 55 weeks (Extended Data Fig. 4b). The anti-EGFR rechallenge was overall well-tolerated with manageable side effects as expected for this class of drugs. The safety analysis included all patients who received at least one dose of panitumumab. There were no permanent treatment interruptions due to adverse events nor treatment withdraws requested by patients. Supplementary Table 1 details all CTCAE grade treatment-related adverse events, including the five patients treated with panitumumab monotherapy according to previous protocol version 2.1. No grade 5 adverse events were reported; grade 3/4 toxicities were observed in seven of 32 (22%) patients. The most common severe adverse events were skin rash (3/32 patients, 9%), folliculitis (2/32, 6%), paronychia (1/32, 3%) and dermatitis (1/32, 3%). Dose reduction and treatment delay was required in three of 32 (9%) patients due to G3 skin rash, folliculitis and skin rash and dermatitis. Of note, 12 of 32 (37%) patients had primary prophylaxis with tetracycline antibiotics as per clinical choice, but the number of cutaneous events did not differ from those who did not receive such treatment.

### Detection of resistance mutations in ctDNA

**Fig. 3:**
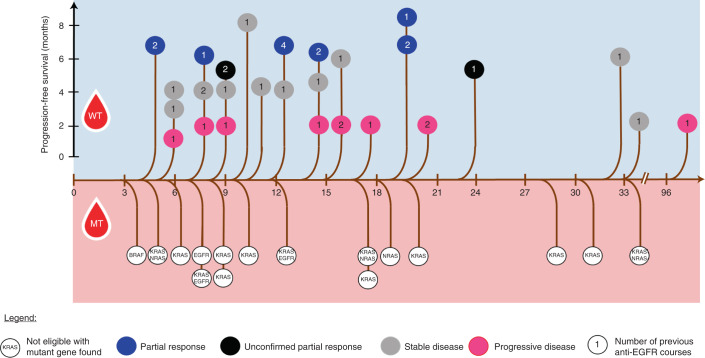
Tree graph describing ctDNA *RAS/BRAF/EGFR* status of patients screened for CHRONOS enrollment according to the time interval between the end of the last anti-EGFR course and the date of the CHRONOS ctDNA screening. Top: patients with WT sample; color-coded objective response to rechallenge with panitumumab on a time scale displaying PFS. Bottom: patients with mutated sample and mutations retrieved leading to CHRONOS screening failure. αEGFR, anti-EGFR. Abbreviations: MT, mutant; WT, wild-type.

It has been suggested that time thresholds based on mathematical modeling of the kinetic of decay of *RAS/EGFR* mutant clones can be used to define an optimal timing of rechallenge treatment with anti-EGFR antibodies in patients with mCRC^11^. In light of these findings, we calculated for each patient the screening time interval (STI), defined as the time intercurred between the dates of the last dose of previous anti-EGFR and that of the CHRONOS screening (Fig. 3). The median STI was 11.5 months. A specific time threshold differentiating patients with WT ctDNA from patients with mutated ctDNA was not present. Moreover, there was no correlation between length of STI and probability of response, further suggesting that ‘zero mutation ctDNA’ status is the main predictive factor for anti-EGFR rechallenge efficacy. Overall, these observations suggest that a predefined time limit^11^ might be ineffectual for a clinical selection of cases to be rechallenged, whereas ctDNA measurement enables definition of potentially responding patients.

### Molecular profiling before and after anti-EGFR rechallenge

**Fig. 4:**
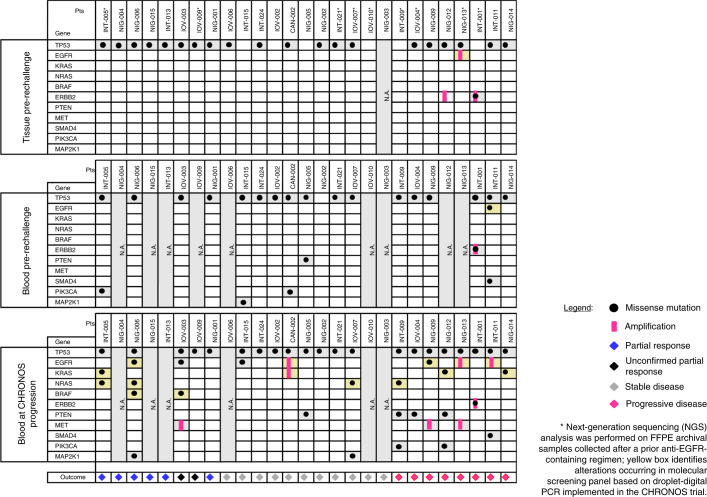
Alterations identified by NGS on tissue samples collected before CHRONOS enrollment (upper panel), on ctDNA at baseline to panitumumab rechallenge (middle panel) and on ctDNA at progression to panitumumab rechallenge (lower panel). As per inclusion criteria, all patients enrolled achieved complete or partial response to prior anti-EGFR antibodies either as monotherapy or in combination with cytotoxic agents. Mutations in the genes (*NRAS/KRAS/BRAF/EGFR*) of the molecular screening panel and *KRAS/EGFR* amplifications are highlighted in yellow.

We studied archival formalin-fixed paraffin-embedded (FFPE) samples, gathered before panitumumab rechallenge, as well as ctDNA collected at baseline and at progressive disease (PD) to panitumumab rechallenge (Fig. 4). Tissue samples were analyzed using the next-generation sequencing (NGS) Oncomine Comprehensive Assay version 3 (OCAv3), whereas ctDNA samples were genotyped by a high-sensitivity NGS panel assay based on a custom Duplex Sequencing (DS) workflow (Methods). NGS analysis on archival FFPE samples obtained before panitumumab rechallenge revealed an *ERBB2* amplification in two patients (INT-001 and NIG-012), one of whom (INT-001) also harbored the hotspot *ERBB2* p.V777L mutation (Fig. 4). It was previously reported by us and others that *ERBB2* mutations confer resistance to EGFR blockade in CRC^28,29^. Of note, both INT-001 and NIG-012 had PD as best response to panitumumab rechallenge.

Although tissue was available in all but one of the enrolled patients, plasma collected (before panitumumab rechallenge within the CHRONOS trial) was sometimes limited, as it was used primarily for the screening ddPCR assays required to enroll patients in the trial. In most cases (18/27), there was sufficient ctDNA left to also perform high-sensitivity NGS panel analysis on plasma before rechallenge. Interestingly, this analysis revealed, in a subset of the patients, the presence of *MAP2K1*, *PTEN*, *SMAD4* and *PIK3CA* mutations previously associated with resistance to EGFR blockade and that were not captured by the ddPCR assay (Supplementary Table 2).

NGS panel analysis was also performed on all the patients (21/27, 78%) for whom plasma collected at PD after rechallenge was available in sufficient amounts. In this instance, NGS analysis revealed in most of the cases (15/21, 71%) at least one genetic alteration previously linked to resistance to EGFR blockade (Fig. 4). The most common mechanisms of resistance identified at panitumumab rechallenge were mutations or amplification in the *EGFR*, *KRAS* and *NRAS* genes, which were observed in ten of 21 (48%) patients. As expected, *KRAS* and *NRAS* mutations occurred frequently at position 61. *PTEN* nonsense mutations were found in four patients, and *MET* amplification was detected in three patients. Interestingly, in ten of 21 (48%) patients, at least two alterations putatively conferring resistance to anti-EGFR treatment were identified (Fig. 4 and Supplementary Table 2). Of five patients with evaluable ctDNA analysis and achieving PR, three displayed resistance-conferring mutations at PD (INT-005, NIG-006 and IOV-003) and achieved a PFS of, respectively, 28 weeks, 28 weeks and 21 weeks. Similarly, the two remaining ctDNA-evaluable patients showing PR but with no mutations on ctDNA at CHRONOS progression (IOV-009 and NIG-001) achieved a PFS of 20 weeks and 27 weeks, respectively (Fig. 4). Of note, resistance mutations were also frequently detected in plasma of patients who did not clinically respond to rechallenge therapy. Because the parallel NGS analyses performed on the same individuals before anti-EGFR rechallenge were negative (Fig. 4), the emergence of molecular alterations in the tumors of four of six patients (INT-009, IOV-004, NIG-009 and NIG-014) indicates that a biological pressure was applied by the anti-EGFR antibodies also on the tumors that did not benefit by CHRONOS therapy according to RECIST. In other words, cancer evolution upon EGFR blockade must have occurred also in those patients in whom radiological determination did not highlight a clinical response.

## Discussion

Over 30% of patients with mCRC are eligible for a third or further line of treatment throughout their continuum of care^27,30^. In this context, retreatment with drugs to which patients had been previously exposed is often adopted in clinical practice despite the lack of biomarkers and of conclusive clinical evidence^20^. In keeping with such empirical practice, rechallenge with anti-EGFR agents is deployed according to the described decay of resistance mechanisms^11^, its manageable toxicity profile and potential induction of tumor shrinkage^20,22,23^. This strategy is commonly used in clinical practice and is increasingly guided by extended and real-time molecular profiling, despite the lack of prospective data^18,20^. We and others previously proposed that ctDNA analysis of anti-EGFR resistance-conferring mutations could be used to direct the selection of patients in this context^9,13,22,23^.

CHRONOS is a phase 2 trial assessing the activity and safety of an anti-EGFR rechallenge strategy based on an interventional assessment of *RAS*, *BRAF* and *EGFR* ECD status in ctDNA. Considering the lack of association of clinical factors with the outcomes of anti-EGFR retreatment^31^, the use of liquid biopsy to guide patient selection has three main advantages. First, it avoids a potentially ineffective and toxic treatment in the approximately 30% of the patients who are known to carry resistance-conferring mutations owing to previous exposure to EGFR blockade. Second, it empowers treatment decision-making by selecting patients according to real-time monitoring of resistance in tumors using blood as a proxy, independently of pre-defined clinical criteria, such as number of previous therapies, sidedness of primary tumor or a specific timeframe constraint before rechallenge^11,31^. The latter strategy is based on the observation that the average half-life of *RAS/EGFR* mutant clones, estimated by mathematical modelling of retrospective data, is approximately 4.3 months, hence the recommendation to wait at least 8 months — the equivalent of two mutant clone half-lives — before rechallenging the patient^11^. Of relevance, we found, instead, that mutant circulating alleles were absent as early as 4 months in patients who achieved PR to rechallenge (Fig. 3), whereas resistance-conferring mutations were found in the ctDNA beyond 12 months of anti-EGFR-free interval, underlying the need of personalizing the rechallenge interval to the single tumor, which can be done only via liquid biopsy. Third, it appears to enrich for response. The 30% response rate observed with the CHRONOS chemotherapy-free regimen favorably compares with the response rates of 8% and 21% of two other clinically based studies using rechallenge combination with chemotherapy or immunotherapy in the same setting^22,23^. In short, the results of CHRONOS indicate that selecting patients based on ctDNA improves the therapeutic index of anti-EGFR rechallenge by excluding resistant cases otherwise neglected by clinical criteria and by adopting a less toxic, chemotherapy-free panitumumab monotherapy.

CHRONOS was initially conceived to longitudinally monitor the drop of resistant mutations in blood, as originally reported by us^9^ and subsequently confirmed by others^32,33^. However, in clinical practice, implementation of multiple blood tests to confirm decay of resistant mutations to prompt a rechallenge therapy would be overcostly and impractical. When the approach was indeed attempted in our clinics, the availability of sequential samples, often widely spaced in time between each other, and the time needed to wait for two longitudinal ctDNA measurements (to assess mutation decay) clashed with the need to rapidly initiate the next round of treatment, as clinically required in patients with advanced-line mCRC.

To bypass these issues, we decided to enroll in CHRONOS only those patients in whom the ctDNA resistance mutations were undetectable. We refer to this strategy as the ‘zero mutation ctDNA triage’. This pragmatic approach is easily implemented in outpatient clinics and ensures effective turnaround time for timely therapeutic intervention.

The CHRONOS strategy has limitations and can be further improved. First, the three-gene ddPCR panel molecular screening implemented in CHRONOS could be further refined. In this regard, future studies should consider screening of a larger panel of resistant variants in ctDNA to increase the therapeutic index of anti-EGFR monoclonal antibodies. For example, assessment of *MAPK* alterations or *ERBB2/MET* amplification should also be considered, as these are similarly known to confer resistance to EGFR blockade, although at lower prevalence in mCRC^29,34–39^. Second, the assessment of anti-EGFR resistance-conferring mutations on ctDNA requires a dedicated ctDNA analysis laboratory support. As liquid biopsies become more routinely deployed, this issue should progressively fade owing to the large number of certified laboratories that now offer rapid ctDNA testing^40,41^. Third, we are unable to precisely estimate whether stochastic mutational events could have affected the sensitivity of detecting variants by ddPCR or NGS. Fourth, despite the use of ctDNA-based selection, a formal comparison between retreatment versus later-line standard options is lacking despite the historically favorable results of the former. Although these results are addressed by ongoing trials^42,43^, considering that the activity and safety of panitumumab rechallenge favorably compares (especially regarding tumor shrinkage potential) with other standard-of-care therapies beyond the second line of treatment, the CHRONOS strategy could be readily deployed in the clinical setting^14,15,27,30^.

In summary, CHRONOS demonstrated (to our knowledge for the first time prospectively) that genotyping tumor DNA in the blood can be safely, effectively and conveniently incorporated in the management of patients with mCRC. We conclude that ctDNA analysis is an effective strategy to select patients with mCRC for panitumumab rechallenge by allowing the maximization of treatment efficacy and concomitantly sparing iatrogenic toxicities.

## Methods

### Study design

CHRONOS (EudraCT 2016-002597-12, NCT03227926) is an open-label, single-arm, multicenter, phase 2 trial designed to evaluate the efficacy of rechallenging with panitumumab a population of patients with *RAS/BRAF* WT mCRC selected on the basis of absence of *RAS*, *BRAF* and *EGFR* ECD resistance mutations in ctDNA at the actual moment of treatment initiation (Fig. 1 and Extended Data Fig. 1). The primary endpoint was ORR by RECIST version 1.1 with independent central review. Secondary endpoints were PFS, OS, safety and tolerability of panitumumab rechallenge.

#### Patient population

The main inclusion criteria were histologically confirmed mCRC with *RAS* and *BRAF* WT status of the primary colorectal cancer and/or related metastasis; objective response and subsequent documented progression upon a previous anti-EGFR-therapy-based regimen administered in any line of treatment; intervening anti-EGFR-free treatment; and selection on the basis of *RAS*, *BRAF* and *EGFR* ECD WT status in ctDNA at molecular screening after progression (within 4 weeks) to the last anti-EGFR-free regimen (Extended Data Fig. 2). Further criteria were age older than 18 years; Eastern Cooperative Oncology Group (ECOG) performance status score of 0–2; and measurable metastatic disease according to RECIST version 1.1 (ref. ^25^). The trial was conducted in accordance with the Declaration of Helsinki^44^ and adhered to international Good Clinical Practice guidelines. The protocol was approved by the local ethics committees of participating sites (Niguarda Cancer Center, Grande Ospedale Metropolitano Niguarda, Milan, Italy; Fondazione IRCCS Istituto Nazionale dei Tumori, Milan, Italy; Veneto Institute of Oncology (IOV)-IRCCS, Padua, Italy; Istituto di Candiolo, Fondazione del Piemonte per l’Oncologia, FPO-IRCCS, Candiolo, Italy; Policlinico Universitario Biomedico, Rome, Italy; HUMANITAS Research Hospital, Milan, Italy). All patients provided written informed consent to study procedures. For more details, see the full protocol detailed in Supplementary Material Appendix 1. Due to the outbreak of the Coronavirus Disease 2019 (COVID-19) pandemic, and the resulting travel restrictions established by the Italian government, in three patients treatment was partially delivered in a hospital different from the initial recruiting center. Finally, some patients were enrolled following protocol violations as detailed in Supplementary Material Appendices 2, 3 and 4.

#### Treatment and procedures

Panitumumab was given at 6 mg kg^−1^ by intravenous administration over 1 hour on day 1 every 2 weeks until disease progression. Dose was reduced or delayed in case of side effects according to the Panitumumab Summary of Product characteristics, as reported in the protocol (Supplementary Material Appendix 1). A liquid biopsy was collected on days −28 to 0 to define the eligibility to the rechallenge treatment. Subsequently, liquid biopsies were drawn at each cycle until progression. Tumor assessments were performed by local radiologists within 4 weeks before treatment start (baseline) and were repeated every 8 weeks according to RECIST version 1.1 thereafter until progression. Local tumor assessments were reviewed centrally by two radiologists (D.R. and A.V.) who read the computed tomography scans blinded using Telemis version 4.9 software to collect, store and guide the revision of the imaging results. The imaging review protocol and tumor assessment reconciliation report are included in Supplementary Material Appendix 5. Safety was continuously assessed and graded according to CTCAE version 4.03. (Protocol Supplementary Material Appendix 1). All patients provided written informed consent. Detailed inclusion and exclusion criteria are provided in Supplementary Material Appendix 3.

#### Statistical analysis and reproducibility

We used the A’Hern one-stage approach to calculate the sample size. For the primary objective of the trial, 27 patients were required to achieve a power of at least 85% to test the null hypothesis that the rate of response to panitumumab would be 10% or less versus the alternative hypothesis that the response rate would be 30% or more, at a one-sided alpha level of 0.05. Six objective responses were necessary to declare the study positive. Initially, only patients experiencing a ≥50% drop of *RAS/BRAF* plasma clones, determined by testing the ctDNA at prior anti-EGFR progression and at progression to the preceding chemotherapy, entered the trial. These screening criteria were rapidly modified due to logistics attrition and more recent retrospective clinical knowledge^9^, as discussed in detail above (Discussion). Accordingly, the protocol was amended to require a single determination of *RAS/RAF* and *EGFR* ECD WT ctDNA (Extended Data Fig. 2), obtained at progression of the immediately preceding chemotherapy (any type). However, the expected panitumumab rechallenge ORR remained the same—that is, 30%—requiring under the same alpha and beta assumption a sample size of 27 patients. Therefore, 27 patients were recruited in the amended protocol (version 3.0) and are here reported in both the safety and efficacy analyses. The five patients enrolled in the previous version of the protocol are not included in this analysis and will be reported separately.

Secondary endpoints were PFS and OS as well as safety, given that panitumumab is routinely administered to patients with CRC. Translational exploratory objectives were aimed at studying the molecular determinants of response and resistance to study treatment and included molecular characterization of longitudinal liquid biopsies collected during the treatment. Time-to-event variables were estimated using the Kaplan–Meier method, and the log-rank test was used for testing the null hypothesis of no difference among curves. Analyses were performed with SAS statistical software, version 9.4.

No data were excluded from the analyses. The trial was not randomized. The investigators were not blinded to allocation during experiments and outcome assessment.

#### Plasma sample collection

At least 10 ml of whole blood was collected by blood draw using EDTA as anticoagulant. Plasma was separated within 5 hours through two different centrifugation steps (the first at room temperature for 10 minutes at 1,600*g* and the second at 3,000*g* for the same time and temperature), obtaining up to 3 ml of plasma. Plasma was stored at −80 °C until ctDNA extraction.

### ddPCR analysis of ctDNA

cfDNA extracted from at least 4 ml of plasma was amplified using ddPCR Supermix for Probes (Bio-Rad) using *KRAS G12/G13* (1863506), *NRAS G12* (12001094), *KRAS Q61* (12001626) and *NRAS Q61* (12001006) ddPCR Multiplex Mutation Screening Kit, *BRAF V600E* (dHsaMDV2010027) and *NRAS G13D* (dHsaMDV2510526) single-plex assay (Bio-Rad) and *EGFR* ECD custom design probes (Bio-Rad). ddPCR was then performed according to the manufacturer’s protocol, and the results were reported as the percentage or fractional abundance of mutant DNA alleles to total (mutant plus WT) DNA alleles. The theorical ddPCR limit of detection is one mutant in 20,000 WT molecules^45^. Notably, in blood samples, the detection of mutant molecules is also affected by the quality of the starting material. To increase the sensitivity of each experiment, cfDNA was isolated from at least 4 ml of plasma, and we considered unsuitable samples with fewer than 100 total ddPCR events. Next, 5–10 μl of DNA template was added to 10 μl of ddPCR Supermix for Probes (Bio-Rad) and 2 μl of the primer and probe mixture. Droplets were generated using the Automated Droplet Generator (Bio-Rad) where the reaction mix was added together with Droplet Generation Oil for Probes (Bio-Rad). Droplets were then transferred to a 96-well plate and then thermal-cycled with the following conditions: 10 minutes at 95 °C, 40 cycles of 94 °C for 30 seconds, 55 °C for 1 minute, followed by 98 °C for 10 minutes (ramp rate 2 °C s^−1^). Droplets were analyzed with the QX200 Droplet Reader (Bio-Rad) for fluorescent measurement of FAM and HEX probes. Gating was performed based on positive and negative controls, and mutant populations were identified. ddPCR data were analyzed with QuantaSoft analysis software (Bio-Rad) to obtain fractional abundance (F.A.) of the mutated or amplified DNA alleles in the WT or normal background. The quantification of the target molecule was presented as number of total copies (mutant plus WT) per sample in each reaction. F.A. is calculated as follows: F.A. % = (Nmut/(Nmut + Nwt) × 100), where Nmut is the number of mutant events, and Nwt is the number of WT events per reaction. Samples that showed positive events in the multiplex kit were afterwards tested for each single assay detected by the kit (*KRAS G12A*, *G12C*, *G12D*, *G12R*, *G12S*, *G12V* and *G13D*; *NRAS G12A*, *G12C*, *G12D*, *G12R*, *G12S* and *G12V*; and *KRAS* and *NRAS Q61H (183 A*>*C)*, *Q61H (183 A*>*T)*, *Q61K*, *Q61L* and *Q61R*). Each sample was analyzed in at least two technical replicates to validate the obtained results.

### NGS workflow and data generation on ctDNA samples

QIAmp MinElute ccfDNA Mini Kit (QIAGEN) was used for cfDNA extraction from plasma samples following the manufacturer’s protocol. Quantity and quality of cfDNA were evaluated by the Qubit dsDNA HS Assay Kit (Thermo Fisher Scientific) and 2100 Bioanalyzer with a High-Sensitivity DNA Assay Kit (Agilent Technologies), respectively.

Library preparation methods optimized for analysis of small targets and for identification of very low allelic frequency variations were applied. In particular, starting from published methods for the DS approach^46,47^, the protocol was specifically customized for cfDNA processing. Overall, based on the quality (highly fragmented) and the low quantity of starting material (from 35 ng up to 100 ng of input cfDNA), library preparation was performed with specific adjustments, whereas the target enrichment was customized with the design of a small target panel.

Library preparation started directly with end-repair reaction by using NEBNext End Repair Module (New England Biolabs) following the manufacturer’s protocol, therefore avoiding any fragmentation step. To minimize the loss of so short fragments (167–170 base pairs (bp))^9^ of low-input cfDNA, a clean-up step with an optimized ratio volume of AMPure XP beads (Beckman Coulter) was performed, without size selection. Furthermore, all clean-up washes foreseen in the workflow were performed maintaining samples/beads complex in the magnetic plate and using room temperature 80% (vol/vol) ethanol. For the subsequent dA-tailing reaction of blunt-ended DNA fragments, NEBNext dA-Tailing Module (New England Biolabs) was used, followed, again, by an optimized clean-up with AMPure XP beads (Beckman Coulter). At this point, quality and quantity for each sample were evaluated with 2100 Bioanalyzer with a High-Sensitivity DNA Assay Kit (Agilent Technologies). By using the so-obtained molarity for each cfDNA sample, the needed volume of DS adapters (for sequences and synthesis^46^) was calculated, considering an optimized ratio of 10:1 molar excess of DS adapters with respect to cfDNA. DS adapter ligation occurred with the use of T4 DNA Ligase Rapid Enzyme (600,000 U ml^−1^, Qiagen) with a longer incubation time with respect to the manufacturer’s instructions. The following AMPure XP beads-based clean-up step was improved to capture all DS adapter/cfDNA-ligated fragments and to wash out free adapters. DNA libraries, eluted in 1× TE Buffer Low EDTA (Affymetrix), were then evaluated by means of 2100 Bioanalyzer with a High-Sensitivity DNA Assay Kit (Agilent Technologies) to verify the proper ligation of DS adapters and to calculate the molarity of fragments of interest. At this point, because we updated the DS workflow for cfDNA processing, the yield we usually obtained in terms of attomoles was extremely lower than that suggested by the authors as necessary for proceeding^46^ on the bases of the target size of our custom panel. Therefore, the following step of PCR amplification for Duplex families generation was implemented, increasing the number of parallel reactions (ranging from four to six) and the number of PCR cycles (ranging from nine to 12), to obtain the best possible balance between the number of Duplex consensus families members and the total yield of amplified libraries. Duplicate families were obtained by using KAPA HotStart PCR Kit (250 U, Roche) and primers specific for the DS approach (for all oligo sequences^46^). The clean-up step was improved also in that case and was performed on pooled PCRs for each sample.

The target of interest was defined starting from the identification of genes (or specific exons) relevant for tumorigenesis, evolution and emergence of drug resistance in CRC. In detail, the genomic size of high-sensitivity capture LB panel (Liquid Biopsy panel, xGen Lockdown Probe Pools, IDT) is 59 kilobases for a total of 300 captured regions. The design includes hotspot regions of 44 target genes and all coding sequences of all isoforms of nine clinically relevant genes: *APC*, *BRAF*, *ERBB2*, *MET*, *TP53* and DNA damage response pathway genes (*MLH1*, *MSH2*, *MSH6*, *B2M* and *PMS2*). Moreover, tandem repeat sequences (ten loci) useful to determine the stability of microsatellite regions (MSS/MSI status) are included. Finally, the LB panel is embellished by a list of 55 single-nucleotide polymorphisms (SNPs) that were used to identify the allelic profile and to build the SNP identifier (SNP_ID) of each sample.

Before target enrichment and capture steps, fragment distribution patterns of post-family-PCR libraries were checked by means of 2100 Bioanalyzer with a High-Sensitivity DNA Assay Kit (Agilent Technologies), thus obtaining their concentration. Depending on the available material, a minimum of 1 µg (up to 3 µg) of post-PCR libraries was used for the LB panel target enrichment with xGen Hybridization and Wash Kit (Integrated DNA Technologies). The manufacturer’s protocol was followed, except for the choice to perform two rounds of enrichment/capture steps to increase the on-target capture. After the first round of target enrichment, a further amplification step was performed using the same primer oligos used for families PCR (for sequences, see ref. ^1^) and KAPA HiFi HotStart ReadyMix PCR Kit (Roche). All PCR-amplified material was then subjected to a second step of target enrichment/capture. A further amplification with KAPA HiFi HotStart ReadyMix PCR Kit (Roche) allowed reaching the amount required of final libraries and the insertion of individual index sequence (for sequences, see ref. ^46^) needed for the demultiplexing of NGS data. Final libraries were quantified by means of Qubit dsDNA BR Assay Kit (Thermo Fisher Scientific), and their fragment distribution was evaluated by the High-Sensitivity DNA Assay Kit (Agilent Technologies). Equal amounts of DNA libraries were pooled and sequenced using Illumina NextSeq 500 sequencer.

### Bioinformatic workflow for ctDNA analysis

All libraries were sequenced on NextSeq 500 sequencer (Illumina), and 150-bp paired-end READS were generated. The READS were processed using the described bioinformatic pipeline, which can be divided into five steps: (1) READS pre-processing; (2) tag parsing and initial alignment; (3) single-strand consensus sequence (SSCS) assembly; (4) duplex consensus sequencing (DCS) assembly; and (5) mutational calling. Points 2–4 were performed^46,47^ with the following customizations.

In the pre-processing step (1), to recover READS shorter than 150 bases, the READS length were checked, and, if necessary, ‘N’ bases were added at the end to uniform the READ length to 150 bases. Mates with homopolimeric sequences longer than 35 nucleotides (nt) in the first 50 nucleotides were removed. As final pre-processed step (1), READS were sorted based on 12-nt tag sequence. Pre-processed READS, at the beginning of the second step (2), were composed by 12-nt tag sequence followed by invariant 5-bp nucleotides, which corresponds to the ligation site. This invariant 5-bp was used as anchor to recognize the variable sequence (12-nt tag). READS with homopolimeric sequences greater than 9 bases in the tag sequence and regions with mismatched nucleotides in 5-bp anchor sequence were discarded. The two 12-nt tags present on each of the two paired-end reads were merged into a single 24-nt tag that was used as new ‘read name’ in the read header^46,47^. At the end of the process, the anchor sequence (the constant sequence of 5 bases at the beginning of the reads) was removed. In the third step (3), READS were aligned to the human genome reference version 19 (hg19) using BWA with standard option. READS sharing the same tag sequence and genomic coordinates were classified with a tag family (12-nt tag) and then grouped in a consensus (SSCS). In detail, a minimum of two members was required to build a tag family. The family members were then compared position by position (from the beginning to the end of each read), and the nucleotide was maintained in the SSCS only when at least 70% of the members were coherent. Genomic positions that could not form a consensus were considered undefined and replaced by an ‘N’ base in the SSCS. A maximum of 30% ‘N’ was permitted, and, if in a genomic position, more than 30% of the READS were ‘N’, in the SSCSs ‘N’ base was reported. At the end of this step, two SSCSs (one for each strand) were created with tag αβ (12 + 12 tag). (4) Two related SSCSs corresponding to the two initial DNA strands were grouped and compared position by position. Specifically, the two 12-nt sub-tag sequences α + β in the SSCS were associated with the complementary SSCS with tag β + α^46^. The 24-nt tag (α + β) of the DCS was derived by the association of each paired SSCS consisting of two matched 12-nt sequences (α and β) + (β and α). The tags of the matched SSCSs were grouped considering that each one corresponds to the transposed of the other. The paired-strand SSCSs were compared and only matching bases being kept producing the DCS. Non-matching bases were considered undefined and replaced by ‘N’ in DCS. DCSs containing more than 30% of ‘N’ were considered unsupported and discarded. In the last step (5), in order to all single-nucleotide variations, indels and copy number alterations, genetic discovery analysis was performed as previously reported^48,49^. Therefore, DCSs were mapped to hg19 using the BWA-mem algorithm with standard parameters. DCSs having more than seven mismatches compared to the human reference genome and bases with phred score <30 were filtered out. Mutations supported by only mismatch in the head/tail of the DCSs were discarded, and only mutations supported by more than two DCSs with minimum 3× depth (using three different DCSs) were considered in the final analysis. Pindel tool was used to call the short indel using DCSs. Indels supported by fewer than three altered DCSs were filtered out. Tumor focal gene copy number variations (CNVs) were calculated as the ratio of the median depth of the probe region and the median depth of all chromosomal regions in the panel. Copy number was considered increased when the log_2_ value was higher than 1 (refs. ^48,49^).

### NGS workflow and data generation on archival samples

Hematoxylin and eosin sections were cut from FFPE tumor blocks corresponding to a tumoral lesion of the 27 patients subjected to panitumumab rechallenge. These were archival samples collected before trial enrollment. In nine patients, the available lesion was collected after a prior anti-EGFR-containing regimen because samples used for eligibility to anti-EGFR prescription, as detailed in inclusion criterion 9, were not available due to tissue exhaustion for diagnostic procedures. For samples with adequate tumor cell content (26/27), four 8-µm-thick sections were microdissected, and DNA was extracted with the GeneRead DNA FFPE Kit (QIAGEN) and quantified with a fluorometric assay (Qubit, Thermo Fisher Scientific).

The OCAv3 target gene panel (161 genes) was applied to the available DNA purified from tumor FFPE samples. A total of 40 ng of DNA was subjected to library preparation using the Ion AmpliSeq Library kit Plus (Thermo Fisher Scientific) following the manufacturer’s instructions. Ion Xpress Barcode and Ion P1 Adapter (Thermo Fisher Scientific) were inserted during the library preparation. The amplicon-based libraries were quantified using the Ion Library TaqMan Quantitation Kit (Thermo Fisher Scientific), diluted to 50 pM and then sequenced. Template generation and chip loading were performed with the Ion Chef System (Thermo Fisher Scientific) by using the Ion 540 Kit-Chef, loaded to the Ion GeneStudioTM S5 Plus System for the sequencing (Thermo Fisher Scientific) for an expected mean read depth of 1,000×. BAM files derived from processed raw data were generated by the Ion Torrent platform-specific pipeline software (Torrent Suite Software 5.12, Thermo Fisher Scientific). All the BAM files were transferred on the Ion Reporter Software (version 5.10.5.0) (Thermo Fisher Scientific) and analyzed by the Oncomine OCAv3 w3.0–DNA–Single Sample (version. 5.10). The Ion Reporter workflow was applied to identify SNVs, indels and CNVs with a tumor-only pipeline using the parameters and the Boolean chain reported in Supplementary Table 3. Only those genes present also in the liquid biopsy panel were considered for downstream data analysis.

### Reporting summary

Further information on research design is available in the Nature Research Reporting Summary linked to this article.

## Online content

Any methods, additional references, Nature Research reporting summaries, source data, extended data, supplementary information, acknowledgements, peer review information; details of author contributions and competing interests; and statements of data and code availability are available at 10.1038/s41591-022-01886-0.

## Supplementary information

Supplementary Information Extended data figure captions and Supplementary Tables 1–3

Reporting Summary

## Data Availability

The following information and data are available: 1. Protocol: available in Supplementary Material Appendix 1. 2. Centralized peer revision of response: available in Supplementary Material Appendix 5. 3. Human sequencing data: fully anonymized data are available at the European Nucleotide Archive: https://www.ebi.ac.uk/ena/browser/home, code PRJEB49484. 4. Individual clinical data: Patient-related data not included in the manuscript and in the supplementary figures and materials were generated as part of a clinical trial and are subject to patient confidentiality according to the General Data Protection Regulation (2016/679). 5. Other raw data: Requests for data and materials other than the clinical data above and that can be shared (for example, tissue samples or imaging data) will need approval from the institutional review board of Fondazione del Piemonte per l’Oncologia FPO-IRCCS and should be addressed to Fondazione del Piemonte per l’Oncologia FPO-IRCCS, Strada Provinciale, 142 -KM 3.95 - 10060 Candiolo – Turin, Italy. All data shared will be de-identified.
