# Supplementary material for: Circulating tumor DNA to guide rechallenge with panitumumab in metastatic colorectal cancer: the phase 2 CHRONOS trial
Source: Nat Med. 2022 Aug 1;28(8):1612–8. doi: 10.1038/s41591-022-01886-0 (PMC9386661; doi:10.1038/s41591-022-01886-0)
Supplement: Supplementary file 1 — Extended data figure captions and Supplementary Tables 1–3 [file 41591_2022_1886_MOESM1_ESM.pdf]

nature portfolio

https://doi.org/10.1038/s41591-022-01886-0

Supplementary information

Circulating tumor DNA to guide rechallenge with panitumumab in metastatic colorectal cancer: the phase 2 CHRONOS trial

In the format provided by the authors and unedited

Supplementary Materials.

Supplementary Tables.

Supplementary Table 1. Treatment-related adverse events, including patients treated with panitumumab monotherapy according to protocol version 2.1 dated October 30th, 2017 (N=32).

Supplementary Table 2. Type of mutations and corresponding variant allele frequencies (VAFs) identified on circulating tumor DNA (ctDNA) from CHRONOS patients at baseline and progression to panitumumab monotherapy rechallenge.

Supplementary Table 3. Boolean Chain and filtering parameters for somatic variants detection (OCAv3 “tumor-only” pipeline).

Supplementary Material Appendices.

Supplementary Material Appendix 1. CHRONOS trial protocol v. 3.0.

Supplementary Material Appendix 2. Recruiting centers, principal investigators, and enrolled patients.

Supplementary Material Appendix 3. Inclusion and exclusion criteria Protocol version 3.0.

Supplementary Material Appendix 4. Protocol deviations.

Supplementary Material Appendix 5. Report of the independent review of radiologic responses of CHRONOS trial.

Supplementary Tables.

Supplementary Table 1. Treatment-related adverse events, including patients treated with panitumumab monotherapy according to protocol version 2.1 dated October 30th, 2017 (N=32).

**Event**	**Grades 1-2**	**Grade ≥ 3**
	Number of patients (percent) N=32 (100)	
Dermatological		
Rash	13 (41)	3 (9)
Folliculitis	9 (28)	2 (6)
Paronychia	7 (22)	1 (3)
Dry skin	4 (12)	0 (0)
Pruritus	4 (12)	0 (0)
Dermatitis	4 (12)	1 (3)
Erythrodysaesthesia	3 (9)	0 (0)
Hirsutism	3 (9)	0 (0)
Mucositis	3 (9)	0 (0)
Skin hyperpigmentation	1 (3)	0 (0)
Rhinitis	1 (3)	0 (0)
Ocular		
Conjunctivitis	6 (19)	0 (0)
Electrolytes alterations		
Hypomagnesemia	5 (16)	0 (0)
Hypokalemia	1 (3)	0 (0)
Gastrointestinal		
Diarrhea	1 (3)	0 (0)

Supplementary Table 2. Type of mutations and corresponding variant allele frequencies (VAFs) identified on circulating tumor DNA (ctDNA) from CHRONOS patients at baseline and progression to panitumumab monotherapy rechallenge.

Patient	Gene	Coord	NN.change	AA.change	Type	Blood pre-Rechallenge (VAF)	Blood at CHRONOS progression (VAF)
INT-005	TP53	chr17:7577538	c.G743A	p.R248Q	nonsynonymous	4,58	16,01
	KRAS	chr12:25398284	c.G35C	p.G12A	nonsynonymous	-	0,15
	PIK3CA	chr3:178936095	c.A1637G	p.Q546R	nonsynonymous	0,18	0,00
	KRAS	chr12:25380275	c.A183C	p.Q61H	nonsynonymous	-	0,18
	NRAS	chr1:115256529	c.A182T	p.Q61L	nonsynonymous	-	0,47
NIG-006	TP53	chr17:7577121	c.C817T	p.R273C	nonsynonymous	53,87	6,24
	EGFR	chr7:55228007	c.A1474T	p.S492C	nonsynonymous	-	0,16
	EGFR	chr7:55227924	c.C1391T	p.S464L	nonsynonymous	-	0,12
	NRAS	chr1:115256530	c.C181A	p.Q61K	nonsynonymous	-	0,86
	BRAF	chr7:140453136	c.T1799A	p.V600E	nonsynonymous	-	0,32
	MAP2K1	chr15:66727451	c.A167C	p.Q56P	nonsynonymous	-	0,03
IOV-003	TP53	chr17:7577539	c.C742T	p.R248W	nonsynonymous	0,19	0,37
	TP53	chr17:7576897	c.C949T	p.Q317*	stopgain	44,62	15,47
	EGFR	chr7:55228011	c.A1478T	p.N493I	nonsynonymous	-	0,07
	BRAF	chr7:140453136	c.T1799A	p.V600E	nonsynonymous	-	0,13
IOV-009	TP53	chr17:7577538	c.G743A	p.R248Q	nonsynonymous	NA	14,60
NIG-001	TP53	chr17:7577120	c.G818A	p.R273H	nonsynonymous	5,68	1,34
	TP53	chr17:7577547	c.G734A	p.G245D	nonsynonymous	0,39	0,25
	TP53	chr17:7578403	c.G527A	p.C176Y	nonsynonymous	0,14	-
IOV-006						NA	NA
INT-015	TP53	chr17:7577515	c.A766G	p.T256A	nonsynonymous	0,04	0,11
	TP53	chr17:7578205	c.G644C	p.S215T	nonsynonymous	0,05	-
	TP53	chr17:7578393	c.T537A	p.H179Q	nonsynonymous	0,70	0,57
	MAP2K1	chr15:66727455	c.G171T	p.K57N	nonsynonymous	0,06	-
	EGFR	chr7:55228019	c.G1486A	p.E496K	nonsynonymous	-	0,07
INT-024	TP53	chr17:7579470	c.G217C	p.V73L	nonsynonymous	2,98	10,15
	TP53	chr17:7578406	c.G524A	p.R175H	nonsynonymous	-	1,29
	TP53	chr17:7577109	c.T829A	p.C277S	nonsynonymous	0,07	-
IOV-002	TP53	chr17:7579470	c.G217C	p.V73L	nonsynonymous	3,06	7,91
	MAP2K1	chr15:66774129	c.G605T	p.G202V	nonsynonymous	-	0,07
CAN-002	TP53	chr17:7578263	c.C586T	p.R196*	stopgain	7,63	28,30
	PIK3CA	chr3:178936095	c.A1637T	p.Q546L	nonsynonymous	0,06	-
NIG-005	TP53	chr17:7573982	c.G1045T	p.E349*	stopgain	27,10	17,59
	PTEN	chr10:89692904	c.C907T	p.R303*	stopgain	1,23	0,30
NIG-002	TP53	chr17:7578265	c.T584C	p.I195T	nonsynonymous	-	0,57
INT-021	TP53	chr17:7577120	c.G818A	p.R273H	nonsynonymous	0,14	0,53
	TP53	chr17:7578503	c.G427A	p.V143M	nonsynonymous	0,15	-
IOV-007	TP53	chr17:7578271	c.A578G	p.H193R	nonsynonymous	67,27	34,87
	NRAS	chr1:115256530	c.C181A	p.Q61K	nonsynonymous	-	2,12
	MAP2K1	chr15:66727453	c.A169G	p.K57E	nonsynonymous	-	0,18
INT-009	TP53	chr17:7578517	c.C413A	p.A138D	nonsynonymous	0,06	0,07
	TP53	chr17:7578513	c.G417C	p.K139N	nonsynonymous	0,30	0,69
	NRAS	chr1:115256529	c.A182T	p.Q61L	nonsynonymous	-	0,08
	PTEN	chr10:89692844	c.C847T	p.Q283*	stopgain	-	0,06
	PIK3CA	chr3:178936095	c.A1637T	p.Q546L	nonsynonymous	-	0,05
	TP53	chr17:7577499	c.G782A	p.S261N	nonsynonymous	-	0,12
	TP53	chr17:7577109	c.T829A	p.C277S	nonsynonymous	-	0,06
IOV-004	TP53	chr17:7577548	c.G733A	p.G245S	nonsynonymous	19,85	36,31
	PTEN	chr10:89720828	c.A1498T	p.K500*	stopgain	-	0,08
NIG-009	TP53	chr17:7577121	c.C817T	p.R273C	nonsynonymous	88,26	87,12
	EGFR	chr7:55227924	c.C1391T	p.S464L	nonsynonymous	-	0,02
NIG-012	TP53	chr17:7578263	c.C586T	p.R196*	stopgain	NA	29,34
	KRAS	chr12:25398284	c.G35C	p.G12A	nonsynonymous	NA	0,21
	PTEN	chr10:89717615	c.C1159T	p.Q387*	stopgain	NA	14,33
	PIK3CA	chr3:178951970	c.G3025T	p.G1009*	stopgain	NA	0,03
NIG-013	TP53	chr17:7577141	c.G797A	p.G266E	nonsynonymous	NA	69,17
INT-001	TP53	chr17:7578406	c.G524A	p.R175H	nonsynonymous	0,06	-
	TP53	chr17:7577545	c.A736G	p.M246V	nonsynonymous	-	40,73
	ERBB2	chr17:37881000	c.G2329T	p.V777L	nonsynonymous	84,07	85,93
INT-011	TP53	chr17:7578475	c.C455T	p.P152L	nonsynonymous	16,85	25,32
	SMAD4	chr18:48603032	c.C1333T	p.R445*	stopgain	1,42	2,32
	EGFR	chr7:55227924	c.C1391T	p.S464L	nonsynonymous	0,03	-
NIG-014	TP53	chr17:7577094	c.C844T	p.R282W	nonsynonymous	0,60	2,35
	KRAS	chr12:25398284	c.G35C	p.G12A	nonsynonymous	-	1,53

Supplementary Table 3. Boolean Chain and filtering parameters for somatic variants detection (OCAv3 “tumor-only” pipeline).

**Boolean Chain**	(Variant Type OR Variant Effect) AND CNV Somatic Confidence Range AND Allele Frequency AND (5000Exomes Global MAF(20161108) OR ExAC ENFAF(1) OR Minor Allele Frequency AND UCSC Common SNPs) AND Allele Read-Count
**Variant Type in**	SNV, INDEL, CNV
**Variant Effect in**	missense, nonframeshiftInsertion, nonframeshiftDeletion, nonframeshiftBlockSubstitution, nonsense, stoploss, frameshiftInsertion, frameshiftDeletion, frameshiftBlockSubstitution
**Allele Frequency**	0.1 <= VAF <= 0.95
**5000Exomes Global MAF(20161108)**	<= 1.0E-6
**ExAC GMAF(1)**	<= 1.0E-6
**Global Minor Allele Frequency**	<= 1.0E-6
**Allele Read-Count**	100 <= ARC <= 100000
**Minimum Ploidy Gain (5% CI) over expected**	>= 5.0
**UCSC Common SNPs**	Not In
**Boolean Chain**	(Variant Type OR Variant Effect) AND CNV Somatic Confidence Range AND Allele Frequency AND (5000Exomes Global MAF(20161108) OR ExAC ENFAF(1) OR Minor Allele Frequency AND UCSC Common SNPs) AND Allele Read-Count
**Variant Type in**	SNV, INDEL, CNV
**Variant Effect in**	missense, nonframeshiftInsertion, nonframeshiftDeletion, nonframeshiftBlockSubstitution, nonsense, stoploss, frameshiftInsertion, frameshiftDeletion, frameshiftBlockSubstitution
**Allele Frequency**	0.1 <= VAF <= 0.95
**5000Exomes Global MAF(20161108)**	<= 1.0E-6
**ExAC GMAF(1)**	<= 1.0E-6
**Global Minor Allele Frequency**	<= 1.0E-6
**Allele Read-Count**	100 <= ARC <= 100000
**Minimum Ploidy Gain (5% CI) over expected**	>= 5.0
**UCSC Common SNPs**	Not In

Supplementary Material Appendix 1.

CHRONOS trial protocol v. 3.0.

A PHASE II TRIAL OF RECHALLENGE WITH PANITUMUMAB DRIVEN BY RAS CLONAL-MEDIATED DYNAMIC OF RESISTANCE.

THE **CHRONOS** TRIAL

CHRONOS
Franz Ignaz GÜNTHER
1765-70

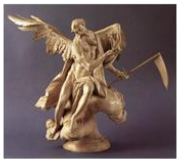

Limewood, white painted
Bayerisches Nationalmuseum
Munich

Study Type:	Phase II
Protocol Number:	013-IRCC-10IIS-16
EudraCT Number:	2016-002597-12
Protocol Version (Date):	v. 3.0 -22.03.2019
Sponsor:	Fondazione del Piemonte per l'Oncologia - Istituto di Candiolo IRCCS

This document contains confidential information belonging to Sponsor. Except as may be otherwise agreed to in writing, by accepting or reviewing these materials, you agree to hold such information in confidence and not to disclose it to others (except where required by applicable law), nor to use it for unauthorized purposes. In the event of actual or suspected breach of this obligation Sponsor should be promptly notified.

Contents

Contents **2**
1 SIGNATURE PAGE **9**
2 STUDY PERSONNEL **10**
3 SYNOPSIS **11**
4 SCHEDULE OF EVENTS **17**
5 BACKGROUND AND STUDY RATIONALE **18**
5.1 Rationale **18**
6 OBJECTIVES AND ENDPOINTS OF THE TRIAL **20**
6.1 Objectives **20**
6.1.1 Primary Objective 20
6.1.2 Secondary Objectives 20
6.1.3 Translational Objectives 20
6.2 End-Points **20**
6.2.1 Primary End-point 20
6.2.2 Secondary End-point 21
6.2.3 Translational End-point 21
7 TRIAL DESIGN **21**
7.1 Patient Population **21**
7.2 Patients' selection process **22**
7.2.1 Screening Phase 22
7.2.2 Trial Phase 23
7.3 Enrolment **23**
7.4 Study logistical plan **23**
8 STUDY POPULATION **24**
8.1 Inclusion Criteria **24**
8.2 Exclusion Criteria **25**
8.3 Patients substitution criteria **26**
8.3.1 Severe Infusion reaction (Trial Phase only) 26
9 TREATMENT (TRIAL PHASE ONLY) **26**
9.1 Description of investigational product **26**
9.2 Pregnancy and contraception **28**
9.3 Treatment Plan **29**

9.3.1 Drug Administration ... 29
9.3.2 Premedication and patient monitoring during infusion ... 30
9.3.3 Duration of Therapy ... 30
9.4 Concomitant Therapy ... 30
9.5 Dose Adjustments ... 31
9.5.1 Panitumumab Dose Reductions ... 31
9.5.2 Criteria for Withholding a Dose of Panitumumab ... 31
9.5.3 Criteria for Re-treatment with Panitumumab ... 32
9.5.4 Dose Modification Schedule for Panitumumab ... 32
9.5.5 Delayed or Missed Doses ... 33
9.5.6 Discontinuation ... 34
9.5.7 Management of dermatologic toxicity and recommended concomitant treatment ... 34
10 CLINICAL EVALUATION, LABORATORY TESTS, FOLLOW-UP ... 34
10.1 Before Treatment Start ... 34
10.2 During Treatment ... 36
10.3 After the End of Treatment (Follow-Up) ... 36
11 EFFICACY ASSESSMENT ... 37
11.1 Definitions ... 37
11.2 Tumour assessments ... 38
11.2.1 Measurability of Tumour Lesions at Baseline ... 38
11.2.2 Tumour Response Evaluation ... 40
11.2.3 Date of Progression ... 42
11.2.4 Reporting of Tumour Response ... 42
11.3 Progression Free Survival ... 43
11.4 Overall Survival ... 43
12 SAFETY ASSESSMENT ... 43
12.1 Definitions for Adverse Event ... 43
12.2 Reporting procedures for Adverse Event ... 44
12.3 Reporting requirements for adverse event ... 45
12.4 Reporting requirements for complaints related to panitumumab ... 47
12.5 Recording Adverse Events in the Case Report Forms ... 47
12.5.1 Pre-existing Conditions ... 47
12.5.2 Procedures ... 48

12.5.3 Symptoms of Targeted Disease ... 48
12.5.4 Causality Assessment and Grading of Adverse Event Severity ... 48
12.5.5 Pregnancy Reporting ... 49
12.5.6 Overdose ... 50
12.5.7 Follow-up of Unresolved Adverse Events ... 50
13 TRANSLATIONAL ASSESSMENT ... 50
14 STATISTICAL CONSIDERATIONS ... 51
14.1 Sample Size ... 51
14.2 Statistical Analysis ... 51
14.2.1 Analysis Populations ... 51
14.2.2 Statistical Methods ... 52
15 QUALITY CONTROL AND QUALITY ASSURANCE ... 54
15.1 Monitoring ... 54
15.2 Auditing ... 54
16 DATA HANDLING AND RECORD KEEPING ... 54
16.1 Case Report Form (CRF) ... 54
16.2 Data Handling ... 55
16.3 Record Retention ... 55
17 ETHICAL CONSIDERATIONS ... 56
17.1 Institutional Review Board (IRB)/ Independent Ethics Committee (IEC) and Competent Authority (CA) ... 56
17.2 Ethical conduct of the trial ... 56
17.3 Informed Consent ... 56
18 LIABILITY AND INSURANCE ... 57
19 CONFIDENTIALITY OF INFORMATION AND PUBLICATION OF RESULTS ... 57
20 FIGURES ... 58
20.1 Figure 1 Mutated KRAS alleles emerge in circulating DNA during anti-EGFR therapy and decline when treatment is suspended ... 58
20.2 Figure 2 Re-challenge with EGFR specific antibodies in CRC cells and patients ... 59
20.3 Figure 3 Study Flow Chart ... 61
21 APPENDIX ... 62
21.1 Appendix A: References ... 62
21.2 Appendix B: minimum set of genes to be tested on tissue FFPE ... 63

21.3 Appendix C: ddPCR Panel for Molecular Screening 64
21.4 Appendix D: Liquid Biopsy Guidelines for Molecular Screening Phase 65
21.5 Appendix E: Liquid Biopsy Guidelines for Trial Phase 65

List of Abbreviations

5-FU	5-fluorouracil
ADR	Adverse Drug Reaction
AE	Adverse Event
ALT	Alanine Aminotransferase (Serum Glutamic-pyruvic Transaminase)
ANC	Absolute Neutrophil Count
aPTT	Activated Partial Thromboplastin Time
AST	Aspartate Aminotransferase (Serum Glutamic-oxaloacetic Transaminase)
AUC	Area Under the Curve
BML	Baseline Mutational Load
CEA	Carcinoembryonic Antigen
CI	Confidence Interval
CL	Clearance
CONSORT	Consolidated Standards of Reporting Trials
CR	Complete Response
CRC	Colorectal Cancer
CRF	Case Report Form
CRO	Clinical Research Organization
CT	Computerized Tomography
CTC	Common Terminology Criteria
CTCAE	Common Terminology Criteria for Adverse Events
Ct-DNA	Circulating tumor DNA
CTM	Clinical Trial Manager
DCL	Data Clarification List
dd-PCR	Digital Droplet PCR
DSUR	Development Safety Update Report
eCRF	Electronic Case Report Form
ECD	Extracellular Ectodomain
ECG	Electrocardiogram
ECOG	Eastern Cooperative Oncology Group
EE	Efficacy Evaluable
EGF	Epidermal Growth Factor
EGFR	Epidermal Growth Factor Receptor
FFPE	Formalin Fixed Paraffin Embedded
FPI	First Patient In
GCP	Good Clinical Practice
G-CSF	Granulocyte Colony Stimulating Factor

HR	Hazard Ratio
IC	Informed Consent
IEC	Independent Ethics Committee
IgG2	Immunoglobulin G2
ICH	International Conference on Harmonization
IML	Intermediate Mutational Load
INR	International Normalized Ratio
IRB	Institutional Review Board
ITT	Intention-to-treat analysis
IV	Intravenous
LDH	Lactate Dehydrogenase
LLN	Lower Limit of Normal
LPI	Last Patient In
LPLV	Last Patient Last Visit
LVEF	Left Ventricular Ejection Fraction
mAb	Monoclonal Antibody
mCRC	Metastatic Colorectal Carcinoma
MRI	Magnetic Resonance Imaging
MS	Molecular Screening
Msec	Milliseconds
MUGA	Multigated Acquisition
NCI	National Cancer Institute
NGS	Next Generation Sequencing
NYHA	New York Heart Association
ORR	Objective Response Rate
OS	Overall Survival
PET	Positron Emission Tomography
PD	Progressive Disease
PFS	Progression-Free Survival
PR	Partial Response
QW, Q2W, Q3W, Q8W, Q9W	Every Week, Every 2 Weeks, Every 3 Weeks, Every 8 Weeks, Every 9 Weeks
RBC	Red Blood Cell
RECIST	Response Evaluation Criteria In Solid Tumors
RML	Rechallenge Mutational Load
ROC	Receiver Operator Characteristic
SADR	Serious Adverse Drug Reaction

SAE	Serious Adverse Event
SAS	Statistical Analysis System
SD	Stable Disease
SE	Safety Evaluable
SPF	Sun Protection Factor
SUSAR	Suspect Unexpected Serious Adverse Reaction
TMF	Trial Master File
ULN	Upper Limit of Normal
WBC	White Blood Count / White Blood Cells
WKS	Weeks
WT	Wild Type

1 SIGNATURE PAGE

Sponsor Signature

Prof. Anna Sapino

Signature 22/03/2019 Date

Clinical Study Chair Signature

Prof. Salvatore Siena

Signature ASST Grande Ospedale Metropolitano Niguarda S.C. ONCOLOGIA FALCK NIGUARDA CANCER CENTER Dr. SALVATORE SIENA 22/03/2019 Date

Translational Study Chair Signature

Prof. Alberto Bardelli

Signature 22/03/2019 Date

Principal Investigator Agreement

I have read the Protocol entitled "A PHASE II TRIAL OF RECHALLENGE WITH PANITUMUMAB DRIVEN BY RAS CLONAL-MEDIATED DYNAMIC OF RESISTANCE" and I agree to conduct the study as detailed herein and in compliance with ICH Guidelines for Good Clinical Practice and applicable regulatory requirements. I will provide all study personnel under my supervision with all information provided by the Sponsor and I will inform them about their responsibilities and obligations.

Principal Investigator
(printed name, Institution, Department and location)

Signature Date

2 STUDY PERSONNEL

STUDY CHAIRS
**OPERATIONAL**<br>**Prof. Anna Sapino**<br>Fondazione del Piemonte per l'Oncologia – Istituto di Candiolo IRCCS<br>Telefono: 011 993 3887; Email: anna.sapino@ircc.it<br><br>**CLINICAL**<br>**Prof. Salvatore Siena**<br>Niguarda Cancer Center, Grande Ospedale Metropolitano – MILANO e Dipartimento di Oncologia ed Onco-Ematologia Università degli Studi di Milano<br>Telefono: 02 6444 2291; Email: salvatore.siena@ospedaleniguarda.it<br><br>**TRANSLATIONAL**<br>**Prof. Alberto Bardelli**<br>Fondazione Piemontese per la Ricerca sul Cancro (FPRC) – CANDIOLO (TO) e Dipartimento di Oncologia Università degli Studi di Torino<br>Telefono; 011 993 3548; Email: alberto.bardelli@ircc.it

PROJECT MANAGER
**Dr. Francesco Rua**<br>Fondazione del Piemonte per l'Oncologia, Istituto di Candiolo IRCCS **–** CANDIOLO (TO)<br>Telefono: 011 993 3203; Email: francesco.rua@ircc.it

PHARMACOVIGILANCE
**Dr.ssa Fiorenza Enrico**<br>Fondazione del Piemonte per l'Oncologia, Istituto di Candiolo IRCCS **–** CANDIOLO (TO)<br>Telefono: 011 993 3261; Email: farmacia@ircc.it

LAB CENTRALIZED LIQUID BIOPSY
**Dr.ssa Giulia Siravegna and Dr.ssa Benedetta Mussolin (Centralized Liquid Biopsy Unit)**<br>Fondazione del Piemonte per l'Oncologia, Istituto di Candiolo IRCCS **–** CANDIOLO (TO)<br>Telefono: 011 993 3812; Email: giulia.siravegna@ircc.it; benedetta.mussolin@ircc.it<br><br>**Dr.ssaTiziana Venesio**, **(Molecular Pathology Unit)**<br>Fondazione del Piemonte per l'Oncologia, Istituto di Candiolo IRCCS **–** CANDIOLO (TO)<br>Telefono: 011 9933547; Email: tiziana.venesio@ircc.it

BIOSTATICIAN
**Dr. Valter Torri**<br>Istituto Mario Negri – MILANO<br>Telefono: 02 390 141; Email: valter.torri@marionegri.it

3 SYNOPSIS

Protocol Number	013-IRCC-10IIS-16
Title	**A PHASE II TRIAL OF RECHALLENGE WITH PANITUMUMAB DRIVEN BY RAS CLONAL-MEDIATED DYNAMIC OF RESISTANCE**
Brief title	The **CHRONOS** Trial
Sponsor	Fondazione del Piemonte per l'Oncologia FPO-IRCCS
Clinical Phase	Phase II
Background and Study Rationale	Colorectal cancer (CRC) is the third most common cancer in the world and the second leading cause of cancer death in the United States and the European Union. In the last decade, substantial advances in the treatment of the metastatic disease (mCRC) have more than doubled overall survival (OS) from 12 months to 30 months due to the refinement of fluoropirimidine-based chemotherapy and the introduction of antiangiogenics and targeted therapies.<br>Pharmacologic blockade of the epithelial growth factor receptor (EGFR) with specific monoclonal antibodies, namely, cetuximab and panitumumab, represents the mainstay of tumour targeted therapy for mCRC in patients with tumors not harboring extended RAS pathway mutations (KRAS, NRAS, or BRAF). Such alterations, which constitutively activate typical EGFR downstream transducers, have been shown to trigger substitute survival pathways that bypass therapeutic blockade of EGFR signalling, thus abating the efficacy of anti-EGFR antibodies ("primary resistance"). Even when response to anti-EGFR therapy occurs in the context of appropriate molecular selection, acquired ("secondary") resistance inevitably arises in all cases. Our group has extensively studied this phenomenon and has shown that extended-RAS alterations are the principal culprit of anti EGFR acquired resistance, and that altered RAS clones decay upon anti-EGFR treatment withdrawal, while tumor cells regain sensitivity to anti EGFR treatment. We have also documented that ctDNA profiles of individuals who benefit from multiple challenges with anti-EGFR antibodies, exhibit pulsatile levels of mutant KRAS. Collectively, these results indicate that the CRC genome adapts dynamically to intermittent anti-EGFR drug schedules, and provide a molecular explanation for the efficacy of re-challenge therapies based on EGFR blockade. Our results also give experimental support to the empirical-based clinical benefit observed,

	following cetuximab or panitumumab rechallenge in two small series of originally KRAS exon 2 wild type mCRC patients.<br>We propose to assess the efficacy and safety of re-challenging with panitumumab RAS-extend wild type mCRC patients with ctDNA-confirmed secondary resistance to anti EGFR treatment, after progression on second or further lines chemotherapy. As proof-of-concept, patients will be blood monitored throughout their therapeutic itinerary for the presence of extended-RAS alterations and EGFR-ectodomain mutations by ctDNA determination (liquid biopsy). We also include in our ddPCR panel 7 different EGFR extracellular domain (ECD) mutations as they occur in 15-20% of patients who acquired resistance to anti-EGFR drugs.
Primary Objective	To evaluate the efficacy of Panitumumab re-challenge in RAS/RAF wild type metastatic colorectal cancer (mCRC), without plasmatic evidence of potentially resistant clones harbouring RAS or EGFR-ectodomain mutations.
Secondary Objective(s)	• To assess Progression Free Survival (PFS) and Overall Survival (OS) after panitumumab re-challenge.<br>• To determine the safety and tolerability of panitumumab re-challenge.
Translational Objective(s)	• To link the blood-presence of RAS-extended and EGFR extracellular (ECD) clones (if any) during panitumumab treatment with response and response duration.<br>• To describe by NGS the tumour ctDNA plasma landscape before and after panitumumab re-challenge.<br>• To determine potential associations, if existing, between different mutated ctDNA clones RAS-extended and EGFR extracellular (ECD) clones (if any) and response to panitumumab re-challenge.<br>• To retrospectively correlate (whenever possible) the decay kinetics of RAS-extended and EGFR extracellular (ECD) clones during anti-EGFR treatment holiday with response to panitumumab rechallenge.
Primary Endpoint	Overall response rate (ORR) to panitumumab according to RECIST v1.1.
Secondary Endpoint(s)	PFS; OS and Toxicity according to CTCAE version 4.03.

Translational endpoint(s)	Longitudinal extended RASmut ctDNA levels in plasma by ddPCR; plasma ctDNA landscapes by NGS Candiolo Panel at panitumumab baseline and progression.
Design	Open label, single-arm, multiple centers, Phase II trial. The trial has been designed to prove or disprove whether a rechallenge with panitumumab can achieve an objective response rate (ORR= CR+PR) of 30% or more in a population of RAS wild type mCRC patients selected on the basis of RAS extended clonal evolution in their plasma.
Population	Patients with metastatic colorectal cancer (mCRC), originally responsive to anti EGFR therapy, selected on the basis of RAS extended and EGFR ECD domain mutations absence in plasma.
Main Inclusion Criteria	1. Histologically confirmed diagnosis of metastatic colorectal cancer;<br>2. Age ≥ 18 years;<br>3. Written informed consent;<br>4. Documented WT RAS exons 2, 3 and 4 (KRas and NRas) and WT BRAF V600E for anti-EGFR treatment.<br>5. Complete or partial response to anti EGFR antibodies in any line-either received as monotherapy or in combination with chemotherapy;<br>6. Imaging documented progression while on therapy with a therapeutic regimen including anti-EGFR mAb;<br>7. Imaging documented progression at the last treatment regimen that must be anti-EGFR free;<br>8. Patient must be RAS and EGFR ectodomain wild type in a liquid biopsy performed no longer that 4 weeks after progression to the last anti-EGFR free treatment<br>9. FFPE sample used for eligibility to anti-EGFR prescription (see criteria 4) must be available for custom gene panel profiling (as described in appendix B). Otherwise if sample is not available, center must have already perfomed a genotyping on this tissue sample according to appendix B.<br>10. ECOG performance status ≤ 2;<br>11. At least one measurable tumor lesion as per RECIST v1.1. Lesions in previously irradiated areas or those that have received other loco-regional therapies (i.e. percutaneous ablation) should not be considered measurable unless there is clear documented evidence of progression of the lesion since therapy. Imaging must be performed maximum within 28 days prior to registration;<br>12. Normal organ functions;

	13. Negative serum pregnancy test within 1 week prior to the first study dose in all women of childbearing potential;<br>14. Subjects and their partners must be willing to avoid pregnancy during the trial. Male subjects with female partners of childbearing potential and female subjects of childbearing potential must, therefore, be willing to use adequate contraception;<br>15. Absence of any psychological, familial, sociological or geographical condition potentially hampering compliance with the study protocol and follow-up schedule; those conditions should be discussed with the patient before registration in the trial.
Main Exclusion Criteria	1. History of severe infusion reactions to monoclonal antibodies cetuximab or panitumumab;<br>2. Symptomatic or untreated leptomeningeal disease and symptomatic brain metastasis;<br>3. Clinically significant cardiac disease including:<br>a. congestive heart failure requiring treatment (NYHA grade ≥ 2), Left ventricular ejection fraction (LVEF) < 45% as determined by Multigated acquisition (MUGA) scan or echocardiogram;<br>b. history or presence of clinically significant ventricular arrhythmias or atrial fibrillation;<br>c. clinically significant resting bradycardia;<br>d. unstable angina pectoris ≤ 3 months prior to starting study drug;<br>e. acute myocardial infarction ≤ 3 months prior to starting study drug;<br>f. QTcF > 480 msec;<br>4. History of thromboembolic or cerebrovascular events within the last 6 months, including transient ischemic attack, cerebrovascular accident, deep vein thrombosis, or pulmonary embolism;<br>5. Patients with interstitial pneumonitis or pulmonary fibrosis;<br>6. Abnormal organ or bone marrow functions defined as:<br>a. Absolute neutrophil count < 1.5 x 10/L;<br>b. hemoglobin < 9 g/dL;

	c. alkaline phosphatase > 2.5 x upper normal limit (ULN), if liver metastases > 5 x ULN;<br>d. aspartate aminotransferase (AST)/ alanine aminotransferase (ALT) > 2.5 x ULN, if liver metastases > 5 x ULN;<br>e. bilirubin > 1.5 x ULN, if liver metastases > 2 x ULN;<br>f. serum creatinine > 1.5 x ULN and/or creatinine clearance ≤ 50 mL/min calculated according to Cockroft-Gault;<br>g. Patients with platelet count <100 x 10^9/L<br>7. Previous or concurrent second malignancy. Exceptions: adequately treated basal cell or squamous cell skin cancer; in situ carcinoma of the cervix, treated curatively and without evidence of recurrence for at least 3 years prior to study entry; or other solid tumor treated curatively and without evidence of recurrence for at least 3 years prior to study entry.<br>8.Patients with positive serology for HIV, HBV, HCV.<br>9. Patients with a history of severe or life threatening hypersensitivity to the active substance or to any of the excipients.
Treatments	Panitumumab 6 mg/kg in 100 cc 0.9% NaCl solution on Day 1 every two weeks by IV administration over 1 hour.
Sample Size	We used the A'Hern one-stage approach to calculate the sample size. For the primary objective of the amended protocol v.3.0, we will need to enroll 27 patients in order to achieve a power of at least 85% to test the null hypothesis that the rate of response to panitumumab would be 10% or less, versus the alternative hypothesis that the response rate would be 30% or more, at a one-sided alpha level of 0.05. Six objective responses are necessary to declare the study positive.<br>Protocol amendment number 2 restricts, while simplifying the eligibility criteria to patients with a negative liquid biopsy for extended RAS/RAF and EGFR ectodomain mutations, while eligibility in the prior version required a reduction of at least 50% of extended RAS/RAF positive clones. The expected Panitunumab rechallenge ORR remains however the same i.e. 30%, requiring under the same alfa and beta assumption a sample size of 27 patients. Therefore 27 patients need to be recruited in the amended protocol v3.0. Patients resulting positive to the Panel B will be analyzed by the intention-to-treat (ITT) approach

	and will be included in the efficacy analysis population (see statistical analysis paragraph 14.2).<br>In the previous protocol v. 2.1 dated 30.10.2017, four patients were enrolled with the previous criteria. All these patients left the trial for progression to panitumumab rechallenge at the first tumor assessment. These patients will be included in the Safety Evaluation Population.
Study Timeline	Expected Timeline Amendment #2 12 months<br>Planned FPI Amendment # 2 March 1 2019<br>Planned LPI Amendment # 2 March 1 2020<br>Planned LPLV Amendment # 2 March 1 2021<br>Total duration of the trial after its activation 42 months<br>Expected final report between May-June 2021.

4 SCHEDULE OF EVENTS

Assessment/Event	Pre study[1]	Cyle Q2wks Treatment day	End of treatment Visit [2]	FUP Visit[3]
	≤ 28 days	d 1 (day 15 = day 1 > cy 2)	> 4 wks from last TX	Q8 WKS
**Informed consent signed**	X			
**Blood Samples to determine the molecular eligibility for the trial**	X			
**History Demographics**	X			
**Panitumumab Administration**		X		
**FFPE availability (only if not already perfomed tissue genotyping according to appendix B)**	X			
**Performance Status**	X	X	X	X
**Physical exam**	X	X	X	X
**Concomitant meds**	X	Throughout the study		
**Adverse event evaluation**		Throughout the study		
**Hematology[4]**	X	X	X	
**Serum chemistry[5]**	X	X	X	
**Serology for HIV, HBV, HCV[6]**	X			
**Urinalysis**	X		X	
**CEA**		Q6 weeks		
**Serum or urine pregnancy test [7]**	X			
**ECG[8]**	X	Only if clinically indicated	X	
**LVEF**	X	Only if clinically indicated	X	
**Tumor assessment[9]**	X	Q8 weeks (+/- 3 days)	X	X
**Blood sample for molecular analysis during panitumumab rechallenge[10]**		X	X	X

1. Within 4 weeks of starting therapy. In case of anomalous values for Hematology, Serum chemistry, ECG and LVEF, urinalysis tests, the analyses must be repeated within 7 days before the date of treatment. Laboratory assessments performed within 72 hrs of study treatment will be considered good also for D1.

2. End of Treatment visit to be performed approximately 4 weeks after last treatment dose.

3. Follow-up visit for patients without documented PD at the time of treatment withdrawal: to be performed every approximately 8 weeks until PD or another anti-cancer therapy is initiated, whichever comes first. Evaluations to be performed according to clinical practice.

4. Hematology: hemoglobin, hematocrit, platelet count, total white blood cell count (WBC) and differential (neutrophil count, lymphocyte, monocyte, eosinophil, and basophil counts), red blood cell count (RBC). To be performed within 72 hours from the day reported on the schedule of assessment.

5. Serum Chemistry: sodium, potassium, chloride, bicarbonate, creatinine, calcium, albumin, total bilirubin, total protein, glucose, alkaline phosphatase, AST, ALT urea or BUN, INR, aPTT. To be performed within 72 hours from the day reported on the schedule of assessment.

6. Serology testing before treatment start for: Hepatitis B virus: HBsAg, antibodies against HBsAg, anti-hepatitis B core antibody (anti-HBcAb); Hepatiatis C virus: HCV antibody (anti-HCV);HIV testing: patients will be tested for HIV prior to the inclusion into the study. To Be done only if molecularly elegible to the rechallenge.

7. Within 1 week prior to the first study dose

8.12 lead electrocardiogram (ECG).

9.Tumor assessment will be performed every 8 weeks thereafter during treatment until PD; however, in order to fulfil RECIST, the FIRST occurrence of response MUST be reconfirmed after 28 days. Patients without documented PD at the time of treatment withdrawal should continue to have disease assessments done every approximately 8 weeks until PD or another anti-cancer therapy is initiated, whichever comes first.

10. Patients will be sampled at baseline (Day -7 to 1, prior to first panitumumab administration), every two weeks (d1 of each 2 weeks cycle) and at disease progression.

5 BACKGROUND AND STUDY RATIONALE

5.1 Rationale

Colorectal cancer (CRC) is the third most common cancer in the world and the second leading cause of cancer death in the United States and the European Union[1]. In the last decade, substantial advances in the treatment of the metastatic disease (mCRC) have more than doubled overall survival (OS) from 12 months to 30 months due to the refinement of fluoropirimidine-based chemotherapy and the introduction of antiangiogenics and targeted therapies[2].

Pharmacologic blockade of the epithelial growth factor receptor (EGFR) with specific monoclonal antibodies, namely, cetuximab and panitumumab, represents the mainstay of tumour targeted therapy for mCRC[3]. However, treatment with cetuximab or panitumumab, in front or second line combinations with a chemotherapy back-bone or as monotherapy in further lines, has resulted in limited clinical benefit when applied to molecularly unselected mCRC patients[4]. More substantial response and survival improvements have been obtained by excluding patients with tumours harboring extended RAS pathway mutations (KRAS, NRAS, or BRAF)[3]. Such alterations, which constitutively activate typical EGFR downstream transducers, have been shown to trigger substitute survival pathways that bypass therapeutic blockade of EGFR signaling, thus abating the efficacy of anti-EGFR antibodies ("primary resistance")[5-7]. Unfortunately even when response to anti-EGFR therapy occurs in the context of appropriate molecular selection, acquired ("secondary") resistance inevitably arises in all cases[5]. Several mechanisms of secondary resistance to anti EGFR targeted therapy have been unveiled by our and other, but the most prevalent by far is again associated with the emergence of RAS axis alterations in patients originally classified as RAS wild type[7,8]. Starting from that discovery, we exploited circulating tumor DNA (ctDNA) to longitudinally track EGFR wild type clonal evolution in patients while on treatment with EGFR-specific antibodies followed, at progression, by additional lines of chemotherapy administered alone or in combination with anti-angiogenic drugs, or monotherapy with the multikinase inhibitor regorafenib. By doing so we confirmed extended RAS genes alterations in the ctDNA of these patients as the prevalent mechanism of acquired resistance to EGFR blockade[9]. Most importantly, we also observed that emerging altered RAS clones declined upon withdrawal of EGFR-specific antibodies and remained below the limit of detection across subsequent lines of treatment (Fig. 1).

These findings led us to postulate that clonal evolution of tumor cell populations that survive treatment with EGFR-specific antibodies continues beyond the point of clinically established resistance.

To mechanistically evaluate this possibility, we studied in vitro CRC cells (DiFi) in which acquisition of resistance to cetuximab is accompanied by amplification of the KRAS gene[7,10]. To parallel withdrawal of EGFR blockade, which occurs when patients develop cetuximab resistance, two populations of resistant DiFi cells were cultured for 160 days in the absence of anti-EGFR antibody (Fig. 2a). Analogously to what we had observed in the blood of patients, KRAS copies declined significantly in both cell models (Student's t-test, $P \leq 0.01$) when the EGFR-specific antibody was suspended (Figs. 1 and 2). The cell populations that experienced antibody withdrawal regained partial sensitivity to cetuximab (Student's t-test $P \leq 0.001$), as compared to the population in which the drug pressure was maintained (Fig. 2a).

In summary, three important findings were revealed by the integrated body of our research. First, that extended-RAS alterations are the principal culprit of anti EGFR acquired resistance. Secondly, that altered RAS clones decay upon anti-EGFR treatment withdrawal, and cells regain sensitivity to anti EGFR treatment. Finally, that ctDNA profiles of individuals who benefit from multiple challenges with anti-EGFR antibodies exhibit pulsatile levels of mutant KRAS. Collectively, these results indicate that the CRC genome adapts dynamically to intermittent anti-EGFR drug schedules, and provide a molecular explanation for the efficacy of re-challenge therapies based on EGFR blockade. Our results also give experimental support to the empirical-based clinical benefit observed, following cetuximab[9] or panitumumab rechallenge[11,12] in two small series of originally KRAS exon 2 wild type mCRC patients.

We propose to assess the efficacy and safety of re-challenging with panitumumab RAS-extend wild type mCRC patients with ctDNA-confirmed secondary resistance to anti EGFR treatment, after progression on second line chemotherapy. As proof-of-concept, patients will be blood monitored throughout their therapeutic itinerary for the presence of extended-RAS alterations and EGFR-ectodomain mutations by ctDNA determination (liquid biopsy). We also include in our ddPCR panel

7 different EGFR extracellular domain (ECD) mutations as they occur in 15-20% of patients who acquired resistance to anti-EGFR drugs[13,14].

6 OBJECTIVES AND ENDPOINTS OF THE TRIAL

6.1 Objectives

6.1.1 Primary Objective

- To evaluate the efficacy of Panitumumab re-challenge in RAS/RAF wild type metastatic colorectal cancer (mCRC), without plasmatic evidence of potentially resistant clones[14] harbouring RAS or EGFR-ectodomain mutations.

6.1.2 Secondary Objectives

- To assess Progression Free Survival (PFS) and Overall Survival (OS) after panitumumab re-challenge.
- To determine the safety and tolerability of panitumumab re-challenge.

6.1.3 Translational Objectives

- To link the blood-presence of RAS-extended and EGFR extracellular (ECD) clones (if any) during panitumumab treatment with response and response duration.
- To describe by NGS the tumour ctDNA plasma landscape before and after panitumumab re-challenge.
- To determine potential associations, if existing, between different mutated ctDNA clones RAS-extended and EGFR extracellular (ECD) clones (if any) and response to panitumumab re-challenge.
- To retrospectively correlate (whenever possible) the decay kinetics of RAS-extended and EGFR extracellular (ECD) clones during anti-EGFR treatment holiday with response to panitumumab rechallenge.

6.2 End-Points

6.2.1 Primary End-point

- Overall response rate (ORR) to panitumumab according to RECIST v1.1.

6.2.2 Secondary End-point

- PFS; OS and Toxicity according to CTCAE version 4.03.

6.2.3 Translational End-point

- Longitudinal extended RAS mut ctDNA levels in plasma by ddPCR; plasma ctDNA landscapes by NGS Candiolo Panel at panitumumab baseline and progression.

7 TRIAL DESIGN

This is a hypothesis driven, open label, single-arm, multiple centers, Phase II trial. The trial has been designed to prove or disprove whether a rechallenge with panitumumab can achieve an objective response rate (ORR= CR+PR) of 30% or more in a population of RAS wild type mCRC patients selected on the basis of RAS extended clonal evolution in their plasma.

7.1 Patient Population

The study has been designed on the basis of three translational findings, as described in the background. Namely that: i) no more than 20% of newly metastatic CRC patients have a tumor “addicted” to EGFR that respond and then become resistant to anti EGFR therapy; ii) in a large majority of cases, resistance to anti-EGFR therapy is due to the emergence, under the anti EGFR treatment selective pressure, of a pre-existing RAS mutant cellular clone(s); iii) RAS-altered clones will spontaneously decay upon anti-EGFR treatment withdrawal (anti EGFR therapy ‘holiday’), and cells will regain sensitivity to a second anti EGFR treatment.
Accordingly, to select the right population, we assumed the following

- Within an extended RAS wild type population of first metastatic CRC patients, approximately 20% of cases will have EGFR addicted tumors.
- EGFR addicted patients will achieve an Objective Response (CR o PR) to first therapy containing an anti EGFR monoclonal antibody, which can’t, however, be dissected from the response to the primary chemotherapy.
- At least 70% of the addicted/responding patients will eventually develop a RAS and/or EGFR ECD mediated resistance.

- After a period of anti EGFR treatment holiday, the RAS mutational load (measured in the plasma ctDNA) in at least 60% of these patients should drop according to an exponential decay kinetic[15].
- Only patients showing absence of RAS and EGFR ectodomain mutations might benefit from a rechallenge with panitumumab.

7.2 Patients' selection process

Given the assumption, and due to the proof-of-concept nature of the trial, we adopted a patients' selection process consisting of i) a Screening Phase, and ii) a therapeutic Trial Phase. The correspondent study flow-chart is shown in Figure 3.

Only patients having received a previous regimen including anti EGFR therapy will be considered for the Screening Phase. It is expected that up to 75% of patients will have received panitumumab. Patients showing absence of RAS and EGFR ectodomain mutations at RML will be declared "molecularly eligible" for the Trial Phase.

7.2.1 Screening Phase

The purpose of the Screening Phase is to determine the "molecular eligibility" of the patients for panitumumab re-challenge. Once a patient has been identified "screenable", the treating physician will seek his/her Informed Consent.

During screening phase, patients will be liquid biopsied (LB) at one optional (BML) and one mandatory (RML) check-points and their ctDNA tested by ddPCR to monitor the emergency, and subsequent decay, of RAS altered clones or EGFR ectodomain mutated clones.

The RAS Baseline Mutational Load (BML) samples will be optionally collected after the last anti-EGFR and acknowledgment of the PD upon first anti-EGFR treatment. Patients progressing during or after subsequent lines of therapy will be retested at the Rechallenge Mutational Load (RML) checkpoint. Patients showing absence of RAS and EGFR ectodomain mutational load at RML will be declared "molecularly eligible" for the Trial Phase. At each checkpoint we will also collect the corresponding radio-imaging data.

7.2.2 Trial Phase

Patients resulting “molecularly eligible” at the RML checkpoint, having satisfied all other eligibility criteria, will be treated with panitumumab monotherapy at standard dose until documented radiological progression or unacceptable toxicity or any other reason (See Section 9.2.3) whichever comes first.

7.3 Enrolment

Only patients provided with a properly processed interventional liquid biopsy (RML) obtained at last chemotherapy (see 21.3) can be enrolled in CHRONOS. In addition, if not already performed, patients will assay within 3 months from the panitumumab rechallenge start, the negativity for a minimum set of gene mutations (defined in appendix B) assayed through a multiplex gene profiling platform (i.e. Foundation Medicine platform, Personal Genomic Diagnostic platform, MSK-IMPACT platform) at diagnosis of metastatic disease or at study screening.

7.4 Study logistical plan

Plasma ct-DNA determination by ddPCR and NGS will be undertaken by the Dept of Oncology, University of Torino, located at the Institute of Candiolo IRCCS. Tumor tissue that has not already been genotyped at participating center will be tested with the Panel of genes reported in Appendix B (performed through the following platform or equivalent: Foundation Medicine platform, Personal Genomic Diagnostic platform, MSK-IMPACT platform), including tumors of patients referred from non-participating centers; Radio-imaging will be collected, stored and centrally analyzed by the means of the mintLesions ® or equivalent software.

8 STUDY POPULATION

8.1 Inclusion Criteria

1. Histologically confirmed diagnosis of metastatic colorectal cancer;
2. Age ≥ 18 years;
3. Written informed consent;
4. Documented WT RAS exons 2, 3 and 4 (KRas and NRas) and WT BRAF V600E for anti-EGFR treatment.
5. Complete or partial response to anti EGFR antibodies in any line–either received as monotherapy or in combination with chemotherapy;
6. Imaging documented progression while on therapy with a therapeutic regimen including anti-EGFR mAb;
7. Imaging documented progression at the last treatment regimen that must be anti-EGFR free;
8. Patient must be RAS and EGFR ectodomain wild type in a liquid biopsy performed no longer that 4 weeks after progression to the last anti-EGFR free treatment
9. FFPE sample used for eligibility to anti-EGFR prescription (see criteria 4) must be available for custom gene panel profiling (as described in appendix B). Otherwise if sample is not available, center must have already perfomed a genotyping on this tissue sample according to appendix B.
10. ECOG performance status ≤ 2;
11. At least one measurable tumor lesion as per RECIST v1.1. Lesions in previously irradiated areas or those that have received other loco-regional therapies (i.e. percutaneous ablation) should not be considered measurable unless there is clear documented evidence of progression of the lesion since therapy. Imaging must be performed maximum within 28 days prior to registration;
12. Normal organ functions;
13. Negative serum pregnancy test within 1 week prior to the first study dose in all women of childbearing potential;

14 Subjects and their partners must be willing to avoid pregnancy during the trial. Male subjects with female partners of childbearing potential and female subjects of childbearing potential must, therefore, be willing to use adequate contraception;

15 Absence of any psychological, familial, sociological or geographical condition potentially hampering compliance with the study protocol and follow-up schedule; those conditions should be discussed with the patient before registration in the trial.

8.2 Exclusion Criteria

1. History of severe infusion reactions to monoclonal antibodies cetuximab or panitumumab;
2. Symptomatic or untreated leptomeningeal disease and symptomatic brain metastasis;
3. Clinically significant cardiac disease including:
    a. congestive heart failure requiring treatment (NYHA grade ≥ 2), Left ventricular ejection fraction (LVEF) < 45% as determined by Multigated acquisition (MUGA) scan or echocardiogram;
    b. history or presence of clinically significant ventricular arrhythmias or atrial fibrillation;
    c. clinically significant resting bradycardia;
    d. unstable angina pectoris ≤ 3 months prior to starting study drug;
    e. acute myocardial infarction ≤ 3 months prior to starting study drug;
    f. QTcF > 480 msec;
4. History of thromboembolic or cerebrovascular events within the last 6 months, including transient ischemic attack, cerebrovascular accident, deep vein thrombosis, or pulmonary embolism;
5. Patients with interstitial pneumonitis or pulmonary fibrosis;
6. Abnormal organ or bone marrow functions defined as:
    a. Absolute neutrophil count < 1.5 x 10/L;
    b. hemoglobin < 9 g/dL;

c. alkaline phosphatase > 2.5 x upper normal limit (ULN), if liver metastases > 5 x ULN;
d. aspartate aminotransferase (AST)/ alanine aminotransferase (ALT) > 2.5 x ULN, if liver metastases > 5 x ULN;
e. bilirubin > 1.5 x ULN, if liver metastases > 2 x ULN;
f. serum creatinine > 1.5 x ULN and/or creatinine clearance ≤ 50 mL/min calculated according to Cockroft-Gault;
g. Patients with platelet count <100 x 10^9/L

7. Previous or concurrent second malignancy. Exceptions: adequately treated basal cell or squamous cell skin cancer; in situ carcinoma of the cervix, treated curatively and without evidence of recurrence for at least 3 years prior to study entry; or other solid tumor treated curatively and without evidence of recurrence for at least 3 years prior to study entry.
8. Patients with positive serology for HIV, HBV, HCV.
9. Patients with a history of severe or life threatening hypersensitivity to the active substance or to any of the excipients.

8.3 Patients substitution criteria

8.3.1 Severe Infusion reaction (Trial Phase only)

Patient experiencing **severe infusion reaction to panitumumab** requiring permanent drug discontinuation will be substituted.

9 TREATMENT (TRIAL PHASE ONLY)

9.1 Description of investigational product

Panitumumab is a recombinant, fully human IgG2 monoclonal antibody that binds with high affinity and specificity to the ligand-binding domain of human EGFR and inhibits receptor autophosphorylation induced by all known EGFR ligands. Binding of panitumumab to EGFR results

in internalization of the receptor, inhibition of cell growth, induction of apoptosis and decreased interleukin 8 and vascular endothelial growth factor production.

Panitumumab exhibits clinical activity as monotherapy or in combination with chemotherapy in wild type RAS mCRC. In Italy panitumumab is indicated for the treatment of adult patients with exon 2,3,4 K and N RAS wild type mCRC in first-line in combination with FOLFOX or FOLFIRI, in second-line in combination with FOLFIRI for patients who have received first-line fluoropyrimidine-based chemotherapy (excluding irinotecan) and as monotherapy after failure of fluoropyrimidine-, oxaliplatin-, and irinotecan-containing chemotherapy regimens.

The most common AE of panitumumab is skin toxicity occurring in 90% of patients (grade 3 NCI-CTC in 25%; grade 4 NCI-CTC < 1%) including rash (45%), dermatitis acneiform (39%), pruritus (35%), erythema (30%) and dry skin (22%); AEs present in ≥ 20% of patients are: gastrointestinal disorders [diarrhea (50%), nausea (41%), vomiting (27%), constipation (23%) and abdominal pain (23%)]; general disorders [fatigue (37%), pyrexia (20%)]; anorexia (27%); infections and infestations [paronychia (20%)]. Panitumumab is mainly distributed into the vascular space and exhibits nonlinear pharmacokinetics that are consistent with target-mediated drug disposition, involving saturable binding to EGFR and subsequent internalization and degradation inside the cells. Following the recommended dose regimen (6 mg/kg given once every 2 weeks as a 1-hour infusion), panitumumab concentrations reach steady-state levels by the third infusion with mean (± Standard Deviation [SD]) peak and trough concentrations of 213 ± 59 and 39 ± 14 mcg/ml, respectively. The mean (± SD) AUC0-tau and CL are 1306 ± 374 mcg•day/ml and 4.9 ± 1.4 ml/kg/day, respectively. The elimination half-life is approximately 7.5 days (range: 3.6 to 10.9 days). Panitumumab is also cleared in a linear fashion by the reticuloendothelial system, similarly to other endogenous immunoglobulins. A population pharmacokinetic analysis was performed to explore the potential effects of selected covariates on panitumumab pharmacokinetics. Results suggest that age (21-88), gender, race, hepatic function, renal function, chemotherapeutic agents, and EGFR membrane staining intensity (1+, 2+, 3+) in tumour cells had no apparent impact on the pharmacokinetics of panitumumab.

Panitumumab is supplied as 20 mg/ml concentrate for solution for infusion. It must be stored in a refrigerator (2°C – 8°C), protected from light.

The recommended dose of panitumumab is 6 mg/kg of body weight given once every two weeks. Prior to infusion, panitumumab should be diluted in sodium chloride 9 mg/ml (0.9%) solution for injection to a final concentration not to exceed 10 mg/ml.

Panitumumab must be administered as an intravenous infusion via an infusion pump, using a low protein binding 0.2 or 0.22 micrometer in-line filter, through a peripheral line or indwelling catheter. The recommended infusion time is approximately 60 minutes. If the first infusion is tolerated, then subsequent infusions may be administered over 30 to 60 minutes. Doses higher than 1000 mg should be infused over approximately 90 minutes.

9.2 Pregnancy and contraception

ICH M3 guidance requires precautions to be taken to minimize risk to fetus or embryo when including women of childbearing potential in clinical studies.

These precautions include the use of highly effective contraceptive measures, excluding pregnancy at baseline (serum test), continued pregnancy monitoring, and continued pregnancy testing for up to 7 months following last dose of study drug (follow-up period based on PK considerations).

Women of childbearing potential (who have not undergone surgical sterilization with a hysterectomy and/or bilateral oophorectomy) and men with partners of childbearing potential must agree to use a highly effective nonhormonal form of contraception or two effective forms of non-hormonal contraception by the patient and/or partner.

Methods that can achieve a failure rate of less than 1% per year when used consistently and correctly are considered as highly effective birth control methods. Such methods include:

- combined (estrogen and progestogen containing) hormonal contraception associated with inhibition of ovulation
  - oral
  - intravaginal
  - transdermal
- progestogen-only hormonal contraception associated with inhibition of ovulation:
  - oral
  - injectable
  - implantable
- intrauterine device (IUD)
- intrauterine hormone-releasing system (IUS)
- bilateral tubal occlusion

- vasectomised partner[1]
- sexual abstinence[2]

9.3 Treatment Plan

9.3.1 Drug Administration

Treatment will be administered on an outpatient basis. No investigational or commercial agents or therapies other than those described below may be administered with the intent to treat the patient's malignancy.

**Regimen Description**				
Agent	Precautions	Dose	Route	Schedule
Panitumumab	Avoid exposure to sunlight	6 mg/kg in 100 cc 0.9% NaCl solution	IV over 1 hour	Day 1 every 2 weeks

The panitumumab dose will be calculated based on the subject's actual body weight at baseline and re-calculated at subsequent doses per institutional guidelines. At a minimum, the dose will be re-calculated if the actual body weight changes by at least 10%. Panitumumab will be administered IV by an infusion pump through a peripheral line or indwelling catheter using a non-pyrogenic, low protein binding filter with a 0.2 or 0.22-micron in-line filter infusion set-up over 1 hour ± 15 minutes by a trained healthcare professional.

If the first infusion of panitumumab is well tolerated (ie without any serious infusion-related reactions) all subsequent infusions may be administered over 30 ± 10 minutes. In the event a subject's actual weight requires greater than 150 mL volume infusion, panitumumab will be administered over 60 to

1 *Vasectomised partner is a highly effective birth control method provided that partner is the sole sexual partner of the WOCBP trial participant and that the vasectomised partner has received medical assessment of the surgical success.*

2 *Sexual abstinence is considered a highly effective method only if defined as refraining from heterosexual intercourse during the entire period of risk associated with the study treatments. The reliability of sexual abstinence needs to be evaluated in relation to the duration of the clinical trial and the preferred and usual lifestyle of the subject.*

90 minutes ± 15 minutes, as tolerated. Doses higher than 1000 mg should be diluted to 150 mL in 0.9% sodium chloride solution, USP (saline solution) and infused over 60 to 90 ± 15 minutes.

9.3.2 Premedication and patient monitoring during infusion

Pre-medications are not required for panitumumab but medications such as corticosteroids and anti-histamines may be administered to treat an existing infusion reaction or as pre-medication for a subject who has previously experienced an infusion reaction at the discretion of the investigator.
Patients will be monitored for any signs of AEs after their first dose of panitumumab for at least 60 minutes.

9.3.3 Duration of Therapy

Treatment may continue until one of the following criteria applies:

- Disease progression;
- Intercurrent illness that prevents further administration of treatment/death;
- Unacceptable adverse event(s);
- Patient decides to withdraw from the study, or
- General or specific changes in the patient's condition render the patient unacceptable for further treatment in the judgment of the investigator.

9.4 Concomitant Therapy

Throughout the study, investigators may prescribe any concomitant medications or treatments deemed necessary to provide adequate supportive care.
Anticancer therapy (chemotherapy, biologic or radiation therapy, palliative radiotherapy covering > 25% of the bone marrow reserve, and surgery) must not be given to patients during the study treatment.
Radiotherapy for palliation in non-target lesions with antalgic aim is allowed.
Recombinant human granulocyte colony stimulating factor (G-CSF) or erythropoiesis stimulating agent use according to the current approved label or institutional guidelines is permitted.

9.5 Dose Adjustments

Doses will be reduced for hematological and other adverse events. Dose adjustments are to be made according to the greatest degree of toxicity. Adverse events will be graded using the NCI Common Terminology Criteria for Adverse Events (CTCAE) Version 4.03 (download from the CTEP web site http://ctep.cancer.gov/protocolDevelopment/electronic_applications/ctc.htm).
The major adverse effects of panitumumab which limit dose is dermatologic reactions and soft tissue toxicity. Are also common anemia, gastrointestinal disorders, hypokalemia, hypomagnesaemia, anorexia, conjunctivitis, dyspnea and cough.
The guidelines that follow outline dose adjustments for several of these toxic effects. If a patient experiences several adverse events and there are conflicting recommendations, please use the recommended dose adjustment that reduces the dose to the lowest level.

9.5.1 Panitumumab Dose Reductions

Dose Level	Percentage (%)	Panitumumab
Starting Dose	100	6 mg/kg Q2W
-1	80	4.8 mg/kg Q2W
-2	60	3.6 mg/kg Q2W

9.5.2 Criteria for Withholding a Dose of Panitumumab

Panitumumab will be withheld if any of the following related toxicities occur:

- Skin- or nail-related toxicities:
  - Any grade ≥ 3 toxicity;
  - Any skin- or nail-related SAE;
- Non-skin- or non-nail-related toxicities:
  - Any grade 3 or 4 toxicity with the following exceptions:
    - ♦ Panitumumab will only be withheld for grade ≥ 3 hypomagnesemia and/or hypocalcemia that persists despite maximal (ie, IV replacement) magnesium and/or calcium replacement;
    - ♦ Panitumumab will only be withheld for grade ≥ 3 nausea, diarrhea, or vomiting that persists despite optimal supportive care;

♦ Panitumumab will only be withheld for grade ≥ 3 anemia or grade 4 thrombocytopenia that can't be managed by transfusion(s).

9.5.3 Criteria for Re-treatment with Panitumumab

<u>Skin- or nail-related toxicities</u>:

Panitumumab administration may recommence once the reason for withholding the dose of panitumumab has resolved:

- Systemic steroids are no longer required, or
- The skin- or nail-related toxicity is no longer intolerable to the subject, or
- IV antibiotic or IV antifungal treatment is no longer required, or
- The AE has improved to grade ≤ 2 or returned to baseline, and is no longer considered serious.

<u>Non-skin- or nail-related toxicities</u>:

Panitumumab administration may recommence at the next planned visit once the AE has improved to ≤ grade 1 or returned to baseline, unless otherwise indicated (see Section 9.4.5 and 9.4.6).

9.5.4 Dose Modification Schedule for Panitumumab

Subjects who have had their dose of panitumumab withheld for one or more reasons listed in Section 9.4.2 and then meet the criteria for re-treatment as listed in section 9.4.3 will recommence panitumumab at the next planned visit according to the following schedule:

**Occurrence of skin- or nail-related toxicities: ≥ grade 3**	**Administration of panitumumab**	**Outcome**	**Dose regulation**
Initial occurrence	Withhold 1 or 2 doses	Improved (≤ grade2)	Continuing infusion at 100% of original dose
		Not recovered	Discontinue
At the second occurrence	Withhold 1 or 2 doses	Improved (≤ grade2)	Continuing infusion at 80% of original dose
		Not recovered	Discontinue
At the third occurrence	Withhold 1 or 2 doses	Improved (≤ grade2)	Continuing infusion at 60% of original dose
		Not recovered	Discontinue
At the fourth occurrence	Discontinue	-	-

**Occurrence of Non-skin- or Non-nail-related toxicities:** **≥ grade 3 (for the exceptions see above)**	**Administration of panitumumab**	**Outcome**	**Dose regulation**
Initial occurrence	Withhold 1 or 2 doses	Improved (≤ grade1)	Continuing infusion at 100% of original dose
		Not recovered	Discontinue
At the second occurrence	Withhold 1 or 2 doses	Improved (≤ grade1)	Continuing infusion at 80% of original dose
		Not recovered	Discontinue
At the third occurrence	Withhold 1 or 2 doses	Improved (≤ grade1)	Continuing infusion at 60% of original dose
		Not recovered	Discontinue
At the fourth occurrence	Discontinue	-	-

It is recommended that panitumumab doses will be escalated in subjects whose toxicity resolves to the degree that meets the criteria for re-treatment with panitumumab. Dose escalations are recommended but not required.

Dose escalations should occur in the following manner:

- Subjects treated at the 80% dose level whose toxicity does not recur should receive the 100% dose level at the next dose unless a previous attempt to re-escalate to the 100% dose level was not tolerated (re-initiation of the 80% dose is allowed as an alternative to dose escalation).
- Subjects treated at the 60% dose level whose toxicity does not recur should receive the 80% dose at the next dose unless a previous attempt to re-escalate to the 80% dose level was not tolerated (re-initiation of the 60% dose is allowed as an alternative to dose escalation).

9.5.5 Delayed or Missed Doses

If panitumumab is not given within ± 3 days of the planned visit, then the dose will be considered missed. The next dose should be given at the next planned visit. Missed doses will not be made up.

9.5.6 Discontinuation

Subjects for whom a delay of panitumumab administration is due to a related toxicity and is of > 6 weeks (from the planned administration date, ie, ≥ 3 consecutively missed doses) will be considered intolerant and be permanently discontinued.

9.5.7 Management of dermatologic toxicity and recommended concomitant treatment

Pre-emptive treatment of anticipated skin toxicity is strongly recommended since it can halve the incidence rate of grade 2 or higher skin toxicity from approximately 60% to 30%, including a reduction from 21% to 6% of grade 3 toxicity.
Subjects treated with panitumumab should receive the pre-emptive regimen 24 hours before study Day 1 and continue for at least 6 weeks, preferably for the duration of treatment. Proactive skin treatment includes:

- skin moisturiser and sun screen (SPF > 15 UVA and UVB), to be applied to face, hands, feet, neck, back and chest <u>every morning during treatment</u>
- topical steroid cream (not stronger than 1% hydrocortisone) to be applied face, hands, feet, neck, back and chest every night during treatment.

Oral antibiotic (e.g. doxycycline) may be useful in the management of dermatologic reactions.
Subjects who experience dermatological toxicities that meet the criteria defined in Section 9.4.2 should have panitumumab dose withheld as described in section 9.4.4.

10 CLINICAL EVALUATION, LABORATORY TESTS, FOLLOW-UP

10.1 Before Treatment Start

Given the progressively restrictive selection only a fraction of the patients undergoing the molecular screening will proceed to the Trial Phase.
They following information must be present:

- Dated IEC/IRB approved informed consent form;
- An imaging documented progression at last treatment with anti-EGFR
- An imaging documented progression at last line therapy not including anti-EGFR mAb;

- Documented absence of RAS extended, RAF and EGFR ectodomain mutations clones at liquid biopsy performed no longer than 4 weeks before trial entry;
- Documentation of demographics, relevant medical history, concomitant medication;
- Detailed information on prior cancer treatment (previous front-line chemotherapy and anti-EGFR mAb treatment, start date, stop date, best response and best response date, date of PD, reason for stopping; previous line chemotherapies, start date, stop date, best response and best response date, date of PD, reason for stopping.
- Tumor histology, location and extent of disease, tumor tissue RAS and BRAF genotyping assayed with the Panel of genes reported in Appendix B, or RAS and BRAF status at initial diagnosis and the FFPE block or at least 10 unstained slides of the diagnostic paraffin tumor;
- Safety laboratory (Blood count with WBC count and differential, sodium, potassium, chloride, urea, magnesium, creatinine, albumin, total protein, total bilirubin, alkaline phosphatase, ALT, AST, calcium, phosphorous, uric acid, INR, aPTT, pregnancy test [if applicable]);

- Baseline Safety (symptoms and persisting toxicities from prior therapy to check compliance with inclusion criteria);
- Serology testing for :
    - Hepatitis B virus: HBsAg, antibodies against HBsAg, anti-hepatitis B core antibody (anti-HBcAb);
    - Hepatiatis C virus: HCV antibody (anti-HCV);.
    - HIV testing: patients will be tested for HIV prior to the inclusion into the study
- Physical examination including assessment of vital signs (blood pressure, heart rate, body temperature), body weight, height, ethnicity, dermatological examination (CTCAE v4 scaling);
- ECOG performance status;
- 12-lead ECG;
- Tumor assessment. Computed tomography (CT) or magnetic resonance imaging (MRI) of the chest and abdomen must have been performed no longer than 28 days prior to first treatment. Imaging must be performed with contrast and baseline status of the tumor disease

using RECIST v1.1. (note it could coincide with the radioimaging of the second line therapy progression). Additional imaging (such as bone scan) must be performed when other areas of disease are suspected. In addition, all imaging evaluation performed during the pre-trial screening phase must be available.

Refer to the Summary table (Section 4.2) for an outline of procedures required at each visit.

10.2 During Treatment

The following assessments are to be performed according to the following schedule during the treatment phase in all subjects:

Every two weeks:

- Physical examination (normal/abnormal, weight);
- Vital signs (body temperature, heart rate, and blood);
- ECOG performance status;
- Laboratory tests local (hematology, coagulation, and serum chemistry);
- AEs (signs and symptoms);
- Concomitant medications (all concomitant medications including prophylactic use of antibiotics);
- Blood sampling for molecular analysis.

Every 8 weeks:

Tumor assessment. CT or MRI of the chest and abdomen. Additional imaging must be performed when other areas of disease are known or suspected. Tumor burden evaluation per RECIST v1.1. (+/- 3 days).

Refer to the Schedule of Events (See Section 4.2) for an outline of the frequency of the required procedures.

10.3 After the End of Treatment (Follow-Up)

Patients who discontinue study treatment should be scheduled for a safety follow-up visit within 28 days after the last dose of study or after the decision to discontinue study treatment.

This visit includes:

- Physical examination, weight, vital signs;
- ECOG PS;
- ECG;
- Laboratory tests local (hematology, coagulation, and serum chemistry);
- Tumor assessment. CT or MRI of the chest and abdomen. Additional imaging must be performed when other areas of disease are known or suspected. Tumor burden evaluation per RECIST v1.1;
- AEs;
- Concomitant medications/procedures;
- Blood sample for NGS molecular analysis;

An End of Treatment eCRF page should be completed, giving the date and reason for stopping the study treatment.

Patients who discontinue study treatment for any reason prior to disease progression (except informed consent withdrawn) should return for disease follow-up and blood sampling for the plasma longitudinal ct-DNA study every 8 weeks until progression, with a last sampling on day 28 post progression, at which time they will be considered withdrawn from the study.

If the subject ends treatment for reasons other than documented disease progression, then CT or MRI scans will be performed at end-of treatment visit and then every 8 weeks (+/- 3 days) until radiographically-confirmed disease progression, start of new cancer treatment, death, withdrawal of consent or at the end of the study, whichever is earlier. Assessments will be made by the Investigator using RECIST v1.1.

11 EFFICACY ASSESSMENT

11.1 Definitions

All eligible patients will be included in the response rate calculation (primary end-point). The subsets that will be assigned a response category (CR, PR, SD or PD; see definitions below) are all patients who have received at least one treatment and have their disease re-evaluated. Patients on will have their response classified according to the definitions set out below.

11.2 Tumour assessments

Tumour assessments will be performed at each center by local radiologists according to RECIST version 1.1. Screening/baseline imaging assessments may be performed up to 28 days prior of treatment start on-study tumor assessments every 8 weeks with a ± 3 day window of variance. In case of discontinuation of treatment for any reason other than disease progression, patients will still be re-evaluated every 8 weeks until disease progression or start of a new treatment or death or patients refusal, whichever comes first. At trial end, all in-Trial tumor assessments, and the radioimaging data at each of the Trial checkpoints (BML and RML) will be reviewed centrally by two radiologists who will read the CT/MRI scans blinded using the mintLesion™ software to collect, store, and guide the revision of the imaging results. The imaging review protocol and tumour assessment re-conciliation report will be included in the final study report and or in the publication of the study.
The complete criteria are included in the published RECIST document also available at http://www.eortc.be/RECIST.

11.2.1 Measurability of Tumour Lesions at Baseline

11.2.1.1 Definitions

- **Measurable disease** - the presence of at least one measurable lesion. If the measurable disease is restricted to a solitary lesion, its neoplastic nature should be confirmed by cytology/histology.
- **Measurable lesions** - *tumour lesions* that can be accurately measured in at least one dimension (longest diameter to be recorded) as ≥ 20 mm with chest x-ray, and as ≥ 10 mm with CT scan or clinical examination [using calipers]. Bone lesions are considered measurable only if assessed by CT scan and have an identifiable soft tissue component that meets these requirements (soft tissue component ≥ 10 mm by CT scan). *Malignant lymph nodes* must be ≥ 15 mm in the short axis to be considered measurable; only the short axis will be measured and followed. All tumour measurements must be recorded in millimeters (or decimal fractions of centimeters) by use of a ruler or calipers. Tumour lesions situated in a previously irradiated area, or in an area subjected to other loco-regional therapy, are usually not considered measurable unless there has been demonstrated progression in the lesion. Provide detail on the conditions under which such lesions would be considered measurable.

- **Non-measurable lesions** - All other lesions (or sites of disease), including small lesions are considered non-measurable disease. Bone lesions without a measurable soft tissue component, leptomeningeal disease, ascites, pleural/pericardial effusions, lymphangitis cutis/pulmonis, inflammatory breast disease, lymphangitic involvement of lung or skin and abdominal masses followed by clinical examination are all non-measurable. Nodes that have a short axis <10 mm at baseline are considered non-pathological and should not be recorded or followed.
- **Target Lesions.** When more than one measurable tumour lesion or malignant lymph node is present at baseline all lesions up to *a maximum of 5 lesions total* (and a maximum of *2 lesions per organ*) representative of all involved organs should be identified as target lesions and will be recorded and measured at baseline. Target lesions should be selected on the basis of their size (lesions with the longest diameter), be representative of all involved organs, but in addition should be those that lend themselves to *reproducible repeated measurements*. Note that pathological nodes must meet the criterion of a short axis of ≥ 15 mm by CT scan and only the *short* axis of these nodes will contribute to the baseline sum. At baseline, the sum of the target lesions (longest diameter of tumour lesions plus short axis of lymph nodes: overall maximum of 5) is to be calculated and recorded.
- **Non-target Lesions**. All non-measurable lesions (or sites of disease) including pathological nodes (those with short axis ≥ 10 mm but < 15 mm), plus any measurable lesions over and above those listed as target lesions are considered *non-target lesions*. Measurements are not required but these lesions should be noted at baseline and should be followed as “present” or “absent”.

All baseline evaluations should be performed as closely as possible to the beginning of treatment and never more than 4 weeks before the beginning of the treatment.

11.2.1.2 Methods of Measurements

The same method of assessment and the same technique should be used to characterize each identified and reported lesion at baseline and during follow-up. Assessments should be identified on a calendar schedule and should not be affected by delays in therapy. While on study, all target lesions recorded at baseline should have their actual measurements recorded on the CRF at each subsequent evaluation, even when very small (e.g. 2 mm). If it is the opinion of the radiologist that

the lesion has likely disappeared, the measurement should be recorded as 0 mm. If the lesion is believed to be present and is faintly seen but too small to measure, a default value of 5 mm should be assigned. For lesions which fragment/split add together the longest diameters of the fragmented portions; for lesions which coalesce, measure the maximal longest diameter for the "merged lesion".

- Clinical Lesions. Clinical lesions will only be considered measurable when they are superficial and ≥ 10 mm as assessed using calipers (e.g. skin nodules). For the case of skin lesions, documentation by color photography including a ruler to estimate the size of the lesion is recommended. If feasible, imaging is preferred.
- CT, MRI. CT is the best currently available and reproducible method to measure lesions selected for response assessment. This guideline has defined measurability of lesions on CT scan based on the assumption that CT slice thickness is 5 mm or less. When CT scans have slice thickness greater than 5 mm, the minimum size for a measurable lesion should be twice the slice thickness. MRI is also acceptable in for abdomen imaging.
- All measurements should be taken and recorded in metric notation using a ruler or calipers.

11.2.2 Tumour Response Evaluation

The activity of panitumumab will be assessed by the investigator according to the RECIST v1.1 (refer to the table below).

Complete Response (CR): disappearance of all *target* and *non-target* lesions and normalization of tumour markers. Pathological lymph nodes must have short axis measures < 10 mm (Note: continue to record the measurement even if < 10 mm and considered CR). Tumour markers must have normalized. Residual lesions (other than nodes < 10 mm) thought to be non-malignant should be further investigated (by cytology or PET scans) before CR can be accepted.

Partial Response (PR): at least a 30% decrease in the diameter of the target lesion or in the sum of measures (longest diameter for tumour lesions and short axis measure for nodes) of target lesions, taking as reference the baseline measurement or sum of diameters. Non target lesions must be non-PD.

Objective Response Rate: the sum of partial responses plus complete responses.

Stable Disease (SD): Neither sufficient shrinkage to qualify for PR nor sufficient increase to qualify for PD taking as reference the smallest sum of diameters on study.

Progressive Disease (PD): at least a 20% increase in the sum of diameters of measured lesions taking as references the smallest sum of diameters recorded on study (including baseline) AND an absolute increase of ≥ 5 mm. Appearance of new lesions will also constitute PD (including lesions in previously unassessed areas). In exceptional circumstances, unequivocal progression of non-target disease may be accepted as evidence of disease progression, where the overall tumour burden has increased sufficiently to merit discontinuation of treatment, for example where the tumour burden appears to have increased by at least 73% in volume (which is the increase in volume when all dimensions of a single lesion increase by 20%). Modest increases in the size of one or more non-target lesions are NOT considered unequivocal progression. If the evidence of PD is equivocal (target or non-target), treatment may continue until the next assessment, but on further documentation, the earlier date must be used.

Integration of target, non-target and new lesions into response assessment.

Target Lesions	Non-Target Lesions	New Lesions	Overall Response
Patients with Target lesions ± non target lesions			
CR	CR	No	CR
CR	Non-CR/Non-PD	No	PR
CR	Not all evaluated	No	PR
PR	Non-PD/ not all evaluated	No	PR
SD	Non-PD/ not all evaluated	No	SD
Not all evaluated	Non-PD	No	NE
PD	Any	Any	PD
Any	PD	Any	PD
Any	Any	Yes	PD
Patients with Non target lesions ONLY			
No Target	CR	No	CR
No Target	Non-CR/non-PD	No	Non-CR/ non-PD
No Target	Not all evaluated	No	NE
No Target	Unequivocal PD	Any	PD

No Target	Any	Yes	PD
Note: Patients with a global deterioration of health status requiring discontinuation of treatment without objective evidence of disease progression [or evidence of unequivocal disease progression] at that time should be reported as "*symptomatic deterioration*". This is a reason for stopping therapy, but is NOT objective PD. Every effort should be made to document the objective progression even after discontinuation of treatment.			

11.2.2.1 Frequency of Tumour Re-Evaluation

Tumor evaluation will be performed at baseline and every 8 weeks during the study treatment until disease progression. However in order to fulfil RECIST, the FIRST occurrence of response MUST be reconfirmed not earlier than 4 and not later than 5 weeks later.

Screening/baseline imaging assessments may be performed within 28 days of treatment start, on-study tumor assessments have a +/- 3 days window.

In case of discontinuation of treatment for any reason other than disease progression, patients will still be re-evaluated every 8 weeks.

11.2.3 Date of Progression

Date of progression is defined as the first day when RECIST (version 1.1) PD is observed.

11.2.4 Reporting of Tumour Response

All patients included in the study must be assessed for response to treatment, even if there is a major protocol treatment deviation or if they are ineligible, or not followed/re-evaluated. Each patient will be assigned one of the following categories: complete response, partial response, stable disease, progressive disease, early death from malignant disease, early death from toxicity, early death from other cause or unknown (not assessable, insufficient data).

Early death is defined as any death occurring before the first per protocol time point of tumour re-evaluation. The responsible investigator will decide if the cause of death is malignant disease, toxicity or other cause.

Patients for whom response is not confirmed will be classified as "unknown", unless they meet the criteria for stable disease (or the criteria for partial response in case of an unconfirmed complete

response). Patients' response will also be classified as "unknown" if insufficient data were collected to allow evaluation per these criteria.

11.3 Progression Free Survival

PFS is defined as the time from start of treatment to the first date of documented progression or death, whichever occurs first. Patients who are progression-free at the time of analysis will be censored at the date of last disease assessment.

11.4 Overall Survival

OS is defined as the time from start of treatment until the date of death due to any cause, patients still alive at the time of analysis will be censored at the date of the last visit.

12 SAFETY ASSESSMENT

All patients that received at least one dose of panitumumab will be evaluable for safety and toxicity analysis. Toxicity will be assessed using the Common Toxicity Criteria for Adverse Events version 4.03 (CTCAE). All adverse events (see section 11), up to 14 days after the last administration of study treatment, will be recorded on the case report forms; the investigator will decide if those events are drug related (not related, not likely, possibly, probably, certainly) and this decision will be recorded on the forms for all adverse events. Serious adverse events are defined by the Good Clinical Practice Guideline. Serious adverse events must be immediately reported according to the procedure detailed below.

12.1 Definitions for Adverse Event

Adverse Event (AE) is defined as any untoward medical occurrence or experience in a patient or clinical investigation subject which occurs following the administration of the trial medication regardless of the dose or causal relationship. This can include any unfavorable and unintended signs (such as rash or enlarged liver), or symptoms (such as nausea or chest pain), an abnormal laboratory finding (including blood tests, x-rays or scans) or a disease temporarily associated with the use of the protocol treatment *(ICH-GCP).*

An **Adverse Drug Reaction (ADR)** is defined as any response to a medical product, that is noxious and/or unexpected, related to any dose *(ICH-GCP).*

Response to a medicinal product (used in the above definition) means that a causal relationship between the medicinal product and the adverse event is at least a reasonable possibility, i.e. the relationship cannot be ruled out.

An **Unexpected Adverse Drug Reaction** is any adverse reaction for which the nature or severity is not consistent with the applicable product information, like the Investigators' Brochure *(ICH-GCP).*

A **Serious Adverse Event (SAE)** is defined as any undesirable experience occurring to a patient, whether or not considered related to the protocol treatment. A Serious Adverse Event (SAE) which is considered related to the protocol treatment is defined as a **Serious Adverse Drug Reaction (SADR)**.

Adverse events and adverse drug reactions which are considered as **serious** are those which result in:

- death;
- a life threatening event (i.e. the patient was at immediate risk of death at the time the reaction was observed);
- hospitalization or prolongation of hospitalization;
- persistent or significant disability/incapacity;
- a congenital anomaly/birth defect;
- any other medically important condition (i.e. important adverse reactions that are not immediately life threatening or do not result in death or hospitalization but may jeopardize the patient or may require intervention to prevent one of the other outcomes listed above).

12.2 Reporting procedures for Adverse Event

Toxicity will be scored using NCI Clinical Trials Criteria for Adverse Events (CTCAE) Version 4.03. AEs will be collected from the time the first dose of study medication is administered until 14 days following discontinuation of study medication regardless of initiation of a new cancer therapy or transfer to hospice. SAEs will be collected over the same time period as stated above for AEs. In addition, any SAE assessed as related to study participation, study medication or concomitant medication must be recorded from the time a subject consents to participate in the study up to and including any follow-up contact.

After discontinuation of study medication, the investigator will monitor all AEs/SAEs that are ongoing until resolution or stabilization of the event or until the subject is lost to follow-up.

Disease progression is not to be reported as an AE since time to progression (TTP) after Panitumumab Rechallenge is an endpoint of the study (See Section 6.2).

A death on study requires reporting regardless of causality and attribution to treatment or other cause must be provided. Death due to disease progression is not to be reported as an AE. Deaths that occur beyond 30 days after the end of study drug administration/initiation of an alternate therapy, do not qualify as SAEs.

Each adverse event is to be classified by the investigator as SERIOUS or NON-SERIOUS. This classification of the seriousness of the event determines the reporting procedures to be followed. If a serious adverse event occurs, the Istituto di Candiolo Pharmacovigilance (Fax Number: + 011 993 3261r mailbox: farmacia@ircc.it) is to be notified, using the SAE report form, within 24 hours of awareness of the event by the investigator. If the initial report is incomplete or the event is still ongoing at the time of reporting or if new significant information becomes available, this report is to be followed by submission of follow-up information within 5 calendar days after the initial notification. Reporting requirements for adverse events are summarized in the following table.

12.3 Reporting requirements for adverse event

Gravity	Reporting Time	Type of Report
**SERIOUS**	Within 24 hours from awareness by the investigator	Initial report on SAE report form + case report form
	Within 5 calendar days from initial report	Follow-up/Final report on SAE report form
**NON SERIOUS**	Per case report form submission procedure	Case report form

If for any reason the SAE form transmission is not possible, Istituto di Candiolo Pharmacovigilance should be informed by phone 011 993 3261) of the occurrence of the event. In this exceptional case, Istituto di Candiolo Pharmacovigilance will complete a SAE form with information received, which will be sent to the investigator for confirmation, and in the meanwhile Pharmacovigilance procedures will be initiated.

Istituto di Candiolo Pharmacovigilance will submit to the concerned drug company (Amgen) all SAEs occurring in this trial, regardless of whether the investigator suspects causality with the study treatment.

Serious adverse events should also be reported on the adverse event case report form. The form to be used for serious adverse event expedited reporting is not the same as the adverse event case report form, but where the same data are collected, the forms must be completed in a consistent manner. For example, the same adverse event term should be used on both forms.

Istituto di Candiolo assesses each SAE reported by the study Investigators to identify any suspected unexpected serious adverse reactions (SUSAR), i.e. serious adverse events considered at least possible associated to the study treatment by the Investigator and not listed in the IMP reference document(s) and provide this information to the sponsor for the final evaluation.

If a SAE is assessed as a possible SUSAR, the sponsor, through Istituto di Candiolo Pharmacovigilance, may urgently require further information to the Investigator. Istituto di Candiolo Pharmacovigilance will issue a SUSAR (Suspect Unexpected Serious Adverse Reaction) notification whenever appropriate, and submit it to all concerned recipients according to current law and following sponsor's indication.

Follow-up information is to be reported on a new serious adverse event form and transmitted to the same fax number as the initial report. A follow-up report is to be filled in, not only to complete the information provided on the initial report but also to modify any incorrect data.

The SAE fax delivery confirmation sheets must be retained at the study sites.

At the end of the study all original SAE report forms are to be collected by the CRO personnel and delivered to Istituto di Candiolo for archiving in the TMF, while the corresponding copies must be retained in the Investigator File.

The Sponsor shall provide Amgen with a copy of any SAE report received by the Investigators for patients treated with panitumumab. The Sponsor will send the SAE forms to Amgen Safety to the following fax number: 800916570 (in case of any issues with the fax number or for any other question the following e-mail address: *eu-it-farmacovigilanza@amgen.com* can be used).

All SAEs must be promptly forwarded to Amgen (in accordance with the local law requirements); a copy of SUSARs must be sent to Amgen at time of regulatory submission. Pregnancy and Lactation reports must be sent within 10 days of Sponsor awareness.

The Sponsor will also provide Amgen with a Development Safety Update Report (DSUR) and with a copy of any other communication or aggregate report, containing safety data generated during the course of the study, sent to the Regulatory Authorities by the Sponsor. Furthermore, the Sponsor will provide Amgen with a line listing/report of all SAEs occurred in subjects exposed to Amgen Product on a periodic basis (but no less frequently than every 6 months) in order to fulfill the reconciliation process.
The final study report should be sent to Amgen no later than 1 calendar year of study completion.

12.4 Reporting requirements for complaints related to panitumumab

All product complaints relating to Panitumumab will be reported directly to Amgen at the time of discovering the complaint. Such complaints include:

- packaging (eg, broken container or cracked container)
- function (eg, subject or healthcare provider cannot appropriately use the product despite training, [eg, due to malfunction of the auto-injector (AI)/Pen or Personal Injector])
- labeling (eg, missing labels, illegible labels, incorrect labels, and/or suspect labels)
- change in IP appearance (eg, color change or visible presence of foreign material)
- unexpected quantity or volume (eg, number of tablets or amount of fluid in bottle/vial)
- evidence of tampering or stolen material'.

Reports must be made to AMGEN using the “Product Complaint Form”.

12.5 Recording Adverse Events in the Case Report Forms

AEs can be assessed directly by the Investigator during a clinical visit or based on laboratory/Instrumental examinations or can be referred by the patient.

12.5.1 Pre-existing Conditions

A pre-existing condition (i.e., a disorder starting before the adverse event reporting period) should not be reported as an adverse event unless the condition worsens during the adverse event reporting period.

12.5.2 Procedures

Diagnostic and therapeutic procedures, such as surgery, should not be reported as adverse events, while the medical condition for which the procedure was performed should be reported if it meets the definition of an adverse event. For example, an appendectomy performed for an acute appendicitis occurring during the adverse event reporting period should not be reported as adverse event; while "acute appendicitis" is to be reported as adverse event. If a patient undergoes a surgical procedure that was planned prior to entry into the trial, and surgery is not performed due to a worsening of a baseline condition, this baseline condition should not be reported as an adverse event.

12.5.3 Symptoms of Targeted Disease

Tumor-related signs and symptoms will be followed at each visit. Although a measure of efficacy, these will always be reported as pre-existing conditions at baseline and during treatment only if they meet the definition of adverse event.

For all adverse events the Investigator will be asked to assess its relationship with the study treatment.

12.5.4 Causality Assessment and Grading of Adverse Event Severity

The assessment of relationship to study drug will be done according to the following causality scale based on the WHO definitions:

- Certain: A clinical event, including laboratory test abnormality, occurring in a plausible time relationship to drug administration, and which cannot be explained by concurrent disease or other drugs or chemicals. The response to withdrawal of the drug (de-challenge) should be clinically plausible. The event must be definitive pharmacologically or phenomenologically, using a satisfactory re-challenge procedure if necessary
- Probable: A clinical event, including laboratory test abnormality, with a reasonable time sequence to administration of the drug, unlikely to be attributed to concurrent disease or other drugs or chemicals, and which follows a clinically reasonable response on withdrawal (rechallenge). Rechallenge information is not required to fulfil this definition.
- Possible: A clinical event, laboratory test abnormality, with a reasonable time sequence to administration of the drug, but which could also be explained by concurrent disease or other drugs or chemicals. Information on drug withdrawal may be lacking or unclear

- Unlikely: A clinical event, laboratory test abnormality, with a temporal relationship to drug administration which makes a causal relationship improbable, and in which other drugs, chemicals or underlying disease provide plausible explanations Severity grading of adverse events and pre-existing conditions will be done according to the National Cancer Institute (NCI) Common Toxicity Criteria (CTCAE) V. 4.03.

AEs that are not defined in the NCI CTCAE should be evaluated for severity according to the following scale:

- Grade 1 = Mild – transient or mild discomfort; no limitation in activity; no medical intervention/therapy required;
- Grade 2 = Moderate – mild to moderate limitation in activity, some assistance may be needed; no or minimal medical intervention/therapy required;
- Grade 3 = Severe – marked limitation in activity, some assistance usually required; medical intervention/therapy required, hospitalization is possible;
- Grade 4 = Life threatening – extreme limitation in activity, significant assistance required; significant medical intervention/therapy required, hospitalization or hospice care probable;
- Grade 5 = Death - the event results in death.

Note the distinction between the gravity and the severity of an adverse event. Severe is a measure of intensity; thus, a severe reaction is not necessarily a serious reaction. For example, a headache may be severe in intensity but would not be classified as serious unless it meets one of the criteria for serious events listed above.

12.5.5 Pregnancy Reporting

Any pregnancy that occurs during study participation must be reported using the “exposure in utero” form. To ensure subject safety, each pregnancy must be reported to Istituto di Candiolo Pharmacovigilance within 2 weeks of learning of its occurrence. The pregnancy must be followed-up to determine outcome (including premature termination) and status of mother and child. Pregnancy complications and elective terminations for medical reasons must be reported as an AE or SAE. Spontaneous abortions must be reported as a SAE.

Any SAE occurring in association with a pregnancy brought to the investigator’s attention after the subject has completed the study and considered by the investigator as **possibly related** to study treatment, must be promptly reported to Istituto di Candiolo Pharmacovigilance.

In addition, the investigator must attempt to collect pregnancy information on any female partners of male study subjects who become pregnant while the subject is enrolled in the study. Pregnancy information must be reported to Istituto di Candiolo Pharmacovigilance as described above.

12.5.6 Overdose

Reporting if any overdose (accidental or intentional) which results in serious adverse reactions is to be handled following the SAE procedures. This includes reports related to drug intake with suicidal intentions and consequent drug overdose.

Overdose reporting even not associated with adverse reactions shall be anyhow reported immediately to Istituto di Candiolo Pharmacovigilance using the most rapid type of communication (phone, e-mail).

12.5.7 Follow-up of Unresolved Adverse Events

All adverse events should be followed at least until 28 days following the last dose of IMP. Drug-related and serious adverse events ongoing at the end of this observation period must be recorded until they are resolved or the investigator assesses them as chronic or the subject is lost to follow-up or starts a new anti-cancer treatment, whichever occurs earlier.

13 TRANSLATIONAL ASSESSMENT

Patients are liquid biopsied at two checkpoints: BML (at end of first anti-EGFR PD, optional), and RML (mandatory), and during Trial Phase on day 1 and 15 of each cycle until progression.

Specific Standard Operating Procedures will be used according to our Liquid Biopsy Guidelines (See Appendix C).

Isolated circulating free DNA is analyzed using ddPCR™(Bio-Rad) or by Next Generation Sequencing using Illumina platforms as previously described. Next generation DNA sequencing based on Illumina reagents is widely used and considered highly reliable. The NGS LB approaches we developed are based on Illumina reagents.

The collection information must be captured on the Biomarker Assessment eCRF pages and Central Lab Requisition forms.

The expanded RAS molecular status will be analyzed by plasma ddPCR to investigate how the RAS profile is affected (if any) by panitumumab rechallenge. The mutation load and the duration of mutation free plasma (if any), will be measured.

The molecular landscape of plasma ctDNA will be defined by NGS at RML and in the LB at progression of all patients participating to the Trial Phase.

14 STATISTICAL CONSIDERATIONS

14.1 Sample Size

We used the A'Hern one-stage approach to calculate the sample size. For the primary objective, we will need to enroll 27 patients in order to achieve a power of at least 85% to test the null hypothesis that the rate of response to panitumumab would be 10% or less, versus the alternative hypothesis that the response rate would be 30% or more, at a one-sided alpha level of 0.05. Six objective responses are necessary to declare the study positive.

Protocol amendment number 2 restricts, while simplifying the eligibility criteria to patients with a negative liquid biopsy for extended RAS/RAF and EGFR ectodomain mutations, while eligibility in the prior version required a reduction of at least 50% of extended RAS/RAF positive clones. The expected Panitunumab rechallenge ORR remains however the same i.e. 30%, requiring under the same alfa and beta assumption a sample size of 27 patients.

27 patients need to be recruited in the amended protocol v3.0. Patients resulting positive to the Panel B genotypization will be analyzed by the intention-to-treat (ITT) approach and will be included in the efficacy analysis population (see statistical analysis paragraph 14.2).

In the previous protocol v. 2.1 dated 30.10.2017, four patients were enrolled with the previous criteria. All these patients left the trial for progression to panitumumab rechallenge at the first tumor assessment. These patients will be included in the Safety Evaluation Population. Therefore, at the end of the study 31 patients will have received the rechallenge with panitumumab.

14.2 Statistical Analysis

14.2.1 Analysis Populations

Screening Phase. All patients signing the informed consent will be registered. The number of patients who will not be eligible will be recorded together with the reasons for non-eligibility.

The number of patients who died or withdrew before trial entry, will be specified.

Two populations will be considered for the analysis, as follows:

- The Safety Evaluable (SE) population defined as all treated patients (i.e. eligible as decided at the time of registration that receives at least 1 dose of study treatment). An incorrect treatment schedule or drug administration or an early termination of treatment does not result in exclusion of patients from this population. This population will be the object of the Safety and Tolerability analysis. Patients with major deviations from the eligibility criteria affecting safety or from the treatment schedule at cycle 1 for reasons other than toxicity may be presented in separate tables/listings. In this population are included the four patients already enrolled with protocol criteria v.2.1 and the 27 patients to be enrolled with protocol v.3.0 criteria.
- The Efficacy Evaluable (EE) population defined as all treated patients, with no major deviations from the eligibility criteria affecting efficacy evaluation, for which the tumor response could be evaluated at least once while on treatment. These patients should have received at least 2 cycles after treatment starts, unless disease progression occurs within this period. Patient with tissue DNA alteration (see also section 8.3.2 and appendix B) will be analyzed by the intention-to-treat (ITT) approach and will be included in the Efficacy Evaluable Population. A patient will be considered to be Trial eligible if he/she did not have any deviation from the patient entry criteria listed in sections 8.1 and 8.2 Potential eligibility problems will be assessed by the Study Coordinator at time of medical review. The final efficacy analysis will occur after 27 patients are evaluable for response according to RECIST 1.1. The distribution of follow-up time will be described and the number of patients lost to follow-up will be reported.

Conclusions will be based on all eligible patients. Further analysis may be performed excluding those patients for whom major protocol deviations have been identified (e.g., early death due to other reasons, early discontinuation of treatment, major protocol violations, etc.). However, these sub-analyses may not serve as the basis for drawing conclusions concerning treatment efficacy, and the reasons for excluding patients from the analysis should be clearly reported.

14.2.2 Statistical Methods

Binary variable such as ORR will be reported as proportions with 95% confidence interval (from the exact binomial distribution). Adverse events will be reported as proportions.

Time-to-event variables such as PFS and OS will be estimated using the Kaplan-Meier product-limit method. The full curve will be included in the report. Estimates at weekly intervals will be tabulated with their standard error; medians will be provided with 95% confidence interval. In case of explorative subgroup analysis to determine a potential association between the different (if any) RAS-extended mutant clones in plasma and response to panitumumab, we will use the log-rank test to estimate the significance of the comparison. Results will be considered significant when P values are 0.05 or less; Fisher's exact test will be used for subgroup comparisons of categorical variables. For the translational exploratory objectives, receiver operating characteristic (ROC) curve analysis might be used to assess whether fractional abundance of the mutational load is a potential classifier. In case, to quantify the discriminatory accuracy of the mutational as a predictor of efficacy outcomes, we will use Harrell C statistics Area under the curve analysis and a recursive partitioning method for detecting the best cut-off points[17]. Analyses will be performed with the use of SAS statistical software, version 9.2 (SAS Institute) and Stata version 12.

15 QUALITY CONTROL AND QUALITY ASSURANCE

15.1 Monitoring

Monitoring visits to the trial site will be made periodically during the trial by a qualified monitor to verify that the trial is conducted according to study protocol, GCP principles and regulatory requirements. The monitor will verify the accurate and complete recording of data on CRFs, source documents, Investigators File and drug accountability records.

The investigator/institution guarantees direct access to source documents of the study patients and to any other trial related documentation.

It is important that the investigator(s) and/or their relevant personnel are available during the monitoring visits.

15.2 Auditing

Representative members of Sponsor Quality Assurance may conduct an on-site audit. The investigator will be informed if an audit is to take place.

Representative of Regulatory Agencies may also conduct an inspection of the study. If informed of such an inspection, the Investigator should notify Sponsor immediately. The investigator will ensure that the auditors/ inspectors have access to the clinical supply, study site facilities, source documents and all study files.

16 DATA HANDLING AND RECORD KEEPING

16.1 Case Report Form (CRF)

An electronic Case Report Form will be completed for each enrolled subject. The language used must be English. The completed original Case Report Forms are the sole property of Sponsor and should not be made available in any form to third parties, except for authorized representatives of appropriate regulatory authorities, without written permission from Sponsor.

The Investigator or an authorized staff member (medically qualified) has the responsibility to ensure completion and to review and sign all Case Report Forms.

However, the Investigator has final personal responsibility for the accuracy and authenticity of all clinical and laboratory data entered on the Case Report Form.

Subject source documents are the hospital subject records maintained at the study site. In case where the source documents are the hospital chart, the information collected on the Case Report Form must match with those charts. In some case a portion of the source documents are not the hospital subject records. The investigator and Sponsor must agree which items will be recorded in the source documents and for which items the Case Report Form will stand as the source document. This must be stated in the “Data Location List” (filed in the Investigator File). One copy of this document should be remitted to the sponsor or CRO delegated by the sponsor (CD Pharma, Milano) for filing into the Trial Master File.

16.2 Data Handling

Data Management will be carried out by external CRO (CD Pharma, Milano). Medical terms are coded according to the MedDRA dictionary. Data will be analyzed using SAS® System currently used at CRO (TBD). Data cleaning will include both visual and computer-driven procedures in order to minimize logical inconsistencies and errors within the collected data. The data are checked for completeness, accuracy and consistency. The errors detected will be rectified by means of Data Clarification List (DCL) that will be used by the monitor for resolution of queries. The original DCL must be kept together with the patient CRF.

16.3 Record Retention

To enable evaluation and/or audits and/or regulatory authorities inspections, the Investigator agrees to keep records, including the identity of all participating subjects (“Subject identification Log”), all original signed informed consent forms, copies of all case report forms, source documents, detailed records of treatment disposition as well as the documentation included in the Investigator File according to local regulations or as specified in the Clinical Trial Agreement.

If the Investigator relocates, retires, or for any reason withdraws from the study, Sponsor should be prospectively notified. The study records must be transferred to an acceptable designee, such as another investigator, another institution, or to CRO (CD Pharma, Milano). The investigator must obtain Sponsor’s written permission before disposing of any records.

17 ETHICAL CONSIDERATIONS

17.1 Institutional Review Board (IRB)/ Independent Ethics Committee (IEC) and Competent Authority (CA)

Before initiating the trial, the Investigator should have written favourable opinion from the IRB/IEC and CA for the trial conduction. All the correspondence with the IRB/IEC and CA should be retained in the Investigator File.

Before implementing any protocol amendment, the IRB/IRC/CA written approval must be obtained. The only circumstance in which an amendment may be initiated prior to IRB/IEC approval is where the change is necessary to eliminate apparent immediate hazards to the subjects. In that event, the IRB/IEC/CA must be notified in writing *asap*.

It is responsibility of Sponsor to provide the Investigator with the Health Authority approval where needed to implement a trial.

17.2 Ethical conduct of the trial

The trial will be performed in accordance with International Conference on Harmonization Good Clinical Practice guidelines, the Declaration of Helsinki and applicable local regulatory requirements and laws.

17.3 Informed Consent

It is the responsibility of the investigator to give each patient full and adequate verbal and written information regarding the objective and procedures of the trial and the possible risks involved. The patient must be informed about his/her right to withdraw from trial at any time. The patient should have time and opportunity to enquire about details of the trial and to decide whether or not to participate in the trial.

Written subject information must be approved by IRB/IEC and CA and must be given to each patient before any trial-related procedure is undertaken.

It is responsibility of the investigator to obtain informed consent signed and dated by the patient and by the medical person conducting the informed consent discussion, prior to undertaken any trial-related procedure. One copy of the signed and dated Informed Consent Form should be given to the

patient. The originally signed document should be archived in the confidential section of the Investigator File.
The approved patient information sheet must not be changed without prior approval by Sponsor and by the IRB/IEC and CA.
When new study information arises during the study, the patients still on treatment must be informed and a new Informed Consent form or an addendum to the already signed Informed Consent form must be signed and dated by the patients.
If a patient becomes incompetent during the course of a trial where it was not anticipated, legally acceptable representative authorization should be obtained for a subject's continued participation.

18 LIABILITY AND INSURANCE

The involved parties will be insured in accordance with the applicable laws and regulation for injuries and/or damages that may arise as a consequence of this trial.

19 CONFIDENTIALITY OF INFORMATION AND PUBLICATION OF RESULTS

Sponsor assures that the key design element of this protocol will be posted in a publicly accessible database such as clinicaltrial.gov; in addition, upon study completion, the results of this study will be submitted for publication and posted in publicly accessible database for clinical trial studies.
All information regarding study drug supplied by Sponsor to the investigator is privileged and confidential information. The investigator agrees to use this information to accomplish the study and will not use it for other purposes without consent from Sponsor.
It is understood that there is an obligation to provide Sponsor with complete data obtained during the study. The investigator agrees to keep in confidence all the results obtained from the study. Such information shall not be disclosed to third parties without prior written permission from Sponsor, except to regulatory authority(ies), when requested.
Individual investigators may present results of the study at scientific meetings. However prior to the submission, the Sponsor will have the opportunity to review and comment the abstracts for a period of up to 15 calendar days prior to the submission.

20 FIGURES

20.1 Figure 1 Mutated KRAS alleles emerge in circulating DNA during anti-EGFR therapy and decline when treatment is suspended

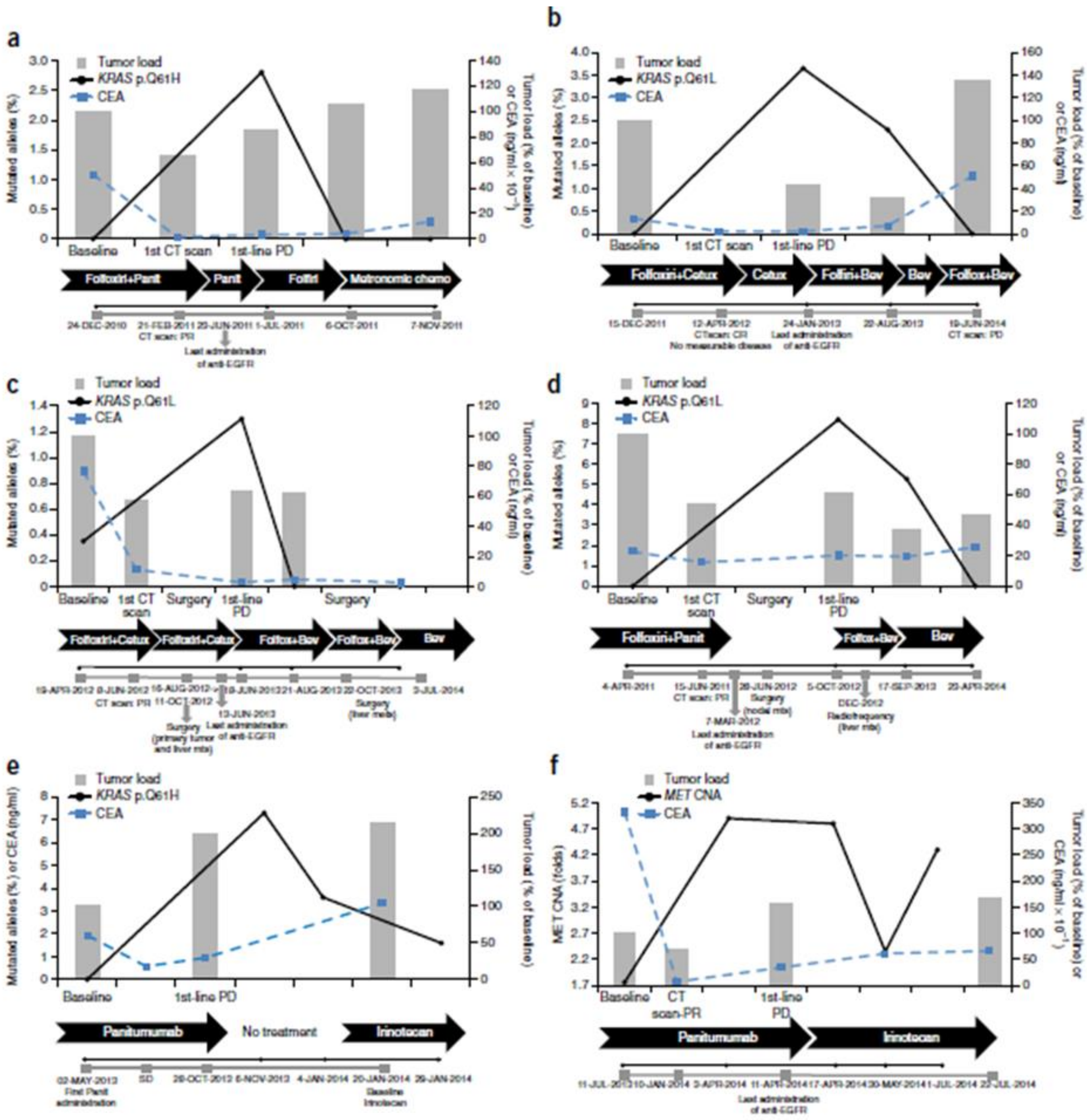

(a–f) Detection of KRAS mutations in mCRC patients AOUP-CRC04 (a), AOUP-CRC01 (b), AOUP-CRC06 (c), AOUP-CRC03 (d), ONCG-CRC71 (e) and MET amplification in patient ONCG-CRC72 (f) in circulating DNA of patients who developed acquired resistance to first-line chemotherapy plus anti-EGFR treatment and then received other lines of treatment. Gray bars represent the variation of tumor load, compared to baseline, during systemic treatments specified in arrows below the graphs. Tumor load is calculated as follows: measurable disease at the initiation of treatment (baseline) is assumed as 100%; responses or progression are calculated as the percentage of tumor load compared to baseline, as per Response Evaluation Criteria in Solid Tumors (RECIST) criteria. Relevant clinical events are indicated in gray boxes below the graphs. Black lines indicate the frequency of

KRAS mutation (percentage of alleles) or MET copy number alteration, detected in circulating DNA at the time points indicated below the graphs. Dotted blue line indicates CEA (carcinoembryonic antigen) values. PD, progressive disease; Cetux, cetuximab; Panit, panitumumab; Bev, bevacizumab; Irino, irinotecan; Folfoxiri, folinic acid, 5-fluorouracil, oxaliplatin and irinotecan; Folfiri, folinic acid, 5-fluorouracil and irinotecan; Folfox, folinic acid, fluorouracil and oxaliplatin.

20.2 Figure 2 Re-challenge with EGFR specific antibodies in CRC cells and patients

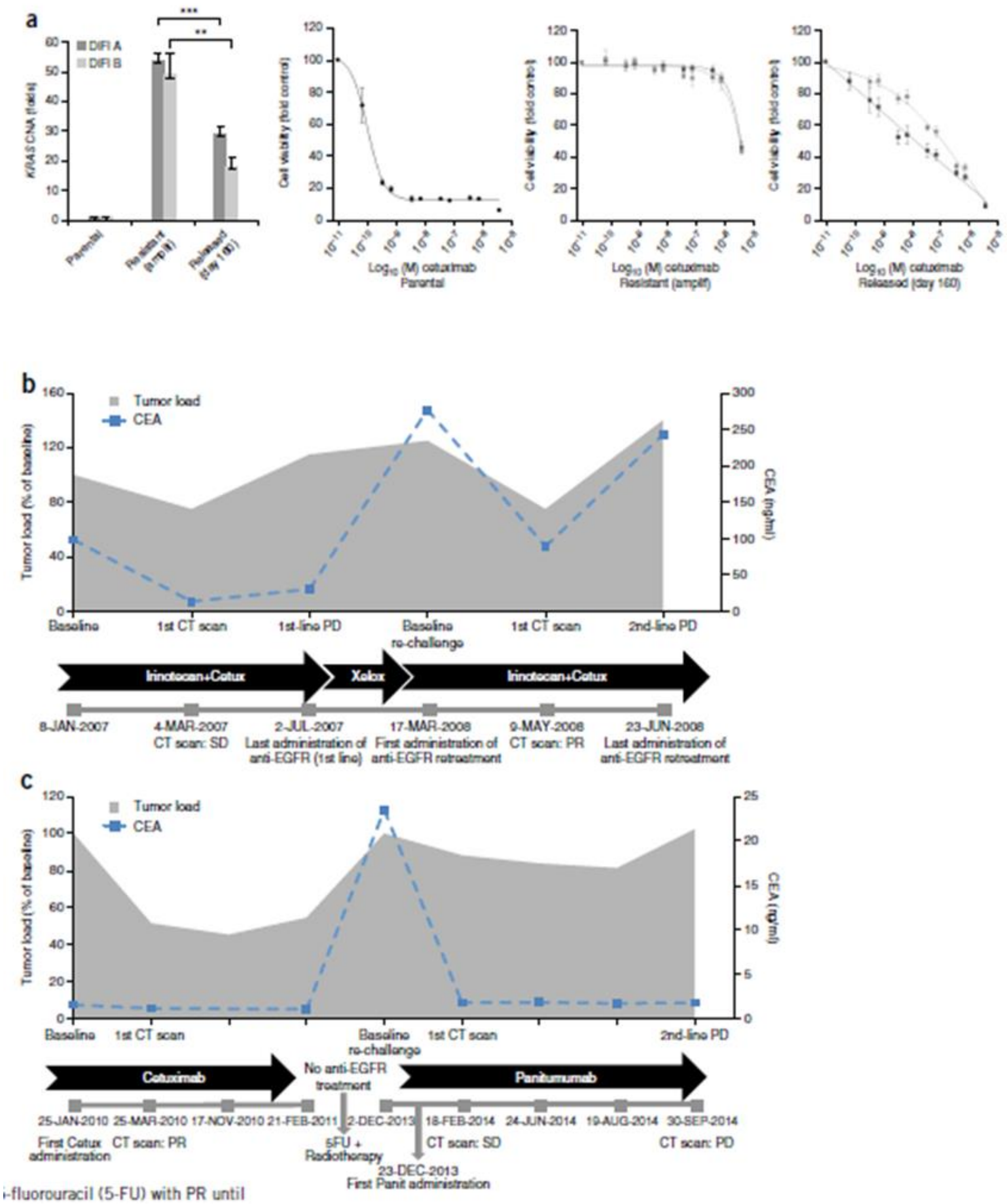

(a) Two CRC cell populations (DiFi A and DiFi B) that developed KRAS amplification as a resistance mechanism to cetuximab were allowed to replicate in the absence of the antibody for 160 d. Top, KRAS amplification assessed by qPCR in the indicated cell models (parental/sensitive, resistant derivatives and resistant cells after 160 d of antibody withdrawal). Gray bars indicate KRAS gene copy number alterations. Statistical differences were

calculated by Student's t-test. Data are expressed as means ± s.d. of three independent experiments. ***P ≤ 0.001; **P ≤ 0.01. Bottom, cetuximab sensitivity assay. Data points represent means ± s.d. of three independent experiments.

(b) Clinical synopsis of mCRC patient HMAR-CRC07 treated with irinotecan plus cetuximab achieving stable disease (SD) for approximately 6 months. At progression, the patient received capecitabine plus oxaliplatin (Xelox) with further progression of the disease after 3 months. The patient was subsequently re-treated with irinotecan plus cetuximab, achieving a partial response (PR). Gray area represents tumor load (percentage of baseline, calculated as described in Fig. 1 legend); dotted blue line indicates CEA (carcinoembryonic antigen) values.

(c) Clinical synopsis of mCRC patient ONCG-CRC74, who was treated with cetuximab as a third-line therapy, achieving a partial response that lasted 13 months; the patient then refused further therapy because of skin toxicity. At disease progression, the subject underwent radiotherapy and treatment with 5-fluorouracil (5-FU) with PR until progression occurred after 6 months. The patient was re-challenged with anti-EGFR treatment, achieving long-lasting stable disease (7 months). Gray area represents tumor load (percentage of baseline, calculated as described in Fig 1 legend); dotted blue line indicates CEA values. Cetux, cetuximab.

20.3 Figure 3 Study Flow Chart

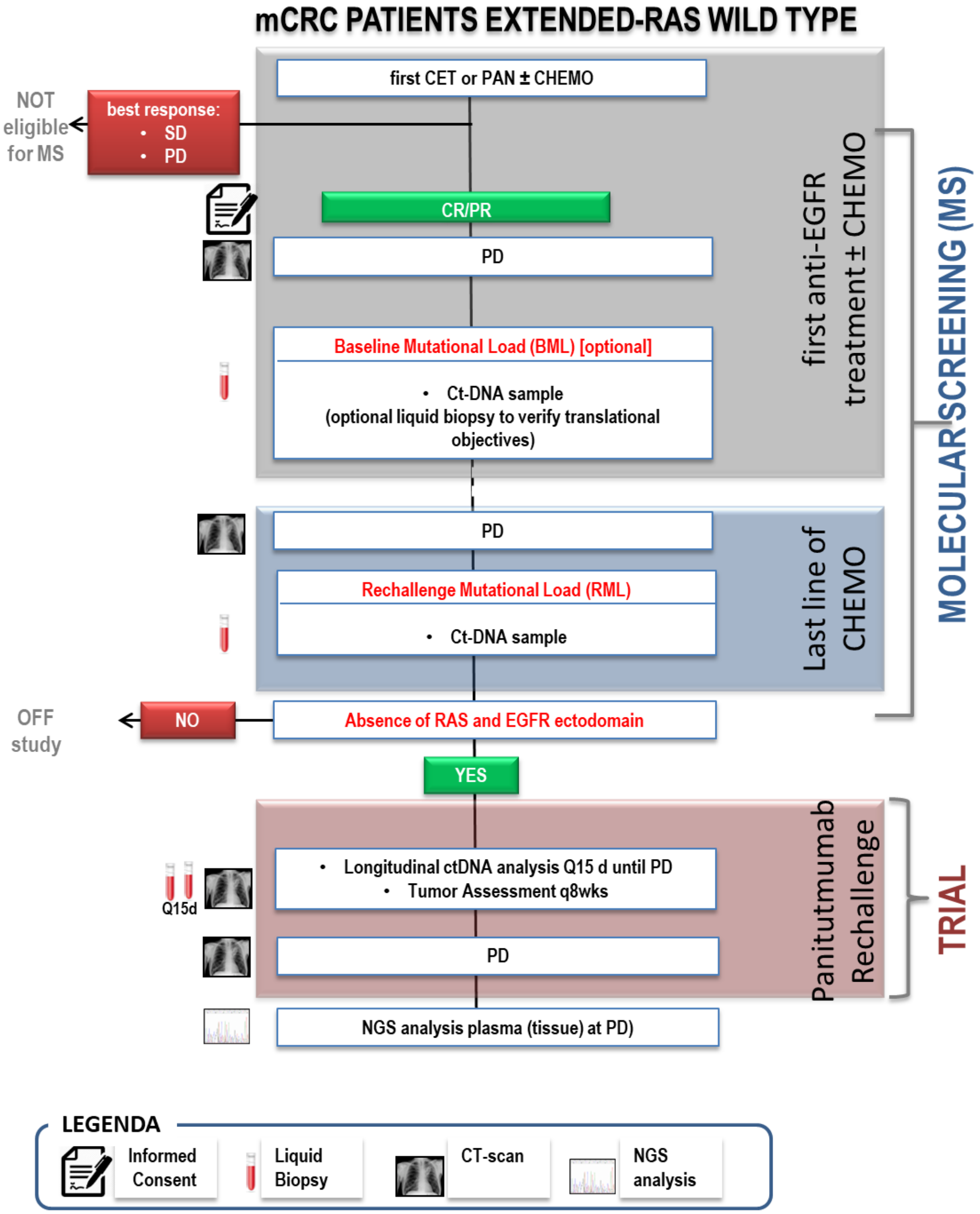

21 APPENDIX

21.1 Appendix A: References

1 Siegel, R., Naishadham, D. & Jemal, A. Cancer statistics, 2013. *CA Cancer J Clin* **63**, 11-30, doi:10.3322/caac.21166 (2013).

2 Heinemann, V. *et al.* FOLFIRI plus cetuximab versus FOLFIRI plus bevacizumab as first-line treatment for patients with metastatic colorectal cancer (FIRE-3): a randomised, open-label, phase 3 trial. *Lancet Oncol* **15**, 1065-1075, doi:10.1016/S1470-2045(14)70330-4 (2014).

3 Fakih, M. G. Metastatic colorectal cancer: current state and future directions. *J Clin Oncol* **33**, 1809-1824, doi:10.1200/JCO.2014.59.7633 (2015).

4 Siena, S., Sartore-Bianchi, A., Di Nicolantonio, F., Balfour, J. & Bardelli, A. Biomarkers predicting clinical outcome of epidermal growth factor receptor-targeted therapy in metastatic colorectal cancer. *J Natl Cancer Inst* **101**, 1308-1324, doi:10.1093/jnci/djp280 (2009).

5 Misale, S., Di Nicolantonio, F., Sartore-Bianchi, A., Siena, S. & Bardelli, A. Resistance to anti-EGFR therapy in colorectal cancer: from heterogeneity to convergent evolution. *Cancer Discov* **4**, 1269-1280, doi:10.1158/2159-8290.CD-14-0462 (2014).

6 Arena, S. *et al.* Aurora kinase A gene copy number is associated with the malignant transformation of colorectal adenomas but not with the serrated neoplasia progression. *Clin Cancer Res.* **21**, 2157-2166. doi: 2110.1158/1078-0432.CCR-2114-2821. Epub 2015 Jan 2126. (2015).

7 Misale, S. *et al.* Emergence of KRAS mutations and acquired resistance to anti-EGFR therapy in colorectal cancer. *Nature* **486**, 532-536, doi:10.1038/nature11156 (2012).

8 Diaz, L. A. *et al.* The molecular evolution of acquired resistance to targeted EGFR blockade in colorectal cancers. *Nature* **486**, 537-540, doi:10.1038/nature11219 (2012).

9 Siravegna, G. *et al.* in *Nat Med* Vol. 21 827 (2015).

10 Arena, S. *et al.* Emergence of Multiple EGFR Extracellular Mutations during Cetuximab Treatment in Colorectal Cancer. *Clin Cancer Res* **21**, 2157-2166, doi:10.1158/1078-0432.ccr-14-2821 (2015).

11 Santini, D. *et al.* Cetuximab rechallenge in metastatic colorectal cancer patients: how to come away from acquired resistance? *Ann Oncol*, doi:10.1093/annonc/mdw551 (2017).

12 Pietrantonio, F. *et al.* Single agent panitumumab in KRAS wild-type metastatic colorectal cancer patients following cetuximab-based regimens: Clinical outcome and biomarkers of efficacy. *Cancer Biol Ther* **14**, 1098-1103, doi:10.4161/cbt.26343 (2013).

13 Arena, S. *et al.* Emergence of Multiple EGFR Extracellular Mutations during Cetuximab Treatment in Colorectal Cancer. *Clin Cancer Res* **21**, 2157-2166, doi:10.1158/1078-0432.CCR-14-2821 (2015).

14 Van Emburgh, B. O. *et al.* Acquired RAS or EGFR mutations and duration of response to EGFR blockade in colorectal cancer. *Nat Commun* **7**, 13665, doi:10.1038/ncomms13665 (2016).

15 Parseghian, C. M. *et al.* Anti-EGFR Resistant Clones Decay Exponentially After Progression: Implications for Anti-EGFR Re-challenge. *Ann Oncol*, doi:10.1093/annonc/mdy509 (2018).

16 A'Hern, R. P. Sample size tables for exact single-stage phase II designs. *Stat Med* **20**, 859-866, doi:10.1002/sim.721 (2001).

17 Harrell, F. E., Lee, K. L. & Mark, D. B. Multivariable prognostic models: issues in developing models, evaluating assumptions and adequacy, and measuring and reducing errors. *Stat Med* **15**, 361-387, doi:10.1002/(SICI)1097-0258(19960229)15:4<361::AID-SIM168>3.0.CO;2-4 (1996).

21.2 Appendix B: minimum set of genes to be tested on tissue FFPE

If not already performed, patients will be tested for negativity to the following minimum set of gene mutations and CNV within 3 months from the treatment C1D1. Genotyping can be performed trough any multigene platform (i.e. Foundation Medicine platform, Personal Genomic Diagnostic platform, MSK-IMPACT platform) at diagnosis of metastatic disease, during study screening or within 3 months from treatment C1D1.

Negativity to this panel is defined as the absence of any DNA mutation and no CNV ≥ 10 in the following loci/genes.

Genotyping can be perfomed on archival tumour samples (not older than 1 year) or on a freshly obtained tumor biopsy. Participating center that cannot perform Genotyping will send FFPE to Istituto di Candiolo-IRCCS for centralized testing.

CHRONOS minimum panel of genes for multiplex screening

GENE	MUTATION FOR SCREENING (SEQUENOM PLATFORM)
BRAF	G464R, G464V/E, G466R, F468C, G469S, G469E, G469A, G469V, G469R, D594G, D594V, V600E (1799T>A), V600K, V600R (1798_1799GT>AG), V600M, V600Q, V600E (1799_1800TG>AA) / V600D (1799_1800TG>AC/1799_1800TG>AT), V600A, V600G, V600L (1798G>C), V600R (1797_1799AGT>GAG), V600>YM, V600_K601>E, K601E,
EGFR	S492R, G465E/R R108K, T263P, A289V, G598V, E709K/H, E709A/G/V, G719S/C, G719A, M766_A767insAI, S768I, V769_D770insASV, V769_D770insCV, D770_N771>AGG/V769_D770insASV, D770_N771insG, N771_P772>SVDNR, P772_H773insV, H773>NPY, H773_V774insNPH/PH/H, V774_C775insHV, T790M, L858R, L861Q, E746_T751del, E746_A750del, S752D, L747_E749del, L747_T750del, L747_T751del, L747_S752del, P753S, A750P, T751A, T751P, T751I, S752I/F, S752_I759del, L747_Q ins, E746_T751del, I ins (combined), E746_A750del, T751A (combined), L747_E749del, A750P (combined), L747_T750del, P ins (combined), L747_S752del, Q ins (combined)
HER2	L866M, V777L, S310Y
IDH1	R132C
KRAS	G12D (35G>A), G12V (35G>T), G12C (34G>T) , G12A , G12S , G12R (34G>C), G12F, G12L, G12I, G12fs*3, G12E (35_36GT>AA/35_36GT>AG), G12Y, G12N, G13D (38G>A), G13C (37G>T), G13S, G13R, G13A, G13V (38G>T), A59E, A59G, A59T, A59S, A59P, A59V, Q61H (183A>C), Q61H (183A>T), Q61L, Q61R (182A>G), Q61K (181C>A), Q61P, Q61E, K117N (351A>C), K117N (351A>T), K117Q, K117E, K117R, K117T, K117I, A146T, A146P, A146V, A146G, A146S, A146E,
MEK1 (MAP2K1)	K57N
NRAS	G12V/A/D, G12C/R/S, G13V/A/D, G13C/R/S, A18T, Q61L/R/P, Q61H, Q61E/K
PIK3A	R88Q, N345K, C420R, P539R, E542K, E545K, Q546K, H701P, H1047R/L, H1047Y, R38H, C901F, M1043I

GENES FOR CNV (NANOSTRING PLATFORM)
CRAF(RAF1)
EGFR
FGFR1
FGFR2
FGFR3
HER2
IGF1
IGF1R
IGF2
KRAS
MET
NF1 (deletion)

21.3 Appendix C: ddPCR Panel for Molecular Screening

ddPCR Panel for Molecular Screening														
Gene	*KRAS*													
Mutation	G12A	G12C	G12D	G12R	G12S	G12V	G13D	Q61K	Q61L	Q61R	Q61H (A>T)	Q61H (A>C)	Q61P	Q61E
Gene	*NRAS*													**BRAF**
Mutation	G12A	G12C	G12D	G12R	G12S	G12V	G13D	Q61K	Q61L	Q61R	Q61H (A>T)	Q61H (A>C)		V600E
Gene	*EGFR*													
Mutation	S492R (C>A)	G465R (G>A)	G465R (G>C)	G465E (G>A)	S464L (C>T)	V441G (T>G)	V441D (T>A)							

21.4 Appendix D: Liquid Biopsy Guidelines for Molecular Screening Phase

Health and safety

In accordance with the site's policies and guidelines, use personal protective equipment to prevent exposure to blood borne pathogens or other potentially infectious materials, and dispose of all clinical waste appropriately. Before starting to work under this protocol, all staff should review the guidelines for working with blood borne pathogens and have been vaccinated.

Materials and Equipment

Cell-Free DNA BCT® Streck Tubes (Streck, catalogue number 218997) will be used to collect blood samples. Streck Tubes must be stored at controlled room temperature (6°-30°C).

21.5 Appendix E: Liquid Biopsy Guidelines for Trial Phase

These instructions describe how to collect whole blood samples and prepare plasma for the isolation of cell-free circulating tumor DNA (step 1)
PBMC isolation guidelines from the same blood samples are described below (step 2).

Health and safety

In accordance with the site's policies and guidelines, use personal protective equipment to prevent exposure to blood borne pathogens or other potentially infectious materials, and dispose of all clinical waste appropriately. Before starting to work under this protocol, all staff should review the guidelines for working with blood borne pathogens and have been vaccinated.

All steps to be carried out under a laminar flow sterile hood.

Materials and Equipment for step 1

- 10 ml K2-EDTA Vacutainer (Becton Dickinson, # 366643 or equivalent)
- Vacutainer needles or butterfly needles, 20G/21G (Becton Dickinson, #367344/364815 or equivalent)
- 1.5ml APEX Screw-Cap Microcentrifuge Tube, Conical, Standard Cap, Sterile #APCP5931
- 10 ml and 5 ml serological disposable pipettes (Corning, # 4487 or #4488 or equivalent)
- 15 ml polypropylene centrifuge tubes (Fisher, # 3208303 or equivalent)

- Freezer storage boxes for 2 ml cryogenic vials (Fisher, # 3468196 or equivalent)
- Centrifuge, capable of ~3000 g with a swing bucket rotor (e.g. Eppendorf, 5702; # 5702 000.019 or equivalent)
- Pipetting aid (e.g. Eppendorf, Easypet; #4421 000.013 or equivalent)
- Ultra-Low Temperature Freezer (e.g. Thermo Electron, Revco Ultima PLUS; ULT1786-10 or equivalent)

Materials and Equipment for step 2

- Lysis buffer 10X: (NH4Cl 1,55M, KHCO3 100mM, EDTA 10mM)
- H2O infusion grade.
- Freezing mix: 80% FCS + 20% DMSO
- Turk Staining
- Centrifuge tube 15 and 50mL
- Serological pipette, micropipette and pipetting aid.
- Burker chamber

Lysis Buffer recipe

NH_4Cl 1,55M	= 41,45gr
$KHCO_3$ 100mM	= 5gr
EDTA 10mM	= 1,85gr
H_20	to 500ml

Procedure

Blood Draw (in Clinic)

1. Confirm subject's ID and write subject's name or subject ID and DOB on the sample sheet and EDTA tube.
2. Prepare subject for blood draw.
3. Obtain venous blood (~1 to 2 times10 ml) by any standard phlebotomy technique from a peripheral access point or from a central line by trained personnel into EDTA tubes.
4. For special instructions, see EDTA tube product information.
5. Gently invert tubes about 10 times immediately after collection.
6. Record date and time of blood draw on samples sheet and EDTA tube.
7. Prepare sample for the transportation to the laboratory or processing side.

Time between blood collection and plasma/blood cell processing is recommended to be <4-5 h. Experiments have shown that extended storage at room temperature ≥5h can affect the detection of cell-free circulating tumor DNA in plasma.

STEP 1

Plasma Processing (in Laboratory)

8. Upon arrival in the laboratory, centrifuge EDTA tubes at room temperature for 10 min at 1600 (±150) g. If centrifuge uses rpm (revolutions per minute), see centrifuge instructions for the conversion.
9. Ensure that brake switch is off in order to prevent disruption of the cell layer.
10. Record plasma processing start time at start of first centrifugation.
11. After centrifugation remove tubes from centrifuge.
12. Transfer supernatant of the two EDTA tubes to one fresh 15 ml centrifuge tube without disturbing the cellular layer using a disposable 10 ml serological pipette or disposable bulb pipette. Do not discard the EDTA tubes, as they will be used for PBMC extraction described in step 2.

Centrifugation separates plasma from leukocytes and erythrocytes as shown in the figure below (left). Leaving sufficient residual plasma in the tubes after the centrifugation and not disturbing the leukocyte layer (see image) when pipetting is a critical step in the sample preparation process (right). Be careful not to disturb leukocyte layer in the tubes.

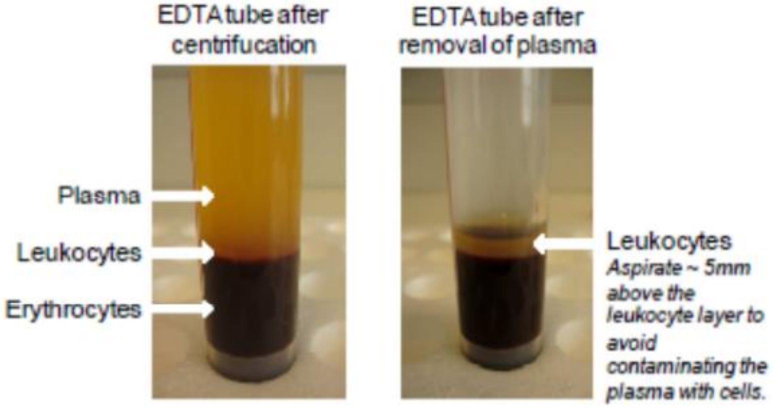

13. Centrifuge the plasma in the 15 ml centrifuge tube at room temperature for 10 min at 3000 (±150) g.

14. After centrifugation remove tubes from centrifuge.
15. Transfer supernatant to a fresh 15 ml centrifuge tube without disturbing the cellular layer using a disposable 5 ml or 10 ml serological pipette or disposable bulb pipette.

The 2nd centrifugation is intended to remove any residual intact blood cells carried over from the 1st centrifugation step. Tests have shown that speeds slower than 3000g do not completely remove blood cells from the supernatant. Centrifugation speeds higher than 3000 g are preferred.

16. Leave a residual volume of about 0.3 ml (~7 mm) on the bottom of the 15 ml tube to avoid contaminating the plasma with cells (see image).

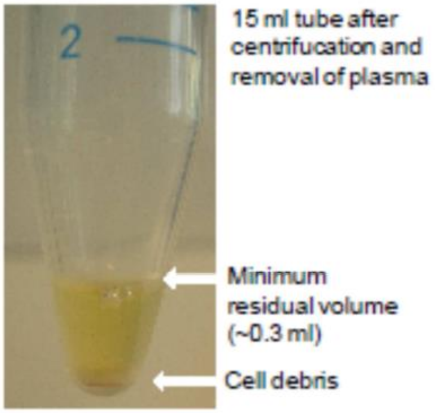

17. After transferring the plasma to a new 15 ml centrifuge tube as described, gently mix plasma and record total plasma volume (~8-10 ml plasma per 20 ml blood).
18. Transfer 1 ml plasma aliquots with a pipette to 1.5ml APEX Screw-Cap Microcentrifuge Tube
19. Place plasma tubes into storage box and freeze plasma in freezer upright in storage box at -70°C or colder.
20. Short time storage at -20°C is possible.

STEP 2

PBMC extraction

**Start from EDTA tubes of step 1 (point 12) after having removed the surnatant (plasma)**

21. With a micropipette transfer all the buffy coat (the leukocyte layer above the erythrocytes) to a new 1,5mL Eppendorf.
22. Resuspend the cell in ≈1,2mL of lysis buffer 1X (dilute lysis buffer 10X in water) and mix thoroughly (vortex).
23. Wait 7 minutes (room temperature) or until the mix looks clear (the mix becomes clear when the erythrocyte are lysed).
24. Centrifuge 5 minutes at 1500rpm.
25. Remove supernatant with a pipette without disturbing the pellet and resuspend it in ≈1,2mL of lysis buffer (wash).
26. Centrifuge again for 5 minutes at 1500rpm.
27. Remove supernatant with a pipette without disturbing the pellet and resuspend it in 500µL of FCS.
28. Count the cells in a Burker Chamber with a vital stain (Turk) and take note of the results. (approx. $15x10^6$ cells will be harvested from each 10 mL blood sample)
29. Add 500µL of Freezing mix
30. Transfer the Eppendorf in a criobox and let it freeze to -80°C overnight.

Specimen Storage Instructions

- Once frozen, maintain samples continuously at -70°C or colder.
- When outside the freezer, such as when transferring to a different freezer in another location or preparing for shipment, boxes containing tubes should be covered with dry ice.
- Freezer or dry ice specimen storage container temperature must be checked and documented at least once each workday. Document any deviation from protocol. The freezer or dry ice storage box containing the specimens should either be locked or in a secure area accessible only to authorized site staff.
- A backup storage plan should be in place in the event of freezer failure. Ship frozen samples according to Inostics' Specimen Shipment Instructions.

Supplementary Material Appendix 2.

Recruiting centers, principal investigators, and enrolled patients.

**Recruiting Center**	**Patients enrolled**
Niguarda Cancer Center, Grande Ospedale Metropolitano Niguarda, Milano, Italy - PI Dr. Andrea Sartore-Bianchi -	11
Fondazione IRCCS Istituto Nazionale dei Tumori, Milan, Italy - PI Prof. Filippo de Braud-	8
Veneto Institute of Oncology (IOV)-IRCCS Padua, Italy - PI Dr. Sara Lonardi -	7
Istituto di Candiolo, Fondazione del Piemonte per l'Oncologia, IRCCS, Candiolo, Italy - PI Prof. Massimo Aglietta -	1
POLICLINICO UNIVERSITARIO CAMPUS BIOMEDICO - PI Prof. Giuseppe Tonini -	0
HUMANITAS Research Hospital, Milano - Dr. Lorenza Rimassa -	0
**TOTAL**	**27**

Supplementary Material Appendix 3.

Inclusion and exclusion criteria Protocol version 3.0.

Inclusion Criteria

1 Histologically confirmed diagnosis of metastatic colorectal cancer;
2 Age ≥ 18 years;
3 Written informed consent;
4 Documented WT RAS exons 2, 3 and 4 (KRas and NRas) and WT BRAF V600E for anti-EGFR treatment.
5 Complete or partial response to anti EGFR antibodies in any line–either received as monotherapy or in combination with chemotherapy;
6 Imaging documented progression while on therapy with a therapeutic regimen including anti-EGFR mAb;
7 Imaging documented progression at the last treatment regimen that must be anti-EGFR free;
8 Patient must be RAS and EGFR ectodomain wild type in a liquid biopsy performed no longer that 4 weeks after progression to the last anti-EGFR free treatment

9 FFPE sample used for eligibility to anti-EGFR prescription (see criteria 4) must be available for custom gene panel profiling (as described in appendix B). Otherwise, if sample is not available, center must have already perfomed a genotyping on this tissue sample according to appendix B.

10 ECOG performance status ≤ 2;
11 At least one measurable tumor lesion as per RECIST v1.1. Lesions in previously irradiated areas or those that have received other loco-regional therapies (i.e. percutaneous ablation) should not be considered measurable unless there is clear documented evidence of progression of the lesion since therapy. Imaging must be performed maximum within 28 days prior to registration;
12 Normal organ functions.

13 Negative serum pregnancy test within 1 week prior to the first study dose in all women of childbearing potential;

14 Subjects and their partners must be willing to avoid pregnancy during the trial. Male subjects with female partners of childbearing potential and female subjects of childbearing potential must, therefore, be willing to use adequate contraception;

15 Absence of any psychological, familial, sociological or geographical condition potentially hampering compliance with the study protocol and follow-up schedule; those conditions should be discussed with the patient before registration in the trial.

Exclusion Criteria

1. History of severe infusion reactions to monoclonal antibodies cetuximab or panitumumab;
2. Symptomatic or untreated leptomeningeal disease and symptomatic brain metastasis;
3. Clinically significant cardiac disease including:

   a. congestive heart failure requiring treatment (NYHA grade ≥ 2), Left ventricular ejection fraction (LVEF) < 45% as determined by Multigated acquisition (MUGA) scan or echocardiogram;

   b. history or presence of clinically significant ventricular arrhythmias or atrial fibrillation;

   c. clinically significant resting bradycardia;

   d. unstable angina pectoris ≤ 3 months prior to starting study drug;

   e. acute myocardial infarction ≤ 3 months prior to starting study drug;

   f. QTcF > 480 msec;

4. History of thromboembolic or cerebrovascular events within the last 6 months, including transient ischemic attack, cerebrovascular accident, deep vein thrombosis, or pulmonary embolism;
5. Patients with interstitial pneumonitis or pulmonary fibrosis;
6. Abnormal organ or bone marrow functions defined as:

a. Absolute neutrophil count < 1.5 x 10/L;

b. hemoglobin < 9 g/dL;

c. alkaline phosphatase > 2.5 x upper normal limit (ULN), if liver metastases > 5 x ULN;

d. aspartate aminotransferase (AST)/ alanine aminotransferase (ALT) > 2.5 x ULN, if liver metastases > 5 x ULN;

e. bilirubin > 1.5 x ULN, if liver metastases > 2 x ULN;

f. serum creatinine > 1.5 x ULN and/or creatinine clearance ≤ 50 mL/min calculated according to Cockroft-Gault;

g. Patients with platelet count <100 x 10^9/L

7. Previous or concurrent second malignancy. Exceptions: adequately treated basal cell or squamous cell skin cancer; in situ carcinoma of the cervix, treated curatively and without evidence of recurrence for at least 3 years prior to study entry; or other solid tumor treated curatively and without evidence of recurrence for at least 3 years prior to study entry.
8. Patients with positive serology for HIV, HBV, HCV.
9. Patients with a history of severe or life-threatening hypersensitivity to the active substance or to any of the excipients.

Supplementary Material Appendix 4.

Protocol deviations.

Patient ID	Protocol deviation
CAN-002	Exclusion criteria n. 8. (HBV, no active infection but Ab positive)
IOV-002	Procedures deviation (Baseline TC > 28 d. Protocol schedule)
IOV-007	Procedures deviation (Baseline TC > 28 d. Protocol schedule)

Supplementary Material Appendix 5.

Report of the independent review of radiologic responses of CHRONOS trial.

Report of the Independent Review of radiologic responses of CHRONOS trial

Product Code:	Vectibix®(panitumumab)
Report of the peer review for study:	CHRONOS 013-IRCC-10IIS-16
EudraCT:	2016-002597-12
ClinicalTrials.gov:	NCT03227926
Date of First Subject Enrolled:	August 19, 2019
Date of Last Subject Enrolled:	November 6, 2020
Date of Data Base Lock:	July 31, 2021
Principal Investigator:	Salvatore Siena, MD<br>Niguarda Cancer Center<br>Milano - Italy
Development Phase of Study:	2

APPROVAL SIGNATURES

I have read the report and confirm that to the best of my knowledge it accurately describes the conduct and results of the study.

15/09/2021

Daniele Regge, MD — Date
Diagnostic, Radio-diagnostic Department
Fondazione del Piemonte per l'Oncologia-IRCCS
Candiolo (TO) - Italy

15/09/2021

Angelo Vanzulli, MD — Date
Director, Struttura Complessa Radiologia
Niguarda Cancer Center, Grande Ospedale Metropolitano Niguarda
Milano, Italy

TABLE OF CONTENTS

1. ABBREVIATIONS AND DEFINITION OF TERMS 2
2. OVERALL REVIEW CONDUCT 3
3. REVIEW PROCESS 3
3.1. Blind independent revision 3
3.2. Reconciliation process 3
3.3. Blind independent revision 3
4. REVIEW REULTS 4
4.1. Summary of the reconciliation results by patient 8
Patient INT-013 8
Patient INT-015 8
Patient IOV-003 8
Patient IOV-006 8
Patient IOV-007 8
Patient IOV-009 8
Patient IOV-010 8
Patient NIG-001 9
Patient NIG-002 9
Patient NIG-003 9
Patient NIG-004 9
Patient NIG-006 9
Patient NIG-015 9

1. ABBREVIATIONS AND DEFINITION OF TERMS

CR	Complete Response
CRF	Case Report Form
DOR	Duration of Response
FPI	First patient in
LD	Longest Diameter
NE	Not Evaluable
ORR	Objective Response Rate
PR	Partial Response
SD	Stable Disease
PFS	Progression free survival

2. OVERALL REVIEW CONDUCT

The review was conducted by two independent reviewers with the support of Telemis version 4.9 (Telemis SA, Louvain la Neuve, Belgium). Due to the pandemic restrictions, the final reconciliation was performed via teleconference on August, 31, 2021.

The reviewers were:

- Prof. Daniele Regge, Director of the FPO Department of Radio-diagnostics, Candiolo (TO), Italy

-Dr. Angelo Vanzulli, Head of the Struttura complessa Radiologia, Ospedale Niguarda Ca'Granda-Milan, Italy

Copies of CT-Scan of all evaluable patients were collected from each participating center and uploaded on Telemis version 4.9 (Telemis SA, Louvain la Neuve, Belgium) for evaluation.

Tumor assessments by protocol were required at baseline (within 4 weeks from study drug administration) and planned every 8 weeks thereafter until progressive disease was noted. The RECIST 1.1 criteria were used for tumor response evaluation by the local Investigators and by both Reviewers.

3. REVIEW PROCESS

3.1. Blind independent revision

All CT-scans for all patients in trial were assessed by RECIST 1.1 independently. Each reviewer, blinded to both the investigator and the other independent reviewer, recorded RECIST 1.1 assessment on an electronic form.

3.2. Reconciliation process

All the cases were uploaded on Telemis version 4.9 (Telemis SA, Louvain la Neuve, Belgium). The study Project Manager flagged all discordant in either assessment of response, and or differences in the length of time dependent variables (time to response; progression free survival; duration of response). Cases requiring reconciliation were discussed collegially via teleconference by the two reviewers.

3.3. Blind independent revision

Discrepancies and decision whether reconciliation was required are reported in Table 1 (best response) and table 2 (time dependent variables).

Five of 27 patients (INT-011, NIG-009, NIG-012, NIG-013 and NIG-014) were not included in the independent revision as the baseline examinations were not available. All the above-mentioned patients were in progression at the first re-assessment (8 weeks).

Overall, 13 (48%) patients were selected for the reconciliation process due to discrepancies in the assessment of best response (n=7; 26%), or length of time dependent variable (n=9; 33%); three patients (IOV-003, IOV-006, NIG-001) are included in both series.

4. REVIEW REULTS

The results of the reconciliation process are summarized Table 3 and 4.

Best responses assigned by the local investigators was modified by reviewers according to RECIST 1.1 in three cases: two cases were downgraded from PR to SD and one was upgraded from SD to PR.

Date of progression - leading to the shortening of the individual patients' time dependent variables - was anticipated in 7 (26%) cases for TTP and 4 (15%) cases for DOR.

Table 1: Discrepancies in the definition of best response

	Assessment			Type of discrepancy	Patient ID #	ACTION
	**INV**	**REV A**	**REV B**			
RESPONSE (RECIST 1.1)	PD	NE	NE	Baseline images not available for revision	INT-011, NIG-009, NIG-012, NIG-013, NIG-014	Not revised
	PD	PD	PD	NONE (full concordance)	INT-001, INT-009, IOV-004	None
	SD	SD	SD	NONE (full concordance)	CAN-002, INT-015, INT-021, INT-024, IOV-002, IOV-007, NIG-002, NIG-005	None
	PR	PR	PR	NONE (full concordance)	INT-005, INT-013, NIG-006, NIG-015	None
	SD	SD	PD	Reviewer B discordant with Investigator and A	IOV-006	To be reconciled
	SD	PR	PR	Reviewer A and B discordant with Investigator	NIG-004	To be reconciled
	PR	SD	SD	Reviewer A and B discordant with Investigator	IOV-010, NIG-003	To be reconciled
	PR	PR	SD	Reviewer B discordant with Investigator and A	IOV-003, NIG-001	To be reconciled
	PR	SD	PR	Reviewer A discordant with Investigator and B	IOV-009	To be reconciled

Table 2: Discrepancies in the evaluation of dates for time-dependent variables calculation

	INV	REV A	REV B	LEVEL OF DISCREPANCY	Patient ID #	ACTION
TIME DEPENDENT VARIABLES	Time to best response	longer	longer	Reviewer A and B discordant with Investigator	NIG-004	To be reconciled

	INV	REV A	REV B	CONCORDANCE	Patient ID #	ACTION
**TIME DEPENDENT VARIABLES**	Progression free survival	NE	NE	Baseline images not available for revision	INT-011, NIG-009, NIG-012, NIG-013, NIG-014	Not revised
		NE	NE	Clinical PD, progression images not available for revision	IOV-010	Not revised
		same	same	NONE (full concordance)	CAN-002, INT-001, INT-005, INT-009, INT-021, INT-024, IOV-002, IOV-004, IOV-009, NIG-003, NIG-004, NIG-005	None
		shorter	shorter	Reviewer A and B discordant with Investigator	INT-015, IOV-003, IOV-007, NIG-002, NIG-006, NIG-015	To be reconciled
		shorter	same	Reviewer A discordant with Investigator and B	NIG-001	To be reconciled
		same	shorter	Reviewer B discordant with investigator and A	IOV-006	To be reconciled
		same	longer	Reviewer B discordant with Investigator and A	INT-013	To be reconciled

Table 3: Reconciliation results – BEST response by RECIST

Revised Patients N 22		OBJECTIVE RESPONSE POST- REVISION			
		PD	SD	PR	CR
OBJECTIVE RESPONSE PRE- REVISION	PD	N=3			
	SD		N=9	N=1	
	PR		N=2	N=7	
	CR				N=0

Table 4: Reconciliation results – Time-dependent variables

TTP after review			
**Patients N 22**	**shorter**	**same**	**longer**
**TTP before review**	N=7	N=15	None
**DOR after review**			
**Patients N 8**	**shorter**	**same**	**longer**
**DOR before review**	N=4	N=4	None

4.1. Summary of the reconciliation results by patient

Patient INT-013
Discrepancy: Date of progression
INV: PD on 25/09/20.
REV A: PD on 25/09/20.
REV B: PR on 25/09/20.
Reconciliation: PD on 25/09/20.

Patient INT-015
Discrepancy: Date of progression
INV: PD on 16/06/20.
REV A: PD on 09/06/20.
REV B: no PD on 09/06/20.
Reconciliation: PD on 09/06/20.

Patient IOV-003
Discrepancy: Best Response and Date of progression
INV: PR on 30/05/20. PD on 23/09/2020.
REV A: PR on 30/05/20. PD on 03/08/2020.
REV B: SD on 30/05/20. PD on 03/08/2020.
Reconciliation: PR on 30/05/20. PD on 03/08/2020.

Patient IOV-006
Discrepancy: Best response and Date of progression
INV: SD on 13/08/20. PD on 09/10/2020.
REV A: SD on 13/08/20. PD on 09/10/2020.
REV B: PD on 13/08/20. PD on 13/08/20.
Reconciliation: SD on 13/08/20. PD on 09/10/2020.

Patient IOV-007
Discrepancy: Date of progression
INV: PD on 11/12/2020.
REV A: PD on 06/10/2020.
REV B: PD on 06/10/20.
Reconciliation: PD on 06/10/2020.

Patient IOV-009
Discrepancy: Best response
INV: PR on 27/10/20.
REV A: SD on 27/10/20.
REV B: PR on 27/10/20.
Reconciliation: PR on 27/10/20.

Patient IOV-010
Discrepancy: Best response
INV: PR on 23/11/20. Clinical progression on 24/12/20.

REV A: SD on 23/11/20. PD not evaluated, no images available at progression.
REV B: SD on PR on 23/11/20. PD not evaluated, no images available at progression.

Reconciliation: SD on 23/11/20. PD on 24/12/20 (clinical progression)

Patient NIG-001

Discrepancy: Best response and Date of progression.

INV: PR on 24/10/19. PD on 23/04/20
REV A: PR on 24/10/19. PD on 27/02/20
REV B: SD on 24/10/19. PD on 27/02/20

Reconciliation: PR on 24/10/19. PD on 27/02/20.

Patient NIG-002

Discrepancy: Date of progression.

INV: PD on 21/09/20.
REV A: PD on 20/05/20.
REV B: PD on 20/05/20.

Reconciliation: PD on 20/05/20.

Patient NIG-003

Discrepancy: Best response

INV: PR on 16/12/19.
REV A: SD on 16/12/19.
REV B: SD on 16/12/19.

Reconciliation: SD on 16/12/19.

Patient NIG-004

Discrepancy: Best response

INV: SD on 09/01/20.
REV A: PR on 19/02/20.
REV B: PR on 19/02/20.

Reconciliation: PR on 19/02/20.

Patient NIG-006

Discrepancy: Date of progression.

INV: PD on 12/08/20.
REV A: PD on 18/06/20.
REV B: PD on 18/06/20.

Reconciliation: PD on 18/06/20.

Patient NIG-015

Discrepancy: Date of progression.

INV: PD on 28/06/21.
REV A: PD on 28/04/21.
REV B: PD on 28/04/21.

Reconciliation: PD on 28/04/21.
