# Supplementary material for: Circulating tumor DNA to guide rechallenge with panitumumab in metastatic colorectal cancer: the phase 2 CHRONOS trial
Source: Nat Med. 2022 Aug 1;28(8):1612–8. doi: 10.1038/s41591-022-01886-0 (PMC9386661; doi:10.1038/s41591-022-01886-0)
Supplement: Supplementary file 2 — Reporting Summary [file 41591_2022_1886_MOESM2_ESM.pdf]

nature portfolio

Corresponding author(s): Prof. Alberto Bardelli

Last updated by author(s): Apr 30, 2022

Reporting Summary

Nature Portfolio wishes to improve the reproducibility of the work that we publish. This form provides structure for consistency and transparency in reporting. For further information on Nature Portfolio policies, see our Editorial Policies and the Editorial Policy Checklist.

Statistics

For all statistical analyses, confirm that the following items are present in the figure legend, table legend, main text, or Methods section.

n/a	Confirmed	
☐	☒	The exact sample size (*n*) for each experimental group/condition, given as a discrete number and unit of measurement
☒	☐	A statement on whether measurements were taken from distinct samples or whether the same sample was measured repeatedly
☐	☒	The statistical test(s) used AND whether they are one- or two-sided<br>*Only common tests should be described solely by name; describe more complex techniques in the Methods section.*
☐	☒	A description of all covariates tested
☐	☒	A description of any assumptions or corrections, such as tests of normality and adjustment for multiple comparisons
☐	☒	A full description of the statistical parameters including central tendency (e.g. means) or other basic estimates (e.g. regression coefficient) AND variation (e.g. standard deviation) or associated estimates of uncertainty (e.g. confidence intervals)
☐	☒	For null hypothesis testing, the test statistic (e.g. *F*, *t*, *r*) with confidence intervals, effect sizes, degrees of freedom and *P* value noted<br>*Give P values as exact values whenever suitable.*
☒	☐	For Bayesian analysis, information on the choice of priors and Markov chain Monte Carlo settings
☒	☐	For hierarchical and complex designs, identification of the appropriate level for tests and full reporting of outcomes
☒	☐	Estimates of effect sizes (e.g. Cohen's *d*, Pearson's *r*), indicating how they were calculated

Our web collection on statistics for biologists contains articles on many of the points above.

Software and code

Policy information about availability of computer code

Data collection	An electronic Case Report Form (CRF) designed exploiting OpenClinica platform (https://www.openclinica.com/) was used to collect clinical data.
Data analysis	Telemis version 4.9 software to collect, store, and guide the revision of the CT scan imaging results. Statistical analysis were performed with the use of SAS statistical software, version 9.4. The ddPCR data were analyzed with QuantaSoft analysis software (Bio-Rad). All libraries for circulating tumor DNA analysis were sequenced on Nextseq500 sequencer (Illumina). The bioinformatic analysis were porfermed using Ion Torrent platform-specific pipeline software (Torrent Suite™ Software 5.12, Thermo Fisher Scientific, Inc.) and the Ion Reporter Software (v. 5.10.5.0) (Thermo Fisher Scientific, Inc.) and analyzed by the Oncomine OCAv3 w3.0 - DNA - Single Sample (v. 5.10).

For manuscripts utilizing custom algorithms or software that are central to the research but not yet described in published literature, software must be made available to editors and reviewers. We strongly encourage code deposition in a community repository (e.g. GitHub). See the Nature Portfolio guidelines for submitting code & software for further information.

Data

Policy information about availability of data

All manuscripts must include a data availability statement. This statement should provide the following information, where applicable:

- Accession codes, unique identifiers, or web links for publicly available datasets
- A description of any restrictions on data availability
- For clinical datasets or third party data, please ensure that the statement adheres to our policy

Human sequencing data are available with PRJEB49484 code (EGA; https://www.ebi.ac.uk/ega/home).

Field-specific reporting

Please select the one below that is the best fit for your research. If you are not sure, read the appropriate sections before making your selection.

☒ Life sciences ☐ Behavioural & social sciences ☐ Ecological, evolutionary & environmental sciences

For a reference copy of the document with all sections, see nature.com/documents/nr-reporting-summary-flat.pdf

Life sciences study design

All studies must disclose on these points even when the disclosure is negative.

Sample size	We used the A'Hern one-stage approach to calculate the sample size. For the primary objective of the trial, 27 patients were required in order to achieve a power of at least 85% to test the null hypothesis that the rate of response to panitumumab would be 10% or less, versus the alternative hypothesis that the response rate would be 30% or more, at a one-sided alpha level of 0.05. Six objective responses were necessary to declare the study positive.
Data exclusions	No data were excluded from the analyses.
Replication	The number of times each experiment has been repeated with similar results is stated in each figure legend or in the methods section.
Randomization	No randomization was performed given that CHRONOS is is an open label, single-arm, multiple centers, phase II trial designed to evaluate the efficacy of rechallenging with panitumumab a population of RAS/BRAF wild type mCRC patients selected on the basis of absence of RAS, BRAF and EGFR ECD resistance mutations in ctDNA at the actual moment of treatment initiation. We used the A'Hern one-stage approach to calculate the sample size. For the primary objective of the trial, 27 patients were required in order to achieve a power of at least 85% to test the null hypothesis that the rate of response to panitumumab would be 10% or less, versus the alternative hypothesis that the response rate would be 30% or more, at a one-sided alpha level of 0.05. Six objective responses were necessary to declare the study positive.
Blinding	At clinical standpoint, no blinding was required given that CHRONOS is is an open label, single-arm, multiple centers, phase II trial designed to evaluate the efficacy of rechallenging with panitumumab a population of RAS/BRAF wild type mCRC patients selected on the basis of absence of RAS, BRAF and EGFR ECD resistance mutations in ctDNA at the actual moment of treatment initiation. Concerning translational analyses, bioinformatics and pathologists involved were not aware and blinded of patients' outcome at the time of data analyses. Translational data and patients' outcome were matched once the former were completed.

Reporting for specific materials, systems and methods

We require information from authors about some types of materials, experimental systems and methods used in many studies. Here, indicate whether each material, system or method listed is relevant to your study. If you are not sure if a list item applies to your research, read the appropriate section before selecting a response.

Materials & experimental systems

n/a	Involved in the study	
☒	☐	Antibodies
☒	☐	Eukaryotic cell lines
☒	☐	Palaeontology and archaeology
☒	☐	Animals and other organisms
☐	☒	Human research participants
☐	☒	Clinical data
☒	☐	Dual use research of concern

Methods

n/a	Involved in the study	
☒	☐	ChIP-seq
☒	☐	Flow cytometry
☒	☐	MRI-based neuroimaging

Human research participants

Policy information about studies involving human research participants

Population characteristics	A total of 52 patients were screened and 27 with no detectable alterations in RAS, BRAF, and EGFR ECD on ctDNA were enrolled for anti-EGFR treatment (Figure 1 and Supplementary Figure 2). The patients' characteristics are representative of a third or further line population of mCRC patients (Table 1). The median number of previous anticancer treatments was 3 and the previous anti-EGFR treatment most commonly administered was panitumumab (15/27, 55%), predominantly in 1st line (21/27, 78%) and always in combination with a cytotoxic backbone. All patients had a microsatellite stable (MSS) tumor. The primary endpoint of the trial was overall response rate (ORR) to panitumumab rechallenge according to RECIST version 1.1 criteria 25, while the secondary endpoints were progression-free survival (PFS), OS and toxicity according to Common Terminology Criteria for Adverse Events (CTCAE) version 4.03.
Recruitment	The main inclusion criteria were: histologically confirmed metastatic CRC with RAS and BRAF wild-type status of the primary colorectal cancer and/or related metastasis; objective response and subsequent documented progression upon a previous

anti-EGFR therapy-based regimen administered in any line of treatment; intervening anti-EGFR-free treatment; selection on the basis of RAS, BRAF and EGFR ECD WT status in ctDNA at molecular screening after progression (within 4 weeks) to the last anti-EGFR-free regimen (Supplementary Figure 2). Further criteria were: older than 18 years of age; an Eastern Cooperative Oncology Group performance status of 0 to 2; measurable metastatic disease according to the Response Evaluation Criteria in Solid Tumors, version 1.1. The trial was conducted in accordance with the Declaration of Helsinki 44 and adhered to the international Good Clinical Practice guidelines. The protocol was approved by local ethics committees of participating sites (Niguarda Cancer Center, Grande Ospedale Metropolitano Niguarda, Milano, Italy; Fondazione IRCCS Istituto Nazionale dei Tumori, Milan, Italy; Veneto Institute of Oncology (IOV)-IRCCS Padua, Italy; Istituto di Candiolo, Fondazione del Piemonte per l'Oncologia, FPO-IRCCS, Candiolo, Italy; Policlinico Universitario Biomedico, Roma, Italy; HUMANITAS Research Hospital, Milano, Italy). All patients provided written informed consent to study procedures. Due to the outbreak of the COVID-19 pandemic, and the resulting travel restrictions established by the Italian government, in three patients treatment was partially delivered in a hospital different from the initial recruiting center.
The CHRONOS' strategy has limitations and can be further improved. First, the 3-genes ddPCR panel molecular screening implemented in CHRONOS could be further refined. In this regard, future studies should consider screening of a larger panel of resistant variants in ctDNA to increase the therapeutic index of anti EGFR monoclonal antibodies. For example, assessment of MAPK alterations or ERBB2/MET amplification should also be considered as these are similarly known to confer resistance to EGFR blockade although at lower prevalence in mCRC 29,34–39. Second, the assessment of anti-EGFR resistance-conferring mutations on ctDNA requires a dedicated ctDNA analysis laboratory support. As liquid biopsies become more routinely deployed this issue should progressively fade thanks to the large number of certified laboratories which now offer rapid ctDNA testing 40,41. Third, we are unable to precisely estimate whether stochastic mutational events could have affected the sensitivity of detecting variants by ddPCR or NGS.

STUDY POPULATION
8.1 Inclusion Criteria
1 Histologically confirmed diagnosis of metastatic colorectal cancer;
2 Age ≥ 18 years;
3 Written informed consent;
4 Documented WT RAS exons 2, 3 and 4 (KRas and NRas) and WT BRAF V600E for anti-EGFR treatment.
5 Complete or partial response to anti EGFR antibodies in any line either received as monotherapy or in combination with chemotherapy;
6 Imaging documented progression while on therapy with a therapeutic regimen including anti-EGFR mAb;
7 Imaging documented progression at the last treatment regimen that must be anti-EGFR free;
8 Patient must be RAS and EGFR ectodomain wild type in a liquid biopsy performed no longer that 4 weeks after progression to the last anti-EGFR free treatment
9 FFPE sample used for eligibility to anti-EGFR prescription (see criteria 4) must be available for custom gene panel profiling (as described in appendix B). Otherwise if sample is not available, center must have already perfomed a genotyping on this tissue sample according to appendix B.
10 ECOG performance status ≤ 2;
11 At least one measurable tumor lesion as per RECIST v1.1. Lesions in previously irradiated areas or those that have received other loco-regional therapies (i.e. percutaneous ablation) should not be considered measurable unless there is clear documented evidence of progression of the lesion since therapy. Imaging must be performed maximum within 28 days prior to registration;
12 Normal organ functions;
13 Negative serum pregnancy test within 1 week prior to the first study dose in all women of childbearing potential;
14 Subjects and their partners must be willing to avoid pregnancy during the trial. Male subjects with female partners of childbearing potential and female subjects of childbearing potential must, therefore, be willing to use adequate contraception;
15 Absence of any psychological, familial, sociological or geographical condition potentially hampering compliance with the study protocol and follow-up schedule; those conditions should be discussed with the patient before registration in the trial.
8.2 Exclusion Criteria
1. History of severe infusion reactions to monoclonal antibodies cetuximab or panitumumab;
2. Symptomatic or untreated leptomeningeal disease and symptomatic brain metastasis;
3. Clinically significant cardiac disease including:
a. congestive heart failure requiring treatment (NYHA grade ≥ 2), Left ventricular ejection fraction (LVEF) < 45% as determined by Multigated acquisition (MUGA) scan or echocardiogram;
b. history or presence of clinically significant ventricular arrhythmias or atrial fibrillation;
c. clinically significant resting bradycardia;
d. unstable angina pectoris ≤ 3 months prior to starting study drug;
e. acute myocardial infarction ≤ 3 months prior to starting study drug;
f. QTcF > 480 msec;
4. History of thromboembolic or cerebrovascular events within the last 6 months, including transient ischemic attack, cerebrovascular accident, deep vein thrombosis, or pulmonary embolism;
5. Patients with interstitial pneumonitis or pulmonary fibrosis;
6. Abnormal organ or bone marrow functions defined as:
a. Absolute neutrophil count < 1.5 x 10/L;
b. hemoglobin < 9 g/dL;
c. alkaline phosphatase > 2.5 x upper normal limit (ULN), if liver metastases > 5 x ULN;
d. aspartate aminotransferase (AST)/ alanine aminotransferase (ALT) > 2.5 x ULN, if liver metastases > 5 x ULN;
e. bilirubin > 1.5 x ULN, if liver metastases > 2 x ULN;
f. serum creatinine > 1.5 x ULN and/or creatinine clearance ≤ 50 mL/min calculated according to Cockroft-Gault;
g. Patients with platelet count <100 x 10^9/L
7. Previous or concurrent second malignancy. Exceptions: adequately treated basal cell or squamous cell skin cancer; in situ carcinoma of the cervix, treated curatively and without evidence of recurrence for at least 3 years prior to study entry; or other solid tumor treated curatively and without evidence of recurrence for at least 3 years prior to study entry.
8. Patients with positive serology for HIV, HBV, HCV.
9. Patients with a history of severe or life threatening hypersensitivity to the active substance or to any of the excipients.

8.3 Patients substitution criteria
8.3.1 Severe Infusion reaction (Trial Phase only)
Patient experiencing severe infusion reaction to panitumumab requiring permanent drug discontinuation will be substituted.

Ethics oversight	The protocol was approved by local ethics committees of participating sites (Niguarda Cancer Center, Grande Ospedale Metropolitano Niguarda, Milano, Italy; Fondazione IRCCS Istituto Nazionale dei Tumori, Milan, Italy; Veneto Institute of Oncology (IOV)-IRCCS Padua, Italy; Istituto di Candiolo, Fondazione del Piemonte per l'Oncologia, FPO-IRCCS, Candiolo, Italy; Policlinico Universitario Biomedico, Roma, Italy; HUMANITAS Research Hospital, Milano, Italy). All patients provided written informed consent to study procedures.

Note that full information on the approval of the study protocol must also be provided in the manuscript.

Clinical data

Policy information about clinical studies

All manuscripts should comply with the ICMJE guidelines for publication of clinical research and a completed CONSORT checklist must be included with all submissions.

Clinical trial registration	EudraCT 2016-002597-12, NCT03227926
Study protocol	For more details please refer to the full protocol detailed in the Supplementary Material Appendix 1.
Data collection	Patients were enrolled at Niguarda Cancer Center, Grande Ospedale Metropolitano Niguarda, Milano, Italy; Fondazione IRCCS Istituto Nazionale dei Tumori, Milan, Italy; Veneto Institute of Oncology (IOV)-IRCCS Padua, Italy; Istituto di Candiolo, Fondazione del Piemonte per l'Oncologia, FPO-IRCCS, Candiolo, Italy. The first was enrolled on August 18th 2019 while the last patient was enrolled on November 6th 2020. The study ended on December 31st 2021.
Outcomes	Tumor assessments were performed by local radiologists within 4 weeks before treatment start (baseline) and were repeated every 8 weeks according to RECIST version 1.1 thereafter until progression. Local tumors assessments were reviewed centrally by two radiologists (D. R., A. V.) who read the CT scans blinded using the Telemis version 4.9 software to collect, store, and guide the revision of the imaging results. The imaging review protocol and tumor assessment reconciliation report are included in Supplementary Material Appendix 5. Safety was continuously assessed and graded according to the Common Terminology Criteria for Adverse Events (CTCAE), version 4.03. (Protocol Supplementary Material Appendix 1).<br>We used the A'Hern one-stage approach to calculate the sample size. For the primary objective of the trial, 27 patients were required in order to achieve a power of at least 85% to test the null hypothesis that the rate of response to panitumumab would be 10% or less, versus the alternative hypothesis that the response rate would be 30% or more, at a one-sided alpha level of 0.05. Six objective responses were necessary to declare the study positive.<br>Secondary endpoints were PFS and OS, as well as safety given that panitumumab is routinely administered to CRC patients. Translational exploratory objectives were aimed at studying the molecular determinants of response and resistance to study treatment and included molecular characterization of longitudinal liquid biopsies collected during the treatment. Time-to-event variables were estimated using the Kaplan-Meier method and log-rank test was used for testing the null hypothesis of no difference among curves. Analyses were performed with the use of SAS statistical software, version 9.4.
